# Spatial single-cell proteotyping reveals immunotherapy-resistant features within the complex tumor microenvironment of metastatic NSCLC

**DOI:** 10.1172/JCI195021

**Published:** 2026-03-10

**Authors:** Kohsuke Isomoto, Koji Haratani, Takahiro Tsujikawa, Shuta Tomida, Yusuke Makutani, Masayuki Takeda, Kimio Yonesaka, Kaoru Tanaka, Tsutomu Iwasa, Kazuko Sakai, Kazuto Nishio, Akihiko Ito, Kazuhiko Nakagawa, Hidetoshi Hayashi

**Affiliations:** 1 Department of Medical Oncology, Kindai University Faculty of Medicine, Osaka-Sayama, Japan.; 2 Department of Medical Oncology, Dana-Farber Cancer Institute, Boston, Massachusetts, USA.; 3 Department of Otolaryngology–Head and Neck Surgery, Kyoto Prefectural University of Medicine, Kyoto, Japan.; 4 Center for Comprehensive Genomic Medicine, Okayama University Hospital, Okayama, Japan.; 5 Department of Surgery,; 6 Department of Genome Biology, and; 7 Department of Pathology, Kindai University Faculty of Medicine, Osaka-Sayama, Japan.

**Keywords:** Clinical Research, Immunology, Oncology, Biomarkers, Cancer immunotherapy, Lung cancer

## Abstract

**BACKGROUND:**

Immune checkpoint inhibitors (ICIs) targeting the programmed cell death 1 axis have revolutionized metastatic non–small cell lung cancer (mNSCLC) treatment. However, disease progression remains a concern, and the role of the complex tumor microenvironment (TME) in treatment failure is not fully understood.

**METHODS:**

In this biomarker study involving 103 patients with mNSCLC, including 81 patients who received ICI treatment, we evaluated the association between heterogeneous immune cell subsets and ICI efficacy through single-cell spatial profiling of pretreatment tumor tissue, using a 29-marker multiplex IHC platform built for in-depth dissection of the TME.

**RESULTS:**

Among various types of intratumoral lymphocytes, including Th1, Treg, and NK cells, only CD8^+^ T cells (tumor-infiltrating lymphocytes [TILs]) were associated with ICI efficacy. Computational tissue segmentation underscored the importance of direct physical interactions between CD8^+^ TILs and cancer cells for ICI efficacy. TIL phenotyping identified CD39/CD103/Ki-67 positivity as a hallmark of exhausted yet functional tumor-reactive CD8^+^ TILs. Immunosuppressive tumor-associated macrophages (TAMs) and cancer-associated fibroblasts were independent unfavorable adversaries. High CD73 expression on cancer cells was suggested to confer tolerance to ICI in *EGFR/ALK*-oncogene^+^ NSCLC, potentially through M2-TAM accumulation and aberrant angiogenesis.

**CONCLUSION:**

Our study delineates the clinical relevance of heterogeneous immune cell subsets in ICI-treated mNSCLC, aiding the development of targeted therapeutic strategies.

**FUNDING:**

Osaka Cancer Society, KANAE Foundation for the Promotion of Medical Science, SGH Foundation, and YOKOYAMA Foundation for Clinical Pharmacology.

## Abstract 2

Spatial single-cell proteotyping of 103 stage IV NSCLC samples reveals that exhausted yet functional tumor-reactive CD8^+^ T cells in direct contact with cancer cells enhance ICI efficacy, while M2-TAMs, CAFs, and the CD73-adenosine pathway drive ICI resistance in metastatic NSCLC.

## Graphical abstract

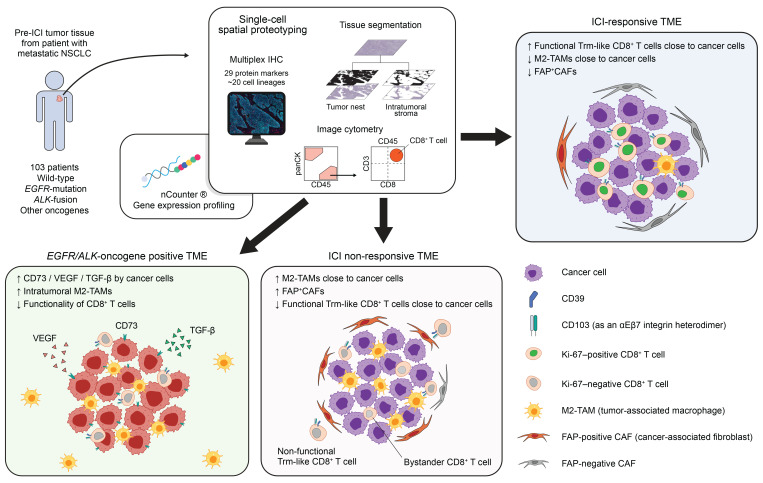

## Introduction

Immune checkpoint inhibitors (ICIs) have become a cornerstone of treatment for various solid malignancies (1). Despite such a recent development of immunotherapy, non–small cell lung cancer (NSCLC) remains a leading cause of cancer-related death because most patients with metastatic NSCLC (mNSCLC) have not responded sufficiently to the current ICI treatment (2). Over two-thirds of patients experience early disease progression within 1 year during programmed cell death 1 (PD-1) or programmed cell death ligand 1 (PD-L1) inhibitor monotherapy in first- or second-line treatment settings according to previous large clinical trials (ClinicalTrials.gov KN-042, CheckMate017, and CheckMate057) (3, 4). In preselected treatment-naive populations with a high likelihood of a favorable response to PD-1/PD-L1 inhibitor monotherapy due to high PD-L1 expression in cancer cells (PD-L1 tumor proportion score [TPS] ≥ 50%), over half of patients experienced early disease progression (KN-024) (5). To overcome the early resistance to PD-1/PD-L1 inhibitors, researchers have developed combined treatment regimens, such as pairing PD-1/PD-L1 inhibitors with cytotoxic lymphocyte antigen-4 antibody or cytotoxic chemotherapy. These regimens may also include VEGF signal blockade to enhance therapeutic efficacy (6–8). While combination therapies have achieved marginally improved survival outcomes, over half of patients still face the prospect of early resistance, even with these aggressive and toxic treatments. Given the limited clinical successes and the complexities encountered in the recent development of new immunotherapy strategies for mNSCLC, a deeper understanding of the tumor microenvironment (TME) is crucial for effectively stratifying patients, optimizing current care, and developing a focused and efficient next-generation treatment strategy (9).

PD-L1 protein expression in cancer cells is a common clinical biomarker associated with PD-1/PD-L1 inhibitor efficacy in mNSCLC. However, its limited utility is acknowledged due to insufficient correlation between PD-L1 TPS and survival outcomes (10). Recent well-designed clinical studies aimed at consistently overcoming this limitation highlighted the independent importance of preexisting tumor-infiltrating lymphocytes (TILs) for subsequent antitumor efficacy with PD-1/PD-L1 blockade therapy in mNSCLC (11–14). These studies used different biomarkers, such as simple CD8^+^ TIL density estimated by conventional IHC (11), tumor-infiltrating pan-lymphocytic cell density determined by artificial intelligence (AI) technology based on cellular morphology in H&E staining of tumor tissue (12, 13), or transcriptomic signatures suggesting immune cell infiltration in bulk tumor samples (14). All of these biomarkers exhibited a consistent association with better treatment efficacy, confirming the significance of TILs in the pretreatment TME for anti–PD-1/PD-L1 efficacy.

However, questions remain about how heterogeneous lymphocytic cell subsets, such as Tregs and NK, NKT, Th1, and CD8^+^ T cells, each potentially playing a different role in the TME, are associated with ICI efficacy (15). Even intratumoral CD8^+^ T cells should be considered biologically heterogeneous, as suggested by previous preclinical or translational studies. For example, only a small fraction of intratumoral CD8^+^ T cells are actually tumor reactive — which could possibly be visualized by tissue-resident memory-like (Trm-like) phenotype such as CD39 and CD103 — while others are bystanders incapable of recognizing cancer cells (16). In addition, even such tumor-reactive CD8^+^ T cells eventually become terminally exhausted after repetitive antigen exposures, rendering them unable to be reinvigorated by PD-1/PD-L1 blockade (17–19). Furthermore, numerous preclinical studies have suggested the detrimental effects of nonlymphocytic immune cells, such as immunosuppressive M2-type (CD163^+^ or CD206^+^) tumor-associated macrophages (TAMs), myeloid-derived suppressor cells (MDSCs), and cancer-associated fibroblasts (CAFs), on antitumor efficacy with ICI therapy. However, their clinical significance in the context of PD-1/PD-L1 inhibitor efficacy in mNSCLC has not been fully investigated (15, 20).

Such cellular and biological heterogeneity has not been analyzed by conventional stains. Bulk or single-cell transcriptomic profiling could provide comprehensive data but lacks spatial single-cell information, offering an incomplete representation of the TME (21). Therefore, the next emerging challenge is a comprehensive and simultaneous evaluation of these heterogeneous immune cell subsets in a single-cell level context with spatial information for ICI efficacy. The recent development of state-of-the-art technologies enables spatial transcriptomic analysis; however, challenges such as insufficient microscopy resolution without single-cell level information, functionally indirect evidence like RNA, or extremely high costs remain for substantial clinical oncology research (21–23). For example, several studies using GeoMX DSP platforms evaluated protein expression in selected regions of tumor tissues and reported the association between several immune features and ICI efficacy in NSCLC, but single-cell-level resolution was not achievable (24–27). However, imaging-based platforms such as multiplex IHC (mIHC) or multiplex immunofluorescence enable spatially resolved single-cell proteotyping. To date, only a limited number of studies utilized these methods to dissect the TME in a substantial number of ICI-treated metastatic NSCLC cases (28–31). Even in these pioneering studies, however, only a limited set of markers (up to 6 protein markers) could be evaluated because profiling a larger variety of immune cells at the same space was challenging. In our previous pilot study based on a few NSCLC cases, we demonstrated the feasibility of our mIHC platform visualizing 16–17 protein markers at a single-cell level, which revealed that the presence of Trm-like CD8^+^ TILs might be important for initial ICI response but does not necessarily predict persistent tumor control (32). This preliminary work warranted further effort involving a larger cohort to explore additional TME features for sufficient ICI efficacy in mNSCLC.

Given this background, we performed a retrospective biomarker study to clarify how TME complexity dictates clinical ICI efficacy in mNSCLC. Here, we used mIHC for spatial single-cell profiling (29 protein markers from 2 serial sections), which was complemented by bulk tumor transcriptome analysis (750 immune-related genes from nCounter IO360). This allowed us to deeply dissect the TME. We prespecified key protein markers for our mIHC library on the basis of clinical questions and hypotheses mentioned above. This comprehensive strategy facilitated a profound stratification of the TME in mNSCLC, elucidating unique TME features that contribute to long-term responses or resistance to PD-1/PD-L1 blockade therapy.

## Results

### Patient characteristics and study schema.

**Figure 1:**
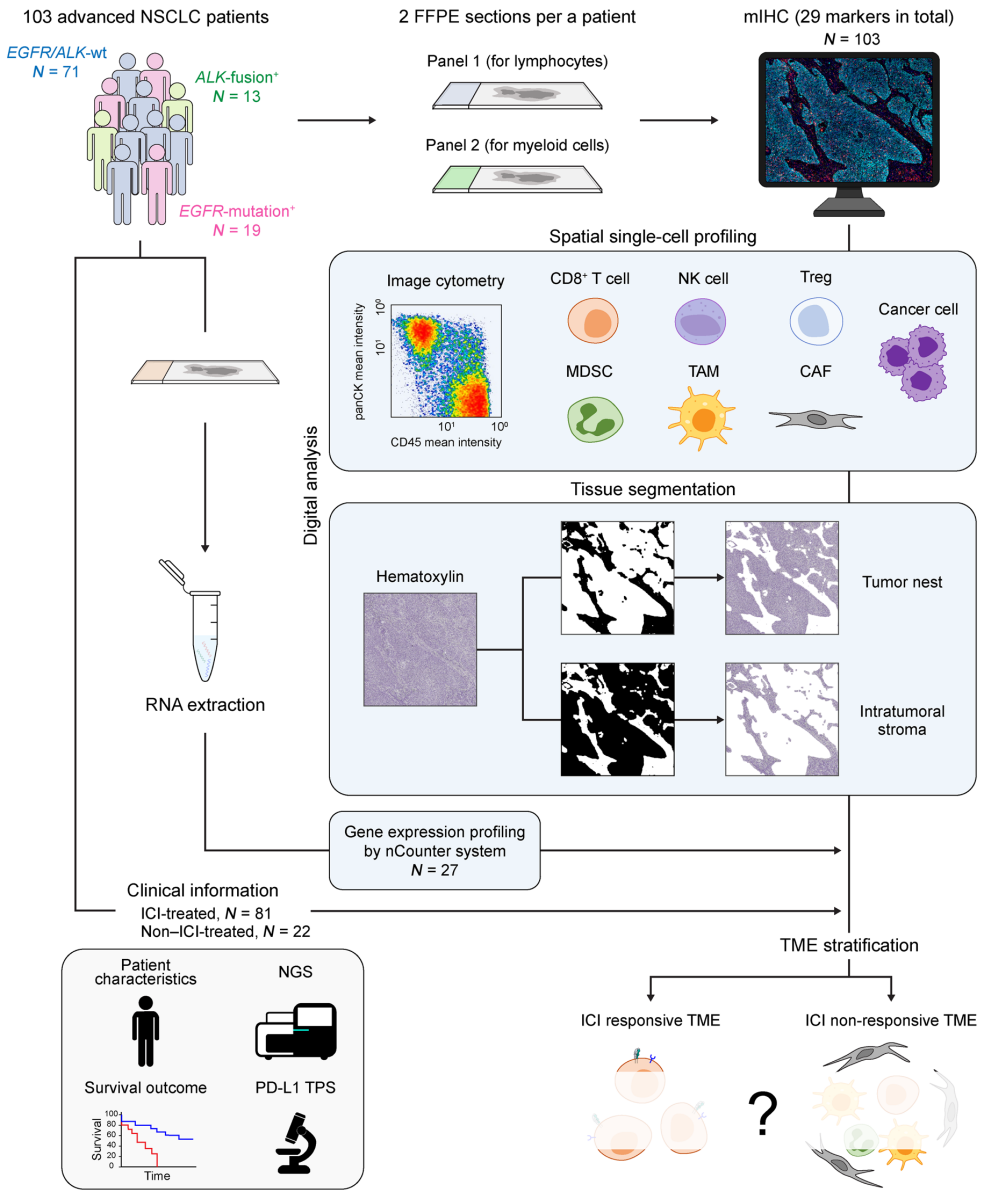
A schematic of the study design. Two sequential FFPE tumor tissue sections from 103 patients with advanced NSCLC, including those with tumors positive for *EGFR*-mutations or *ALK*-fusion genes, were subjected to mIHC. Subsequent spatial single-cell profiling using digital analysis aided in defining cell subsets in the tumor nest and intratumoral stroma. Gene expression analysis was performed via the nCounter platform if sufficient tumor tissue yielded good-quality RNA. All these data were integrated with clinical information, including survival outcomes, next-generation sequencing (NGS) results, and PD-L1 TPS to stratify ICI-responsive TME and nonresponsive TME.

A total of 103 patients were enrolled in this study (Figure 1 and Supplemental Figure 1; supplemental material available online with this article; https://doi.org/10.1172/JCI195021DS1). Seventy-one patients were negative for the *EGFR*-mutations and the anaplastic lymphoma kinase (*ALK*)-fusion genes (*EGFR/ALK*-wt), whereas 19 patients had *EGFR*-mutations, and 13 patients had *ALK*-fusion oncogenes. Eighty-one patients were treated by PD-1/PD-L1 inhibitors, including 10 patients with *EGFR*-mutations. Of the remaining 71 patients, 36 patients were screened by next-generation sequencing, where 14 patients were positive for other known driver oncogenes. Thirty-five other patients remained inconclusive for driver oncogenes. Details for clinicopathologic and genomic features of these 81 patients are provided in Supplemental Tables 1 and 2. Of these 81 patients, 2 patients experienced early censoring (<1 year) and 30 patients (37%) experienced a long-term tumor response (≥1 year) to PD-1/PD-L1 inhibitors (referred to as durable clinical benefit [DCB] hereafter). In contrast, 49 patients (61%) developed early tumor progression (<1 year) (non-DCB), showing a clear difference in progression-free survival (PFS) and overall survival (OS) between these 2 subgroups with HRs of 0.15 (95% CI, 0.09–0.26) and 0.11 (95% CI, 0.06–0.19), respectively (Supplemental Figure 2, A and B). PD-L1 TPS, a classical predictive biomarker for PD-1/PD-L1 inhibitor therapy, was not associated with PFS and OS in our cohort regardless of treatment lines or treatment regimens (monotherapy or combination therapy), consistent with the insufficient power of PD-L1 TPS alone as a single biomarker (Supplemental Figure 2, C and D), although an extremely high PD-L1 TPS, such as ≥90%, can somewhat overcome this limitation, as suggested by previous studies (Supplemental Figure 2E) (33, 34). In agreement with previous studies (35, 36), *EGFR*-mutations were associated with early disease progression during PD-1/PD-L1 inhibitor treatment (Supplemental Figure 2F). Non-*EGFR/ALK* driver oncogenes were not associated with ICI efficacy (Supplemental Figure 2F), in agreement with the prevailing view that *BRAF/KRAS/MET* oncogenes, which accounted for a majority of our cases, are ICI responsive (37, 38).

To identify TME profiles responsible for these distinct responses to PD-1/PD-L1 inhibitors in mNSCLC, we built a highly informative platform using single-cell spatial profiling of pretreatment TME provided by our mIHC system, combined with transcriptomic analysis of the tumor tissues (Figure 1). In this mIHC system, ≥20 cell subsets were visualized and quantified as developed by our recent study (32) (Supplemental Table 3).

### CD8^+^ T cells as an important lymphocytic cell subset responsible for durable response to PD-1/PD-L1 blockade therapy.

**Figure 2:**
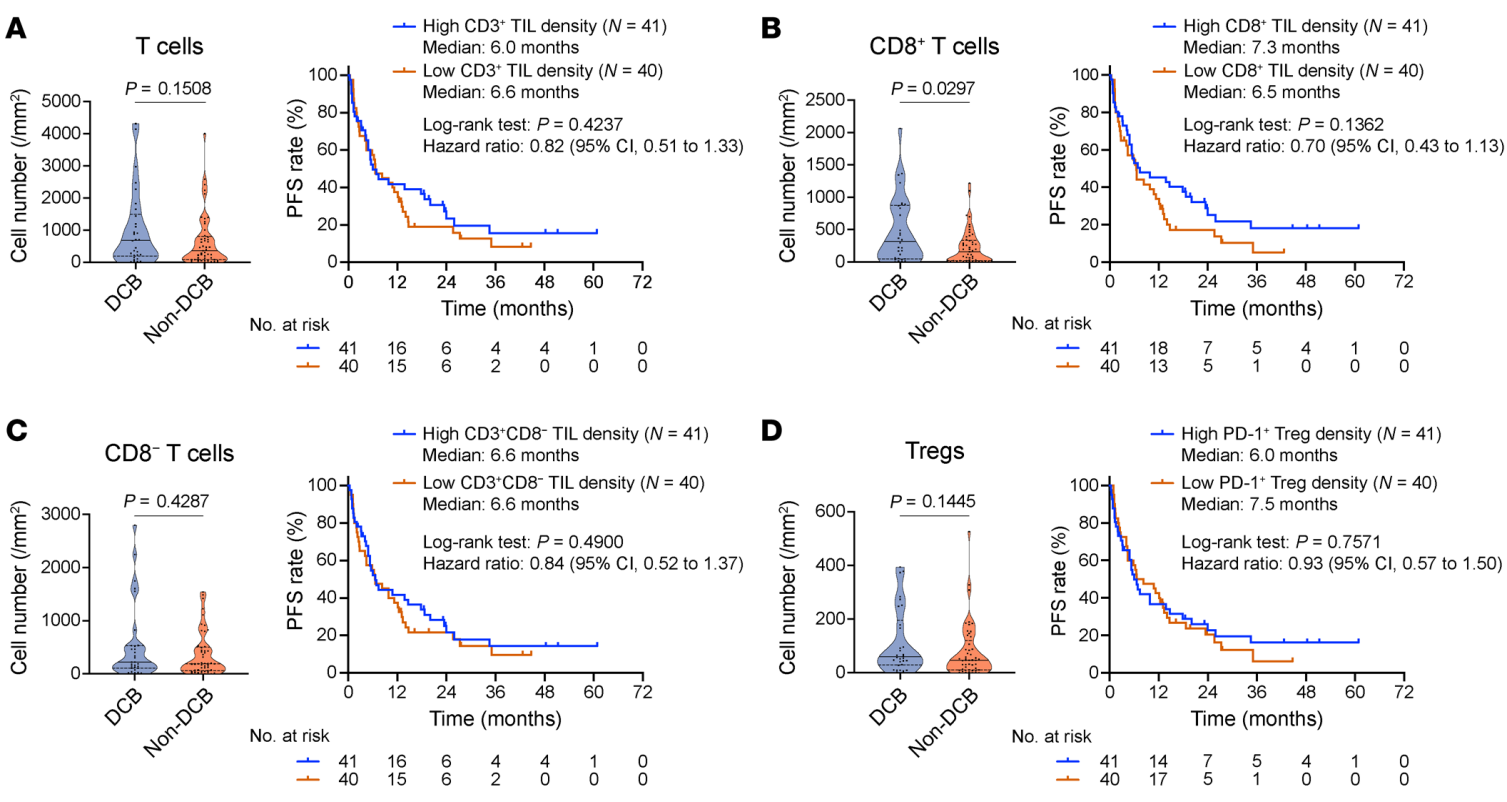
Association of intratumoral T cell density with ICI efficacy. (**A**–**C**) Densities of intratumoral T cells, CD8^+^ T cells, and CD8^–^ T cells compared between the DCB (*N* = 30) and non-DCB (*N* = 49) groups (left) and Kaplan-Meier (KM) curves for PFS of ICI treatment stratified by cell densities (right). (**D**) Intratumoral Treg densities compared between the DCB and non-DCB groups (left) and KM curves for PFS of ICI treatment stratified by PD-1^+^ Treg density (right). Data are presented in violin plots in **A**–**D**; each dot represents 1 patient, and *P* values are indicated on horizontal bars. Two cases were not included in the left panels because DCB/non-DCB status could not be determined due to early censoring (<1 year) of PFS data. Vertical bars on the KM curves indicate censoring. *P* values of the violin plots and survival analyses were determined by Mann-Whitney *U* test and log-rank test, respectively.

Our platform enabled a precise quantification of different lymphocyte subsets, including CD8^+^ T cells, Tregs, and NK cells, in the same spaces (Supplemental Figure 3A). Although a prior AI-based evaluation of H&E stains of mNSCLC using large databases validated an association between intratumoral lymphocyte infiltration and improved ICI efficacy (12, 13), tumor-infiltrating T cell density only was not sufficient to predict a better response to PD-1/PD-L1 blockade therapy in our cohort (Figure 2A). Indeed, PFS curves did not exhibit a marked difference based on T cell density (Figure 2A). Further categorization of these T cells revealed that only CD8^+^ T cells were more infiltrated in tumors from patients with DCB, whereas non-CD8^+^ T cells were not entirely associated with the efficacy (Figure 2, B and C). Additionally, evaluation of these non-CD8^+^ T cells indicated that Th1 and NKT cells were not associated with the treatment efficacy (Supplemental Figure 3B). While Tregs were anticipated to counteract ICI response based on their immunosuppressive role (39, 40), our findings indicate that these cells were not clearly associated with early resistance to PD-1/PD-L1 inhibition. This observation held true even after considering the expression of PD-1 on Tregs (Figure 2D). The intratumoral cell density for non-T lymphocytes, such as NK and B cells, was similar based on the treatment efficacy (Supplemental Figure 3C).

These data suggest that CD8^+^ T cells are the most crucial tumor-infiltrating immune cell populations for enhancing immune cell–based cancer cell killing in response to clinical PD-1/PD-L1 inhibition.

### Crucial spatial proximity: CD8^+^ T cells’ contact with cancer cells for durable PD-1/PD-L1 blockade response.

**Figure 3:**
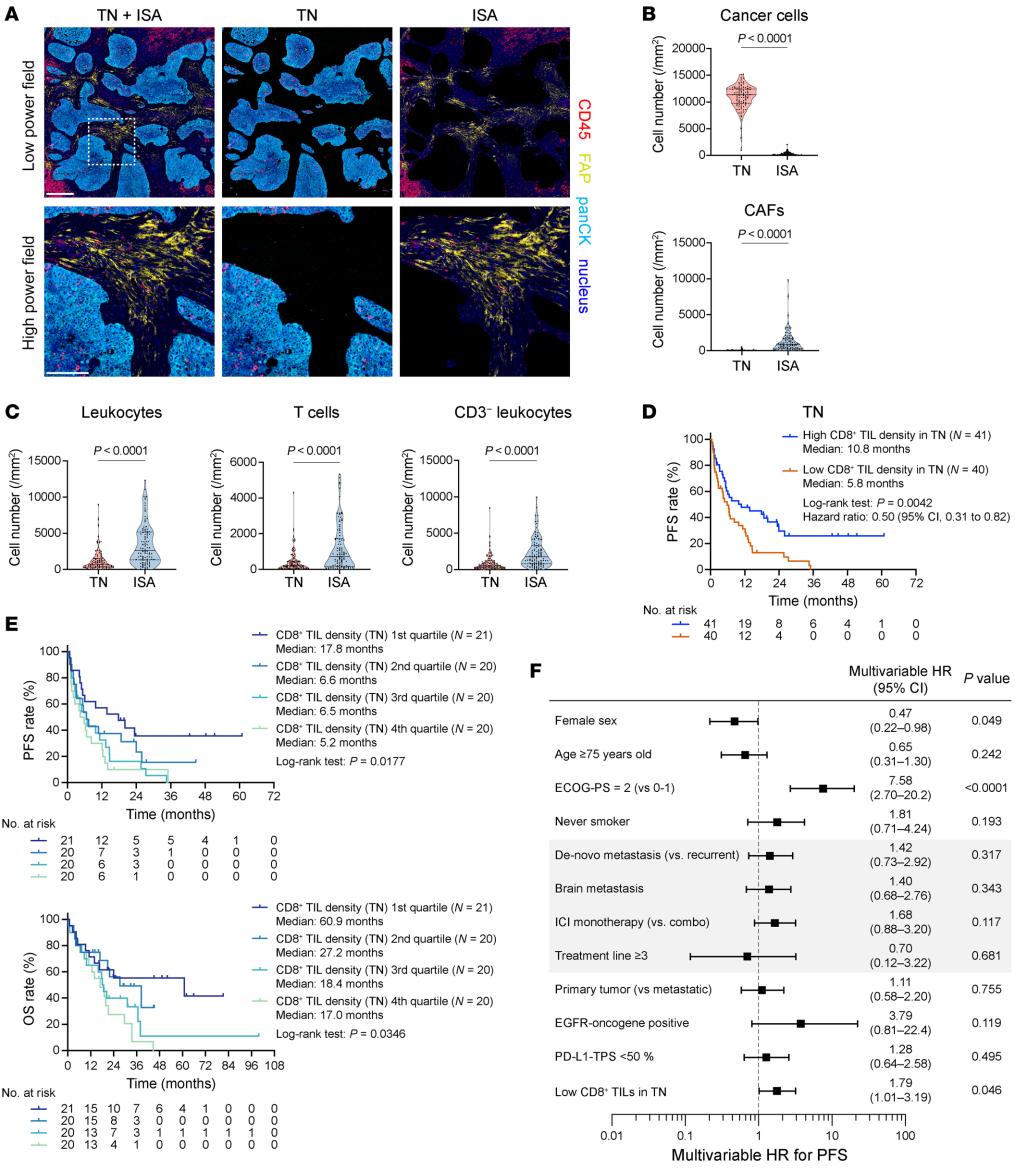
Association of spatial localization of tumor-infiltrating CD8^+^ T cells with ICI efficacy. (**A**) Representative mIHC images of the tumor tissues indicate that the tissue segmentation method confidently separates TN and ISA. Multicolor images of TN+ISA, TN, and ISA — with nucleus (blue), panCK (cyan), CD45 (red), and FAP (yellow) — are shown in the left, middle, and right columns, respectively. The boxed region is shown at a higher magnification in the bottom row. Scale bars: 300 μm (top), 100 μm (bottom). (**B**) Cell numbers of cancer cells (top) and CAFs (bottom) are compared between TN and ISA (*N* = 103). (**C**) Cell numbers of leukocytes (CD45^+^), T cells (CD45^+^CD3^+^), and CD3^−^ leukocytes (CD45^+^CD3^−^) in TN and ISA (*N* = 103). (**D**) KM curves for PFS of ICI treatment are compared according to CD8^+^ TIL densities in TN. (**E**) KM curves for PFS (top) and OS (bottom) are compared according to CD8^+^ TIL densities in TN based on quartiles. (**F**) A forest plot of HRs estimated by multivariable Cox proportional hazards regression models for evaluation of the association of PFS for ICI treatment with the indicated biomarkers. Data are presented in violin plots in **B** and **C**, with each dot representing 1 patient. *P* values of the violin plots and survival analyses were determined by Mann-Whitney *U* test and log-rank test, respectively. Vertical bars on KM curves indicate censoring. ECOG-PS, Eastern Cooperative Oncology Group performance status.

Since intratumoral CD8^+^ T cell density did not sufficiently identify long-term responders, we further dissected a profile related to CD8^+^ TILs to identify more predictive features. Our digital pathology technology separated intratumoral regions into tumor cell nest area (TN) and intratumoral stromal area (ISA). Notably, cancer cells (pan-cytokeratin [panCK]^+^CD45^−^) were almost exclusively distributed in the TN area, whereas only the ISA harbored CAFs (panCK^−^CD45^−^FAP^+^) (Figure 3, A and B). Leukocytes or T lymphocytes were distributed in both regions, with a higher distribution in the ISA, suggesting a lesser tendency for immune cells to infiltrate into cancer cell nests, particularly for non-T leukocytes including myeloid, B, and NK cells (Figure 3C).

Using this technology, we tested the importance of the spatial proximity of CD8^+^ TILs to cancer cells. Consequently, a higher density of CD8^+^ TILs in the TN was clearly associated with long-term responses to PD-1/PD-L1 blockade therapy (Figure 3D), highlighting the significance of physical contact between CD8^+^ TILs and cancer cells. Of note, a dose–response relationship between ICI efficacy and the CD8^+^ TIL density in TN was suggested, as shown in a subgroup analysis based on quartile values (Figure 3E). However, non-CD8^+^ lymphocytes (Th1, NK, NKT, and Tregs) were not associated with tumor response even with closer proximity to cancer cells (Supplemental Figure 4A). Of note, in agreement with the recent report that tumor-reactive CD8^+^ TILs colocalize with CD4^+^ T cells and Tregs at cancer cell nest-forming immunity hubs (41), densities of Th1 and Tregs were also strongly correlated with those of CD8^+^ TILs in TN in our study (Supplemental Figure 4B). Given that only CD8^+^ TIL density was predictive of ICI efficacy, however, the co-infiltration of such non-CD8^+^ T cells at the CD8^+^ TIL–cancer cell interface ultimately may not be crucial for ICI-induced antitumor efficacy.

To investigate why PD-L1 TPS was not strongly associated with ICI efficacy, we evaluated its relationship with CD8^+^ TIL density. PD-L1 staining on cancer cells has been regarded as a surrogate marker of IFN pathway activation, mainly driven by IFN-γ from cancer cell targeting CD8^+^ T cells (42). However, PD-L1 TPS did not accurately reflect CD8^+^ T cell infiltration in our cohort (Supplemental Figure 4C).

We performed a multivariable analysis considering heterogeneous clinical features, particularly for different treatment regimens (monotherapy or combination therapy) and treatment lines (early or late lines). We confirmed that the CD8^+^ TIL density in TN stands as an independent factor for ICI efficacy, irrespective of treatment backgrounds (HR, 1.79 [95% CI, 1.01–3.19]) (Figure 3F). However, such association became less apparent in the same multivariable analysis using CD8^+^ TIL density in ISA, reemphasizing the importance of spatial proximity between antitumor CD8^+^ TILs and cancer cells (Supplemental Figure 4D).

### Features of exhausted yet functional CD8^+^ TILs linked to improve PD-1/PD-L1 inhibition response.

**Figure 4:**
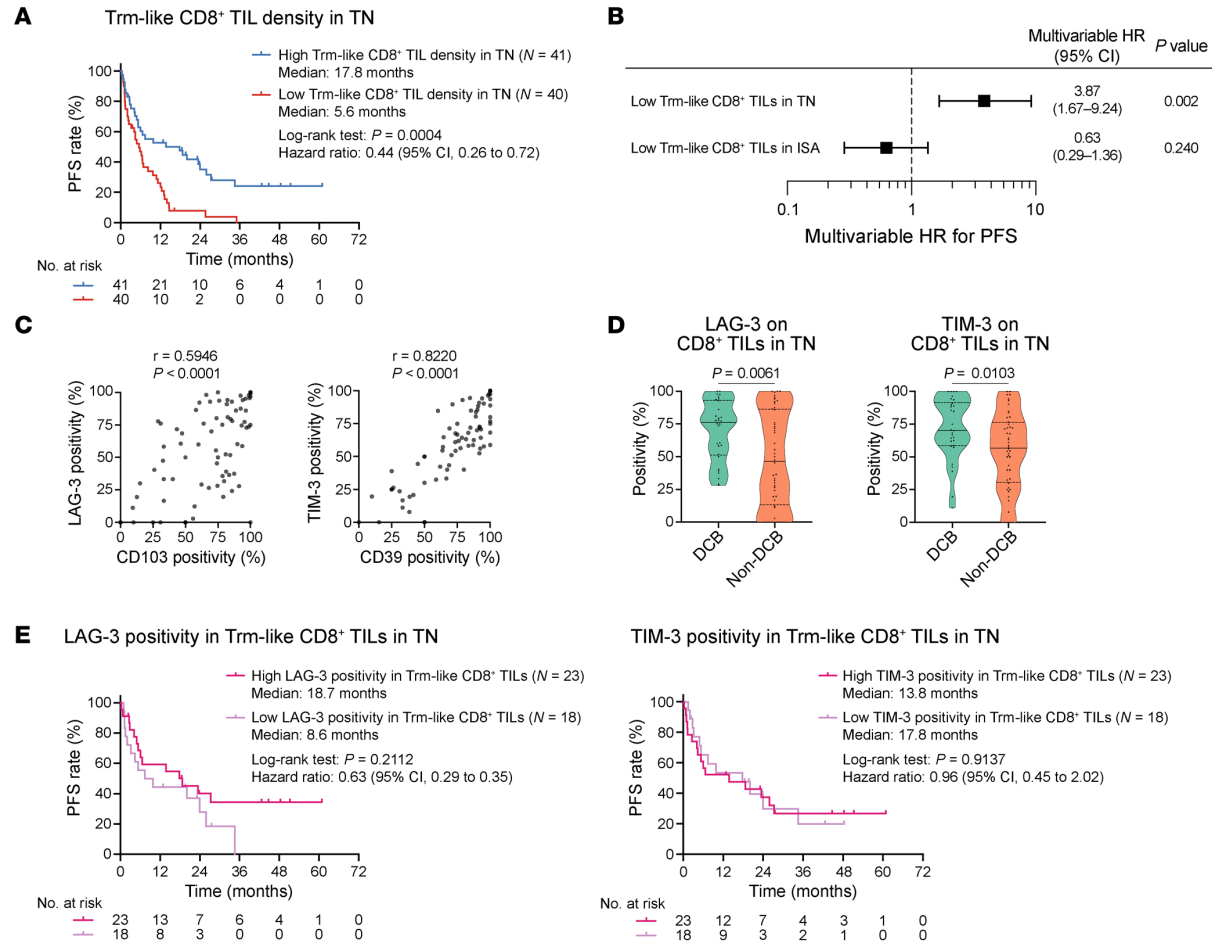
Association of CD8^+^ T cell features within TN with ICI efficacy. (**A**) KM curves for PFS of ICI treatment stratified by Trm-like (CD39^+^CD103^+^) CD8^+^ TIL density in TN. (**B**) Forest plot showing HRs for PFS estimated by multivariable Cox proportional hazards regression models evaluating the association between ICI efficacy and the indicated biomarkers. Other covariates not shown here are provided in the supplemental material. (**C**) Correlation between exhaustion marker positivity in CD8^+^ TILs in TN (LAG-3 vs. CD103 [left]; TIM-3 vs. CD39 [right]) (*N* = 80). One case was excluded because CD8^+^ T cells were absent, precluding positivity calculation. Pairwise correlations were calculated using Spearman’s correlation analysis. (**D**) LAG-3 (left) and TIM-3 (right) positivity in CD8^+^ TILs in TN compared between the DCB (*N* = 30) and non-DCB (*N* = 48) groups. Two cases were excluded because DCB/non-DCB status could not be determined due to early censoring (<1 year) of PFS data. Another case in the non-DCB group was excluded because CD8^+^ T cells were absent in the tumor, precluding positivity calculation. (**E**) KM curves for PFS stratified by LAG-3 or TIM-3 positivity in Trm-like CD8^+^ TILs in TN. Data are presented in violin plots in **D**, with each dot representing 1 patient; *P* values were determined by Mann-Whitney *U* test. *P* values for the survival analyses were determined by log-rank test. Vertical bars on KM curves indicate censoring.

We further investigated specific phenotypes of CD8^+^ TILs associated with durable tumor regression after PD-1/PD-L1 inhibition. We previously reported Trm-like CD8^+^ TILs characterized by a double positivity for both CD39 and CD103 as ICI-responsive T cells from a small cohort of patients with mNSCLC treated with PD-1/PD-L1 inhibitors (32). Therefore, we focused on this feature in the current larger NSCLC cohort. As expected, long-term disease control and short-term tumor response were more frequently observed in tumors with high Trm-like CD8^+^ TIL density (Figure 4A and Supplemental Figure 5A).

To further clarify the importance of Trm-like CD8^+^ TILs in TN for ICI efficacy, additional analyses were performed. Not surprisingly, cell densities were entirely concordant between overall CD8^+^ TILs and Trm-like CD8^+^ TILs in TN (Supplemental Figure 5B). However, a multivariable analysis revealed that Trm-like CD8^+^ TIL density in the TN is a more important ICI efficacy marker independent of overall CD8^+^ TILs (Supplemental Figure 5C). As expected, Trm-like CD8^+^ TILs also existed in ISA (Supplemental Figure 5D), but no association was found between ICI efficacy and Trm-like CD8^+^ TIL density in the ISA (Figure 4B). These findings emphasize the importance of Trm-like CD8^+^ TILs in TN.

To address unavoidable heterogeneities regarding clinical and sample backgrounds such as treatment regimens (ICI monotherapy or ICI/chemotherapy combination) or sample collection methods (surgical biopsy or nonsurgical small biopsy), we performed additional subgroup analyses to further validate our findings. The association of ICI efficacy with Trm-like CD8^+^ TIL density was consistent regardless of treatment regimens (Supplemental Figure 6A). Tissue segmentation was feasible even for such small biopsy samples (Supplemental Figure 6B). Indeed, sample collection methods did not impact the association between ICI efficacy and Trm-like CD8^+^ TIL density (Supplemental Figure 6C). These findings support the proposition that the presence of Trm-like CD8^+^ TILs in the TN is universally important for robust ICI efficacy.

However, we noted that a subset of patients still experienced early disease progression despite having a high Trm-like CD8^+^ TIL density, as demonstrated in Figure 4A. Since persistent T cell receptor (TCR) exposure to cognate antigens leads to dysfunctional hyperexhaustion of the tumor-reactive CD8^+^ TILs (terminal exhaustion) as well as CD39 and CD103 expression (19), we hypothesized that the poor response to ICI treatment observed in a substantial number of patients, even with Trm-like CD8^+^ TILs in close contact to cancer cells, was attributable to the terminal exhaustion of these T cells, caused by repetitive or excessive TCR stimulation, rendering them less effectively reinvigorated by PD-1/PD-L1 inhibition. Indeed, Trm-like features were generally correlated with T cell exhaustion markers such as PD-1, lymphocyte activation gene 3 (LAG-3), and T cell immunoglobulin and mucin domain 3 (TIM-3) (Figure 4C and Supplemental Figure 7A). However, these exhaustion markers — considered to inhibit T cell functions (19) — were not suggestive of poor tumor control after PD-1/PD-L1 inhibitor therapy. Instead, they were associated with a long-term response, particularly for LAG-3 and TIM-3, implying that these molecules are still indirect evidence of T cell capacity to kill cancer cells, consistent with their induction through antigen recognition (19) (Figure 4D and Supplemental Figure 7B). Meanwhile, the correlation maps between Trm-like features (CD39/CD103) and other exhaustion markers (PD-1/LAG-3/TIM-3/T cell immunoreceptor with Ig and ITIM domains [TIGIT]) shown in Figure 4C and Supplemental Figure 7A also demonstrated heterogeneous expression of the exhaustion markers in Trm-like CD8^+^ TILs, particularly for PD-1 and TIGIT. This prompted us to investigate the association of these exhaustion markers with PD-1/PD-L1 inhibitor efficacy in the context of high Trm-like CD8^+^ TIL density. Even in this analysis, higher expression of these exhaustion markers in Trm-like CD8^+^ TILs did not clearly lead to poor treatment efficacy (Figure 4E and Supplemental Figure 7C).

**Figure 5:**
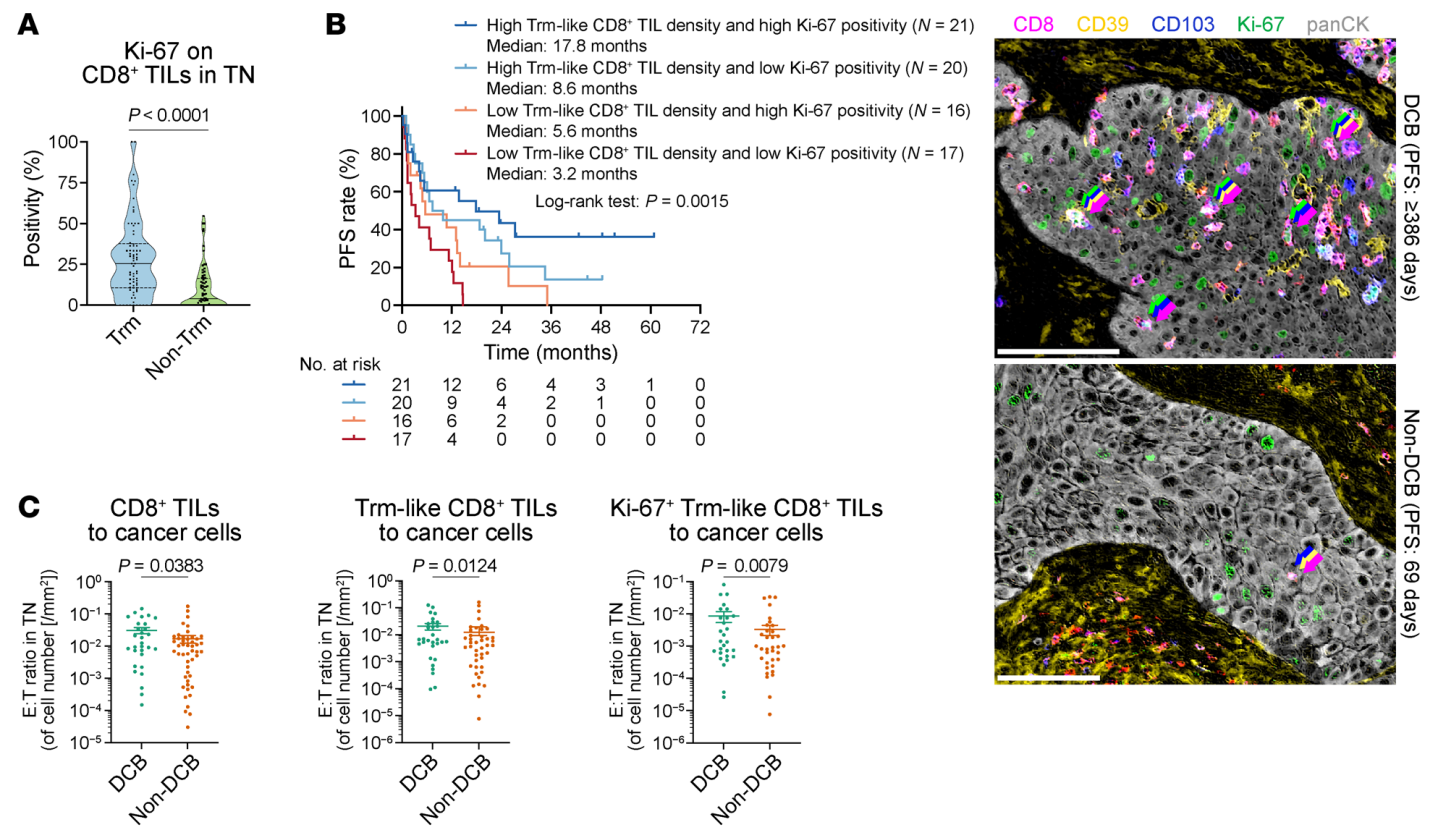
Association of Ki-67 expression in Trm-like CD8^+^ T cells with ICI efficacy. (**A**) Comparison of Ki-67 positivity between Trm-like (*N* = 74) and non–Trm-like (*N* = 76) CD8^+^ TILs in TN. Seven Trm-like and five non–Trm-like cases were excluded because the corresponding T cell populations were absent, precluding positivity calculation. (**B**) KM curves for PFS stratified by intratumoral Trm-like CD8^+^ TIL density and Ki-67 expression (left). Representative multicolor images (right) show CD8 (red), CD39 (yellow), CD103 (blue), Ki-67 (green), and panCK (gray) in a long-term ICI responder (top) and a nonresponder (bottom). Seven cases were excluded because Trm-like CD8^+^ TILs were absent, precluding positivity calculation. Arrows indicate overlaid color markers. Scale bars: 100 μm. (**C**) Cell count ratios of CD8^+^, Trm-like CD8^+^, and Ki-67^+^ Trm-like CD8^+^ TILs to cancer cells in TN compared between the DCB (*N* = 30) and non-DCB (*N* = 49) groups. Two cases were not included because DCB/non-DCB status could not be determined due to early censoring (<1 year) of PFS data. Data are presented in violin plots in **A**. Data are presented as mean ± SEM in dot plots in **C**. Each dot represents 1 patient, and *P* values were determined by Mann-Whitney *U* test. *P* values for the survival analyses were determined by log-rank test. Vertical bars on KM curves indicate censoring.

Next, we conducted detailed phenotyping of CD8^+^ TILs within the TN, using other markers in our mIHC panels, to identify more functional subsets that retained responsiveness to ICI. T-box expressed in T cells (T-bet), which is a transcription factor induced by TCR activation, did not distinguish functional Trm-like CD8^+^ TILs from their nonfunctional counterparts (Supplemental Figure 7D). Granzyme B expression, which was expected to correlate directly with effective cancer cell killing by Trm-like CD8^+^ TILs, did not show an association with improved ICI efficacy (Supplemental Figure 7D). These findings suggest that the proteins examined are not capable of reflecting ICI-responsive residual CD8^+^ TIL functionality in TME. Further, the expression levels of these markers did not differ between Trm-like CD8^+^ TILs and non–Trm-like CD8^+^ TILs (Supplemental Figure 7E). Interestingly, Ki-67 expression, which is indicative of a substantial proliferation of CD8^+^ T cells likely due to persistent TCR pathway activation, was markedly higher in Trm-like CD8^+^ TILs than in non–Trm-like CD8^+^ TILs (Figure 5A). Given this observation, we expected Ki-67 as a biological marker to distinguish exhausted yet ICI-responsive CD8^+^ TILs from terminally exhausted nonresponsive CD8^+^ TILs among tumor-reactive Trm-like CD8^+^ TILs. Indeed, Ki-67 positivity was associated with better PFS in relation to Trm-like features (Figure 5B). As higher E/T ratios are crucial for enhanced tumoricidal effect by CD8^+^ TILs in immuno-oncology experimental studies, cell count ratios of CD8^+^ TILs to cancer cells were compared between DCB and non-DCB, confirming the aforementioned findings that superior TIL features are better predictors of DCB (Figure 5C).

Collectively, Trm-like features, such as CD39 and CD103 positivity, might reflect an exhausted status of T cells due to a recent TCR activation, thus indicating a capability of CD8^+^ TILs to recognize cancer cells. Inhibitory exhaustion markers such as LAG-3, TIM-3, or TIGIT might also represent recent TCR activation and were not readily capable of identifying PD-1/PD-L1 inhibitor–resistant terminally exhausted T cells in clinical tumors. However, such ICI-resistant terminal exhaustion could instead be identified by lack of actual viability markers such as Ki-67 in recently activated CD8^+^ TILs.

### Immunosuppressive myeloid cells and CAFs as unfavorable partners for tumor-reactive CD8^+^ TILs.

Next, we evaluated the significance of nonlymphocytic immune cell subsets in TME on ICI efficacy in mNSCLC, such as TAMs, CAFs, and MDSCs.

**Figure 6:**
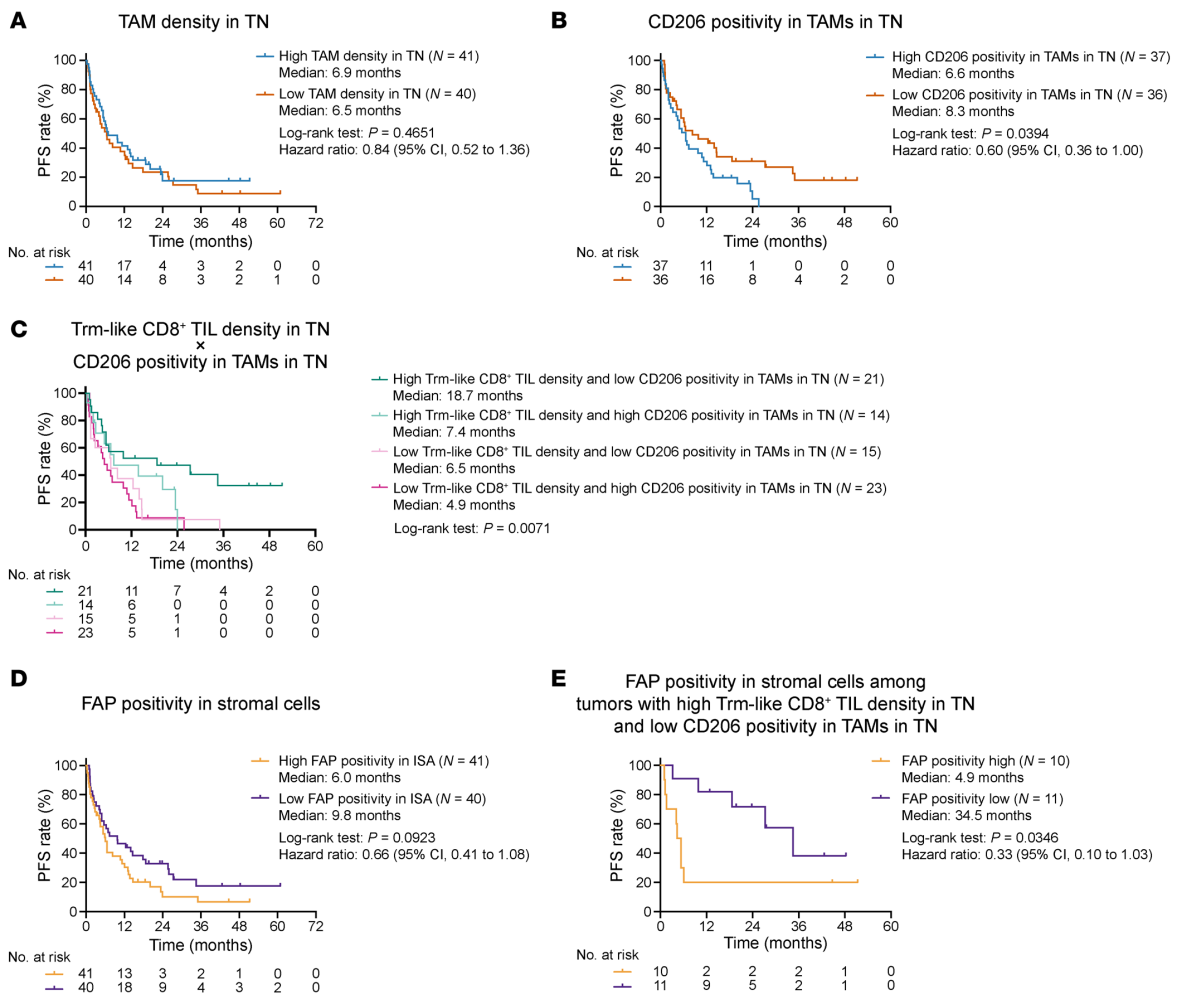
Association of TAMs and CAFs with ICI efficacy. (**A**) KM curves for PFS of ICI treatment stratified by TAM density in TN. (**B**) KM curves for PFS of ICI treatment stratified by CD206 positivity in TAMs. (**C**) KM curves for PFS of ICI treatment stratified by Trm-like CD8^+^ TIL density in TN and CD206 positivity in TAMs. (**D**) KM curves for PFS of ICI treatment stratified by FAP positivity in panCK^−^CD45^−^ cells in the ISA. (**E**) KM curves for PFS of ICI treatment stratified by FAP positivity in the stromal cells among tumors with high Trm-like CD8^+^ TIL density and low CD206 positivity in TAMs in TN. Eight cases were excluded in **B** and **C** because CD68^+^ TAMs were absent in the tumors; therefore, denominators could not be provided for positivity calculation. *P* values for the survival analyses were determined by log-rank test. Vertical bars on KM curves indicate censoring.

Pan-TAM, characterized by CD68 positivity in nonlymphocytes, did not show an association with ICI efficacy (Figure 6A). CD163^+^ M2-TAM was not apparently associated with poor ICI efficacy (Supplemental Figure 8A). However, CD206^+^ M2-TAM was linked with worse survival outcomes, consistent with its immunosuppressive and tumorigenic role in TME (43) (Figure 6B). Expression of CD163 on TAM did not additionally impact PFS, suggesting CD206 as a better marker for M2-TAM (Supplemental Figure 8B). The presence of CD206^+^ M2-TAM rendered Trm-like CD8^+^ TILs less responsive to ICI treatment, even in close contact with cancer cells (Figure 6C).

CAFs, defined by fibroblast activation protein (FAP) positivity in the ISA, exhibited a slight association with disease progression during ICI treatment, consistent with its suggested involvement in immunosuppression and cancer cell aggressiveness in preclinical studies (44) (Figure 6D). Lack of association of CAF density with both Trm-like CD8^+^ TIL density and CD206^+^ M2-TAM density in TN suggested a direct immunosuppressive effect of CAF, unrelated to the prevention of tumor-reactive CD8^+^ T cell infiltration or contribution of M2-TAM accumulation into TN (Supplemental Figure 8C). Therefore, we further interrogated the association of CAF with ICI efficacy in patients with favorable pretreatment TME profiles such as high Trm-like CD8^+^ TIL density and low CD206^+^ M2-TAM density. Of note, FAP^+^ CAF clearly distinguished long-term responders from poor responders in this particular subset (Figure 6E).

MDSCs have been considered another contributor to the immunosuppressive TME, according to previous studies; thus, this cell subset was expected to be a negative prognostic factor for ICI efficacy (45). However, the MDSC density was not different between responders and nonresponders, at least in our cohort (Supplemental Figure 8D). Intratumoral professional APCs, such as M1-TAMs and DCs, were also visualized and quantified. In accordance with a recent report (41), DCs were preferentially coexistent with Trm-like CD8^+^ TILs in TN in our study (Supplemental Figure 8E). However, these cell lineages were not associated with ICI response (Supplemental Figure 8F).

**Figure 7:**
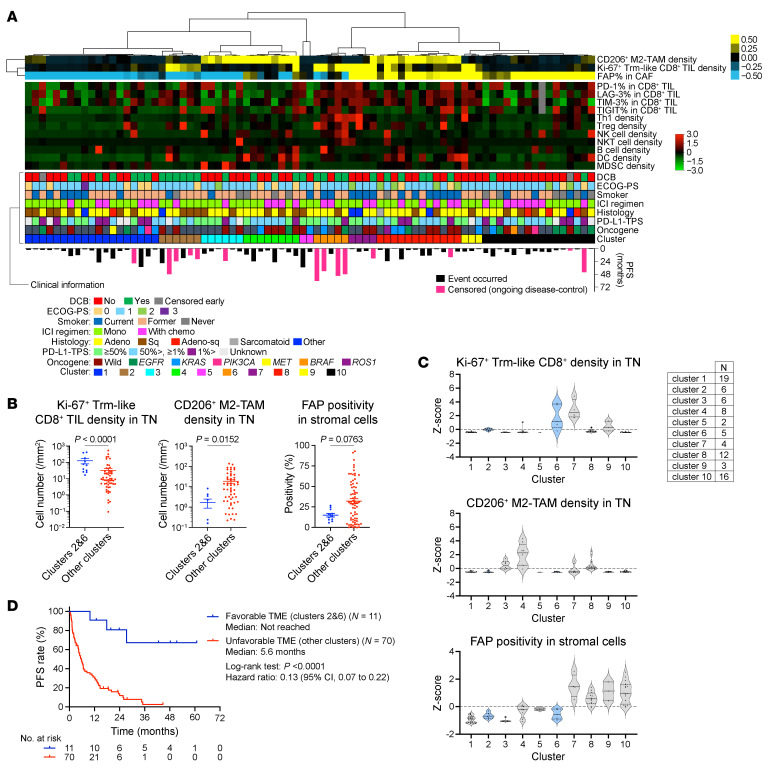
Association of intratumoral immune–stromal contexture with ICI efficacy. (**A**) Unsupervised hierarchical clustering results of ICI cohort according to Ki-67^+^ Trm-like CD8^+^ TIL density, CD206^+^ TAM density, and FAP positivity in stromal cells are visualized as a heatmap. Each tile represents the *z* score for key TME features (yellow, highest; black, median; blue, lowest). Additional mIHC-derived TME features are shown in a second heatmap (red, highest; black, median; green, lowest). Clinicopathologic characteristics and PFS for each patient are shown below the heatmaps. (**B**) Ki-67^+^ Trm-like CD8^+^ TIL density, CD206^+^ M2-TAM density, and FAP positivity in stromal cells compared between patients in clusters 2 and 6 (*N* = 11) and those in other clusters (*N* = 70). (**C**) The *z* scores for Ki-67^+^ Trm-like CD8^+^ TIL density (top), CD206^+^ M2-TAM density (middle), and FAP positivity in stromal cells (bottom) across all 10 clusters. (**D**) KM curves for PFS of ICI treatment comparing favorable (clusters 2 and 6) and unfavorable (other clusters) TME. Data are presented in violin plots in **C**. Each dot represents 1 patient in **B** and **C**. Data are presented as mean ± SEM in dot plots. *P* values for dot plots and survival analyses were determined by Mann-Whitney *U* test and log-rank test, respectively. Vertical bars on KM curves indicate censoring. Mono, monotherapy; chemo, chemotherapy; Adeno, adenocarcinoma; Sq, squamous cell carcinoma; Adeno-sq, adenosquamous.

TME features are summarized alongside the clinicopathologic/genomic features in Figure 7A. The ICI cohort is subcategorized by unsupervised hierarchical clustering based on key cell subsets, including functional (Ki-67^+^) tumor-reactive (CD39^+^CD103^+^) CD8^+^ TILs, immunosuppressive (CD206^+^) M2-TAMs, and immunosuppressive (FAP^+^) CAFs, thereby highlighting patients in clusters 2 and 6 as having more favorable TMEs (fTMEs), such as substantial functional tumor-reactive CD8^+^ TILs without immunosuppressive M2-TAMs or CAFs (Figure 7, B and C). These mathematically extracted subcohorts also exhibited better ICI efficacy, thus confirming our hypothesis (Figure 7D). Presumably, antitumor effect of tumor-reactive CD8^+^ TILs with cellular contact with cancer cells might be abrogated by the juxtaposed immunosuppressive cells, such as CD206^+^ M2-TAMs or FAP^+^ CAFs. To examine whether this association varied by cancer cell biology, we performed subgroup analyses by histology (squamous or nonsquamous) and oncogenic driver status (*EGFR*-mutations, non-*EGFR/ALK* driver oncogenes, or no apparent driver oncogenes). Although the number of cases in each subgroup was limited, the predictive feature of this multicellular TME profiling for ICI efficacy appeared consistent across histologic and genomic features (Supplemental Figure 9).

We next sought an independent external cohort to test the validity of our findings. Spatial single-cell proteotyping of a substantial number of patients with mNSCLC, at this depth and with corresponding ICI treatment efficacy data, is not publicly available to date. Therefore, we utilized a bulk RNA-seq database from the SU2CLC-MGH cohort (46). *CD8B*, *MRC1*, and *FAP* were used, as they are relatively dominant in each cell type (CD8^+^ TILs, CD206^+^ M2-TAMs, and CAFs, respectively). Each gene was only partially associated with ICI efficacy (Supplemental Figure 10A). This was anticipated given that this bulk transcriptome data lack spatial, protein-level, and single-cell-level information as opposed to our platform. In particular, the subtle nuance characterized by Ki-67^+^ Trm-like CD8^+^ TILs in TN cannot be delineated from this approach. However, a composite profile (*CD8B*^hi^*MRC1*^lo^*FAP*^lo^) mimicking the fTME showed a better tendency toward ICI efficacy (Supplemental Figure 10B). This also illustrates that high-depth but labor-intensive spatial single-cell proteotyping, underpinned by prior immuno-oncology knowledge, can guide reliable models using low-resolution, indiscriminate, yet practical platforms, such as bulk transcriptome assays.

To explore underlying potential mechanisms causing the difference between the fTME and unfavorable TME (ufTME), we performed nCounter IO360 transcriptome analysis. Twenty-two pre–ICI treatment tumors that had sufficient archival tissue amenable to good quality mRNA extraction were evaluated. Note that only 4 samples were available from the fTME for this analysis. As expected, genes related to activated T cells were upregulated in the fTME, whereas those related to myeloid cells and stromal cells were upregulated in the ufTME (Supplemental Figure 11). Several potential therapeutic targets for the ufTME were suggested, including *AREG*, *DPP4*, *ANGPTL4*, and *VEGFA*. Despite the small sample size, this might provide a clue for future research.

**Figure 8:**
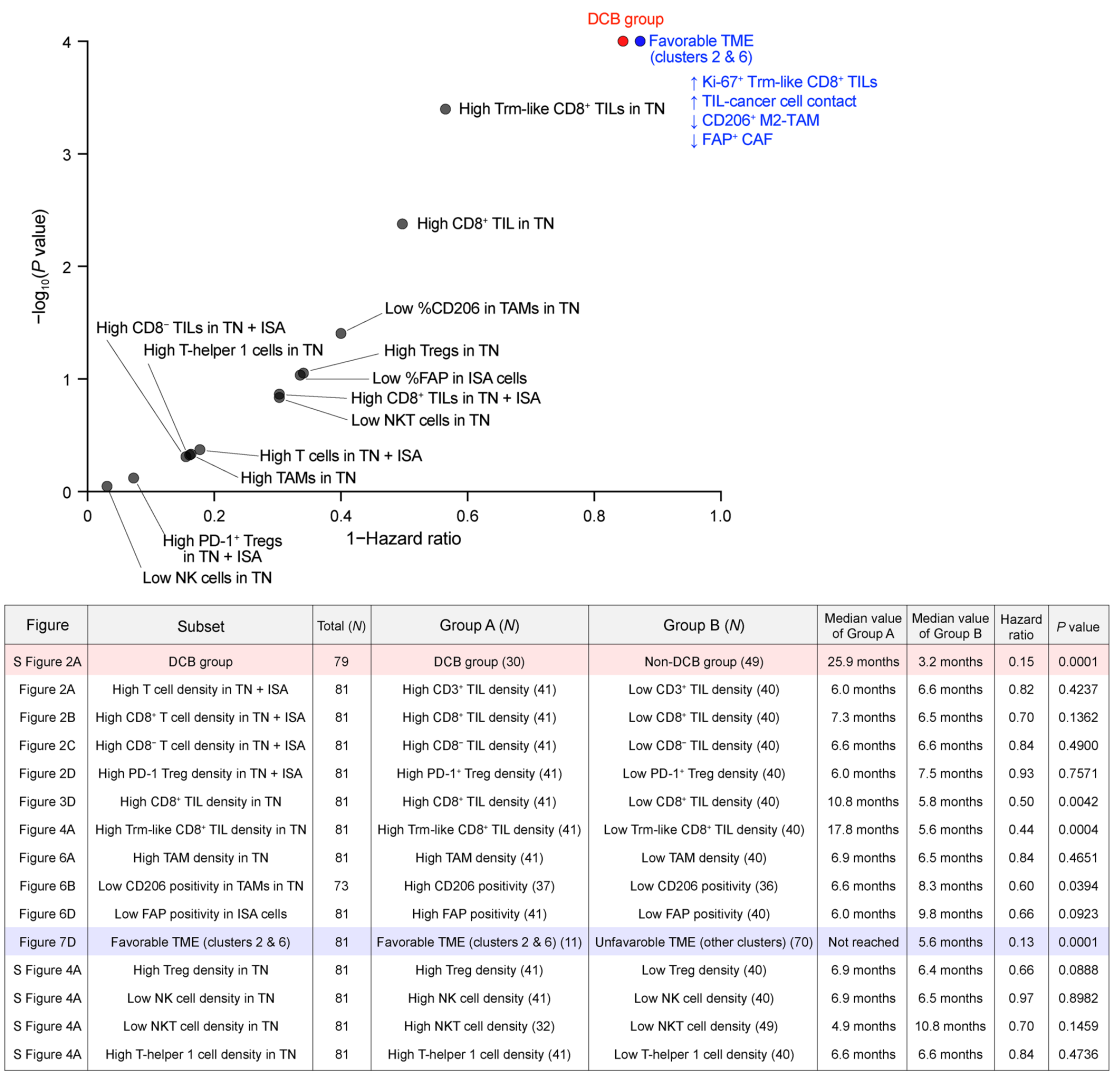
Predictive values of TME profiles for ICI efficacy. The predictive values of TME profiles for ICI efficacy are plotted based on their HRs and *P* values from all PFS analyses performed based on mIHC findings in this study (top). The red dot indicates the predictive value of DCB from Supplemental Figure 2A as a positive control. The blue dot represents the fTME (clusters 2 and 6) from Figure 7A. Note that the mIHC-trained pretreatment prospective profile (blue dot) is equivalent to or even better than the retrospective endpoint-trained category (red dot) for predicting long-term tumor control with ICI. Relevant details, including the number of samples analyzed, are summarized in the table (bottom).

The predictive values of TME profiles examined in our mIHC study for PFS during ICI treatment are summarized in Figure 8. This underscored the importance of CD8^+^ TILs instead of other immune cells, the residual antitumor reactivity of the CD8^+^ TILs, a physical contact of those cells with cancer cells, absence of M2-TAMs and immunosuppressive CAFs, and ultimately co-occurrence of all of these profiles.

### Immunosuppressive TME characterized by M2-TAM infiltration and aberrant angiogenesis/TGF-β signaling in EGFR/ALK-oncogene^+^ NSCLC in relation to CD73 expression.

**Figure 9:**
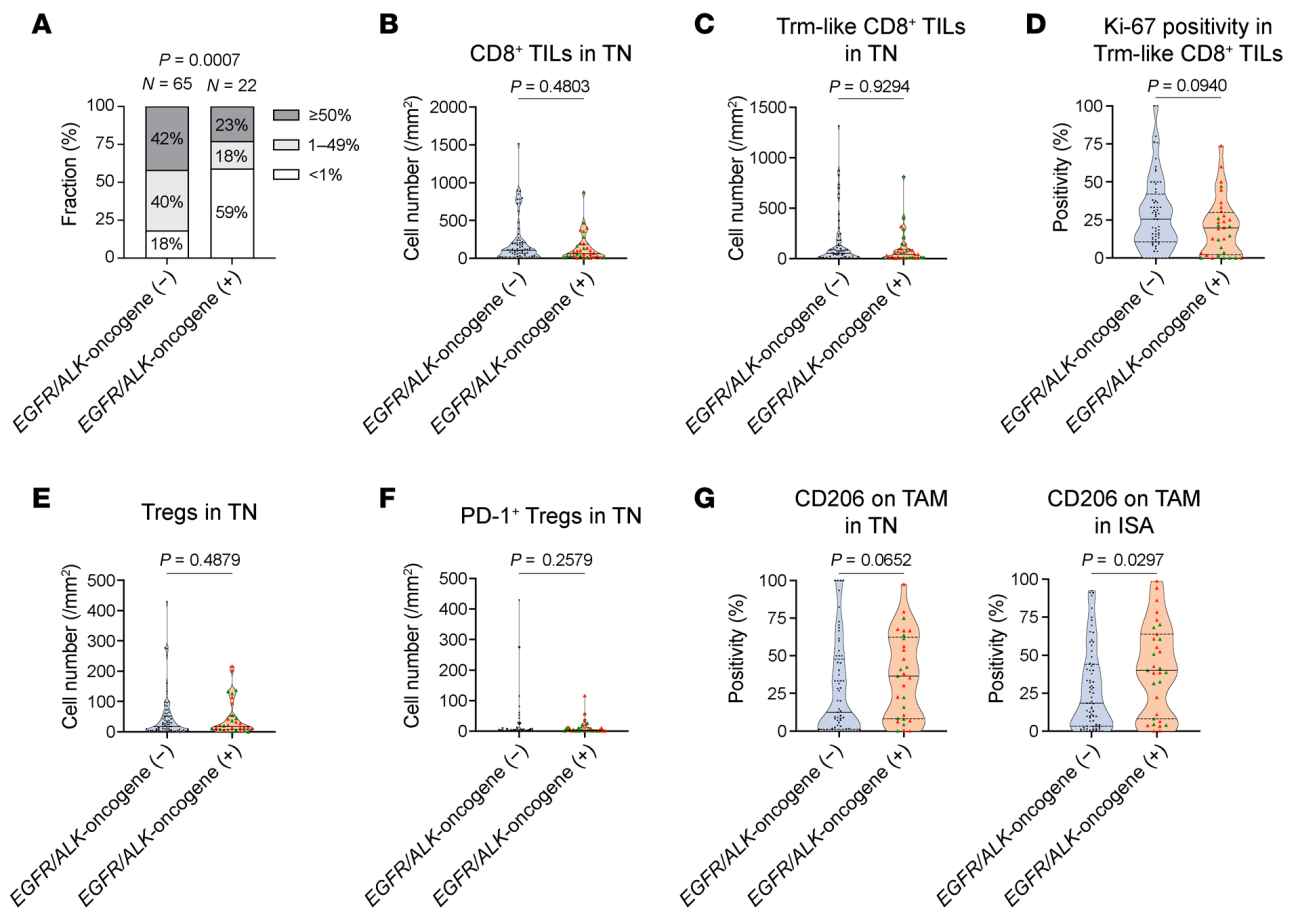
TME features of *EGFR*/*ALK*-oncogene^+^ NSCLC. (**A**) PD-L1 TPS compared between *EGFR/ALK*-oncogene^–^ tumors (*N* = 65) and *EGFR/ALK*-oncogene^+^ tumors (*N* = 22). Six *EGFR/ALK*-oncogene^–^ cases and 10 *EGFR/ALK*-oncogene^+^ cases were excluded due to unavailable PD-L1 TPS data. (**B** and **C**) CD8^+^ T cell densities (**B**) and Trm-like CD8^+^ T cell densities (**C**) in TN compared between *EGFR/ALK*-oncogene^–^ tumors (*N* = 71) and *EGFR/ALK*-oncogene^+^ tumors (*N* = 32). (**D**) Ki-67 positivity in Trm-like CD8^+^ T cells in TN compared between *EGFR/ALK*-oncogene^–^ tumors (N = 64) and *EGFR/ALK*-oncogene^+^ tumors (*N* = 32). Seven *EGFR/ALK*-oncogene^–^ cases were excluded because Trm-like CD8^+^ TILs were absent, precluding positivity calculation. (**E** and **F**) Treg densities (**E**) and PD-1^+^ Treg densities (**F**) in TN compared between *EGFR/ALK*-oncogene^–^ tumors (*N* = 71) and *EGFR/ALK*-oncogene^+^ tumors (*N* = 32). (**G**) CD206^+^ TAM densities in TN and ISA compared between *EGFR/ALK*-oncogene^–^ tumors (*N* = 65 for TN; *N* = 71 for ISA) and *EGFR/ALK*-oncogene^+^ tumors (*N* = 29 for TN; *N* = 31 for ISA). Cases were excluded if CD68^+^ TAMs were absent, precluding positivity calculation (6 cases in TN of *EGFR/ALK*-oncogene^–^ tumors, 3 cases in TN of *EGFR/ALK*-oncogene^+^ tumors, and 1 case in ISA of *EGFR/ALK*-oncogene^+^ tumors). Data are presented in violin plots in **B**–**G**, with each dot representing 1 patient; tumors with *EGFR*-mutations are colored red, and those with *ALK*-fusions are colored green. *P* values were determined by Fisher’s exact test in **A** and Mann-Whitney *U* test in **B**–**G**.

Next, we dissected TME profiles of *EGFR/ALK*-oncogene^+^ NSCLC, which are confirmed by our cohort to be primarily resistant to ICI therapy (Supplemental Figure 2F), to further identify unique features associated with such poor ICI response in this distinctive NSCLC subset. Patient characteristics for this subset are provided in Supplemental Table 4. As expected, the majority of cases with driver oncogene^+^ NSCLC were individuals who had never smoked and exhibited nonsquamous histology, thus representing classical *EGFR/ALK*-oncogene^+^ NSCLC (Supplemental Figure 12A). Indeed, the clinically available PD-L1 TPS was lower in *EGFR/ALK*-oncogene^+^ NSCLC than in *EGFR/ALK*-wt NSCLC, aligning with previous findings (35, 47) (Figure 9A). However, CD8^+^ TIL density did not show a considerable difference between *EGFR/ALK*-oncogene^+^ NSCLC and *EGFR/ALK*-wt NSCLC, in both TN and ISA (Figure 9B and Supplemental Figure 12B). Similarly, Trm-like CD8^+^ TIL density in TN was comparable between these 2 NSCLC subtypes (Figure 9C). These findings suggest that the tolerance of *EGFR/ALK*-oncogene^+^ NSCLC to ICI therapy is not solely due to a fundamental deficiency in neoantigen-reactive T cells. However, functional Trm-like CD8^+^ TILs, indicated by Ki-67 expression, were diminished in these tumor types, suggesting potential mechanisms hindering CD8^+^ TIL function (Figure 9D).

Intratumoral Treg density did not differ based on *EGFR/ALK*-oncogene status in both TN and ISA (Figure 9E and Supplemental Figure 12C), even when considering PD-1 expression on Tregs (Figure 9F and Supplemental Figure 12D), implying a limited role for Tregs in ICI resistance in this specific tumor within our mNSCLC cohort. Next, we investigated the involvement of other immunosuppressive cells, such as M2-TAMs and MDSCs. Intriguingly, CD206^+^ M2-TAMs were more infiltrated into both TN and ISA in *EGFR/ALK*-oncogene^+^ NSCLC than in *EGFR*/*ALK*-oncogene^–^ NSCLC for these gene alterations (Figure 9G), although MDSC density was not different (Supplemental Figure 12E).

**Figure 10:**
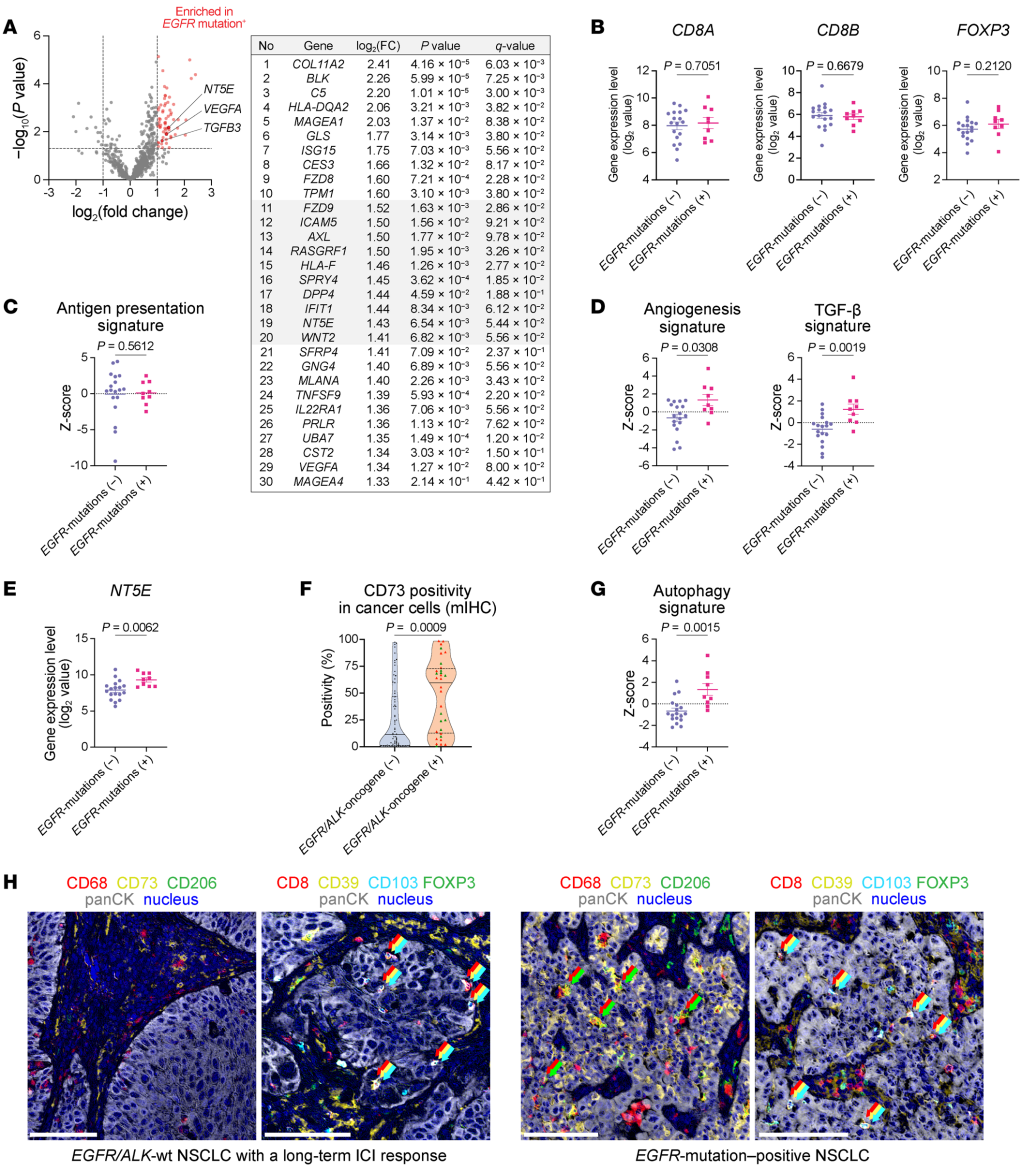
*EGFR*-mutation–associated gene expression and spatial immune features. (**A**) Volcano plot showing −log_10_(*P* value) and log_2_(fold change [FC]) of gene expression levels (*EGFR*-mutant [*N* = 9] vs. *EGFR*-wt [*N* = 18]) for 726 genes (left). The top 30 upregulated genes in *EGFR*-mutation^+^ tumors are listed with *q* values (right), ranked by the log_2_(FC). (**B**) Gene expression levels of *CD8A*, *CD8B*, and *FOXP3* compared between *EGFR*-mutation^–^ NSCLC (*N* = 18) and *EGFR*-mutation^+^ NSCLC (*N* = 9). (**C** and **D**) Gene expression signature scores (antigen presentation, angiogenesis, and TGF-β signaling) compared between *EGFR*-mutation^–^ NSCLC (*N* = 18) and *EGFR*-mutation^+^ NSCLC (*N* = 9). (**E**) Gene expression levels of *NT5E* compared between *EGFR*-mutation^–^ NSCLC (*N* = 18) and *EGFR*-mutation^+^ NSCLC (*N* = 9). (**F**) CD73 protein positivity in cancer cells compared between *EGFR/ALK*-oncogene^–^ tumors (*N* = 71) and *EGFR/ALK*-oncogene^+^ tumors (*N* = 32). (**G**) Autophagy signature scores compared between *EGFR*-mutation^–^ NSCLC (*N* = 18) and *EGFR*-mutation^+^ NSCLC (*N* = 9). (**H**) Representative mIHC images of M2-TAM (CD68^+^CD206^+^), CD73-expressing cancer cells, Trm-like (CD39^+^CD103^+^) CD8^+^ T cells, and Tregs (FOXP3^+^) are shown for *EGFR*-wt NSCLC (left) and *EGFR*-oncogene^+^ NSCLC (right). The *EGFR*-oncogene^+^ NSCLC presented a higher intratumoral M2-TAM infiltration and CD73 expression on cancer cells compared with the WT tumors. Substantial Trm-like CD8^+^ T cell infiltration was verified in the *EGFR*-oncogene^+^ tumor as equivalent to the WT tumor. Treg densities were comparable between the 2 distinctive tumor types. Arrows represent overlaid color markers. Scale bars: 100 μm. Data are presented in violin plots in **F**, with each dot representing 1 patient; tumors with *EGFR*-mutations are colored red, and those with *ALK*-fusions are colored green. Data are presented as mean ± SEM in dot plots in **B**–**E** and **G**. *P* values were determined by unpaired 2-tailed *t* tests in **A** and Mann-Whitney *U* test in **B**–**G**.

To further dissect TME profiles based on these ICI-resistant driver oncogenes, we compared transcriptomic profiles. In addition to the 22 tumors analyzed in Supplemental Figure 11, 5 additional non–ICI-treated *EGFR*-mutant tumors were added. Signature scores for MAPK and NF-κB signaling pathways were expectedly increased in *EGFR*-mutant NSCLC compared with *EGFR*-wt NSCLC, in agreement with *EGFR* downstream pathway activation caused by activating *EGFR*-mutations (48) (Supplemental Figure 12F). Differentially expressed genes are shown in Figure 10A and Supplemental Figure 12G. Consistent with the mIHC data, *CD8A*, *CD8B*, and *FOXP3* genes were similarly expressed between tumor samples from *EGFR*-mutant NSCLC and *EGFR*-wt NSCLC (Figure 10B). GSEA showed that antigen presentation machinery was not suppressed in our *EGFR*-mutant NSCLC (Figure 10C), thus supporting the notion that *EGFR*-mutant NSCLC is potentially capable of being recognized by CD8^+^ TILs, as also evidenced by the existence of Trm-like CD8^+^ TILs in TN in Figure 9C. In contrast, signatures for angiogenesis and TGF-β signaling were upregulated in *EGFR*-mutant NSCLC (Figure 10D), consistent with the higher M2-TAM infiltration in this tumor type, as shown in Figure 9G. Among the upregulated genes listed in Figure 10A, we focused on the *NT5E* gene (Figure 10E), whose protein CD73 was visualized using mIHC. Indeed, CD73 protein from cancer cells was more frequently expressed in *EGFR/ALK*-oncogene^+^ NSCLC than in *EGFR/ALK*-wt NSCLC (Figure 10F), which might be at least partially responsible for the M2-TAM/angiogenesis/TGF-β circuit due to adenosine production via the ATP/CD73 pathway (49). Of note, the *EGFR*-mutant NSCLC exhibited an increased autophagy signature, consistent with *EGFR*-mediated MAPK pathway activation (50), which may reflect enhanced ATP availability (51–53) and thereby more effective activation of the CD73 pathway in this NSCLC subtype (Figure 10G).

To validate our transcriptome findings, publicly available RNA-seq data were obtained from the lung adenocarcinoma and lung squamous cell carcinoma cohorts in The Cancer Genome Atlas (TCGA) (54), comprising 64 *EGFR/ALK*-oncogene^+^ tumors including 59 *EGFR*-mutant cases and 927 *EGFR/ALK*-wt tumors (Supplemental Figure 13A). As expected, differential gene expression analysis confirmed that *NT5E* expression level was apparently higher in *EGFR/ALK*-oncogene^+^ tumors than in *EGFR/ALK*-wt tumors, as this gene was ranked as a top 1.9 percentile upregulated gene (Supplemental Figure 13B). Gene expression levels regarding angiogenesis and TGF-β pathway, such as those for VEGF ligands, TGF-β ligands and TGF-β receptors, were also compared between these tumor types, indicating higher expression of *VEGFB*, *TGFB2*, and *TGFBR2* in *EGFR/ALK*-oncogene^+^ tumors (Supplemental Figure 13C). Of note, *MRC1* (CD206 gene) was also apparently upregulated in *EGFR/ALK*-oncogene^+^ tumors (as a top 3.1 percentile upregulated gene) (Supplemental Figure 13D). These data collectively corroborate findings from our original cohort.

Overall, a general tolerance of *EGFR/ALK*-oncogene^+^ NSCLC to PD-1/PD-L1 inhibitor therapy is likely to be due to its unique immunosuppressive TME, as characterized by aberrant angiogenesis and TGF-β upregulation as well as M2-TAM accumulation, rather than intratumoral infiltration of Tregs or scarcity of tumor-reactive CD8^+^ T cells, which might be attributable to CD73/adenosine pathway activation predominantly caused by *EGFR*-activated cancer cells (Figure 10H).

## Discussion

Recent advances in immunotherapy within the clinical oncology field has rapidly progressed cancer immunology research. Numerous preclinical studies have investigated TME to understand the immunomodulatory role of different immune cell subsets and the significance of heterogeneous features observed in CD8^+^ TILs, which are considered a primary source of antitumor immunity (15). However, translating preclinical findings to clinical scenarios requires careful interpretation, given the inherent biological differences between preclinical samples and human bodies (15). Understanding how heterogeneous immune cell components in the complex TME influence ICI efficacy in humans remains incomplete. The challenge of obtaining fresh tumor samples for a large cohort limits deep TME analysis in clinical studies. In this study, we utilized spatial single-cell profiling at the protein level for many patients to dissect the TME in a heterogeneous cellular context. Exhaustive interrogation using sophisticated computer analysis enabled us to generate multidimensional data, such as simultaneous quantification and visualization of heterogeneous cell subsets with spatial information, taking into account cellular distance consistently. We thereby revealed that functional tumor-reactive CD8^+^ TILs are exclusively responsible for ICI efficacy only when they are in close contact to cancer cells. In contrast, other lymphocytes might be bystander cells that were not clearly associated with therapeutic efficacy and resistance to the therapy in mNSCLC. We simultaneously visualized other immunosuppressive components and T cells and suggest M2-TAM and CAF as unfavorable partners for the tumor-reactive CD8^+^ TILs. To our knowledge, this is the first study performing such a comprehensive cell profiling in TME using multiplex proteotyping in a large number of patients with mNSCLC treated with ICI.

Two recent studies using AI indicated that preexisting TILs predict ICI response in mNSCLC independently from conventional biomarkers such as PD-L1 TPS and tumor mutation burden (12, 13). However, as also pointed out in their papers, this AI-based TIL count is predicated on their morphology in H&E stains and is thereby incapable of distinguishing further lymphocytic subsets. As demonstrated in this study, various types of lymphocytes, including Tregs and Th1, NK, B, and CD8^+^ T cells, exist in the intratumoral area and constitute the overall TIL population with similar morphology, of which biological diversity should be taken into consideration for ICI response. As CD8^+^ TILs mainly rely on their CXCR3 expression, which enables their infiltration into tumors by its chemoattractant ligands CXCL9/10/11, such non-CD8^+^ TILs also generally express CXCR3, thereby supposedly behaving as bystander lymphocytes (55). Indeed, we indicated that non-CD8^+^ TILs were not associated with ICI response and highlighted the importance of a specific evaluation of CD8^+^ TILs for a more precise biomarker. Meanwhile, recently, tertiary lymphoid structure, mainly consisting of B cells and CD4^+^ T cells, has also been reported to be associated with ICI response in solid tumors (56–58). Our study exclusively focused on intratumoral area, where tertiary lymphoid structure is less likely to present (59). Accordingly, future studies should also focus on the peritumoral area, particularly in perioperative settings with abundant surgical specimens available.

In agreement with our study, several previous clinical studies also indicated that preexisting CD8^+^ TILs could predict better survival outcomes with ICI treatment in mNSCLC (11, 14). However, the best spatial definition of TILs has been unclear because these studies used transcriptomic data and thus lacked spatial information or they solely investigated TILs located in TN. Our study interrogated a divergent significance of CD8^+^ TILs on the treatment efficacy between TN and ISA, further highlighting that the durable ICI response requires physical contact of the CD8^+^ TILs with cancer cells. This finding is consistent with effective direct cell–cell interaction between CD8^+^ T cells and cancer cells and is necessary for valid cancer cell killing (60). Combining more sophisticated techniques calculating precise distance between cells (61) with our exhaustive mIHC profiling technique is warranted to provide additional interpretation.

In addition to the spatial distribution information, we also evaluated functional and differentiation markers of CD8^+^ T cells, including granzyme B, T-bet, and Ki-67. Despite their established roles in cytotoxic activity and effector programming (62, 63), neither granzyme B nor T-bet showed predictive value for ICI efficacy in our dataset, whereas Ki-67 did. Our study was not capable of testing causal relationships, possibly because Ki-67 is correlated with long-lasting T cell survival given that it directly reflects upcoming proliferation of T cells, whereas other markers might be just snapshots of recent or nonspecific activation regardless of its durability. These findings suggest that sustained proliferative capacity, rather than immediate cytotoxic potential or differentiation status, is critical for effective and long-term ICI responses.

Previous preclinical studies using mouse models suggested that non–PD-1 exhaustion molecules such as LAG-3, TIM-3, and TIGIT expressed on T cells might be resistance mechanisms to ICI therapy on the rational basis of their inhibitory function against TCR downstream signals independently from the PD-1 pathway (17, 19). This preclinical evidence also raises the possibility that high expression levels of these molecules on preexisting CD8^+^ TILs might predict a consequent failure of the following PD-1/PD-L1 inhibitor therapy in a clinical context. However, the fact that these molecules are provoked by TCR activation, which is essential to tumor response associated with ICI therapy, complicates the interpretation of the association between the expression of these exhaustion markers and the treatment efficacy. Our intensive analysis for this perspective supported the notion that these exhaustion markers are merely indirect markers for PD-1/PD-L1 inhibitor–reactive T cells but are not involved in resistance to the therapy in mNSCLC. That said, given that these exhaustion markers might be further and persistently upregulated by a reinvigoration of tumor-reactive T cells following PD-1/PD-L1 blockade, further investigation of the association between the exhaustion molecules increased after ICI therapy and the consequent disease progression is warranted. To this end, dissecting the TME before and after ICI using paired tumor tissues in neoadjuvant immunotherapy settings is needed.

CD39 has been recently recognized as a T cell exhaustion marker (19). In addition, intratumoral CD103^+^CD8^+^ T cells have been characterized as tumor-reactive T cells by their tissue residency and their frequent coexpression of other exhaustion markers (64). Chow et al. performed a preliminary study including 23 patients with mNSCLC, which suggested the association between intratumoral CD39^+^CD8^+^ T cells and ICI efficacy by nonspatial analysis, such as scRNA-seq and flow cytometry (65). Yeong et al. performed another preliminary study including 35 patients with mNSCLC, which also suggested the association of intratumoral CD39^+^CD8^+^ T cells with ICI efficacy by 6-color mIHC (30). In addition, Corgnac et al. suggested the association between intratumoral CD103^+^CD8^+^ T cells and ICI efficacy in 86 patients with mNSCLC by 3-color mIHC (31). Our prior pilot work comprising 4 responders and 4 nonresponders further indicated that intratumoral CD39^+^CD103^+^CD8^+^ T cells are necessary for initial ICI response but are not sufficient for persistent ICI response (32). These observations prompted further investigation into the spatial interplay between tumor-reactive CD8^+^ TILs and other immune cell types such as non-CD8^+^ T lymphocytes as well as TAMs and CAFs, as we addressed in this study.

As revealed by our current findings, M2-TAMs and CAFs have been historically suggested as unfavorable cell subsets for ICI efficacy due to their immunosuppressive roles, which have mainly been reported by preclinical studies (15, 20, 43, 44). Recent studies implied clinical relevance of these cell types in ICI efficacy. Li et al. implicated a detrimental effect of TAMs on ICI efficacy in a small cohort involving 20 patients with stage IV NSCLC using 7-color mIHC (66). Peyraud et al. suggested a negative impact of CAFs on ICI efficacy in 77 patients with stage IV NSCLC using 6-color mIHC (58). These data warranted more comprehensive dissection of TME in the context of ICI efficacy in mNSCLC. Our 29-color mIHC study is thus notable for the simultaneous, thorough evaluation of 2 cell populations alongside tumor-reactive CD8^+^ TILs. This approach provides an in-depth interpretation of these previously debated cells. Owing to the advantages offered by single-cell spatial proteotyping, our study unveiled the nuanced yet unequivocal detrimental effect of M2-TAMs and CAFs on the clinically effective reinvigoration of preexisting cancer cell–killing CD8^+^ TILs by ICI therapy in mNSCLC. Supplemental Table 5 summarizes the comparison between our study and these relevant pilot studies.

While the direct role of CD206 in cancer biology remains unclear, it is generally recognized to label M2-TAMs that produce immunosuppressive or tumorigenic cytokines such as IL-10, TGF-β, and VEGF (43). These cytokines inhibit the cytotoxic function of antigen-reactive CD8^+^ TILs by inhibiting effective TCR pathway activation (67, 68), aligning with our findings that preexistent tumor-reactive CD8^+^ TILs were not effectively utilized by ICI because of the involvement of such M2-TAMs. CAF populations are heterogeneous cell lineages that are subcategorized into distinct phenotypes based on their divergent roles (44). We predetermined FAP^+^ CAFs as the most important subset, consistent with previous studies suggesting this subset as the most immunosuppressive phenotype (44). They also provide the same immunosuppressive cytokines such as IL-10, TGF-β, and VEGF (44, 69). The impact pattern of FAP^+^ CAFs on ICI efficacy recapitulates that of CD206^+^ M2-TAMs, highlighting the importance of these immunosuppressive cytokines as the next targets.

Of note, our small exploratory subgroup analyses indicated that predictive features of the TME profile — based on functional neoantigen-reactive T cells, M2-TAMs, and immunosuppressive CAFs — were consistently observed regardless of different cancer cell biology based on histology or major driver oncogene positivity such as *EGFR* and *KRAS*. Unfortunately, limited clinical accessibility to comprehensive genomic profiling in Japan — where the regulatory agency (Pharmaceuticals and Medical Devices Agency) requires patients to have undergone all standard-of-care treatments to qualify — prevented more detailed analyses considering tumor mutation burden and other uncommon gene alterations such as those of *KEAP1*, *STK11*, or *SMARCA4*. Of note, however, our data suggested that precise identification of functional CD8^+^ TILs having a direct contact with cancer cells, M2-TAMs, and immunosuppressive CAFs adds better predictive performance for long-term ICI efficacy even where a classical biomarker PD-L1 TPS fails to predict it. Our findings therefore propose a future direction to study immune profiling in relation to these genomic features that are becoming increasingly clinically relevant.

Our mNSCLC cohort included substantial *EGFR/ALK*-oncogene^+^ patients, enabling us to investigate the immunological features of these unique NSCLC subtypes known for their tolerance to ICI therapy. Notably, *EGFR/ALK*-oncogene^+^ patients in our cohort represented typical characteristics, including nonsquamous histology, light smoking history, low PD-L1 TPS, and substantial activation of RTK downstream pathways. This justified our analysis of these tumor types. Consequently, we revealed that their tolerance to ICI treatment was not solely due to a fundamental lack of immunogenicity but may be associated with certain immunosuppressive features such as those with M2-TAM, the CD73/adenosine pathway, and desmoplastic phenotypes involving TGF-β or aberrant angiogenesis. However, some discrepancies exist between our findings and previous research on TME profiles for *EGFR/ALK*-oncogene^+^ NSCLC. For example, while previous studies suggested that *EGFR*-mutant NSCLC was associated with a low CD8^+^ TIL density compared with *EGFR*-wt NSCLC, our findings contradict this (47, 70). As a possible explanation, tumor tissues from *EGFR*-mutant NSCLC subjected to our analyses were exclusively metastatic, whereas most previous studies mainly collected nonmetastatic early-stage NSCLC (47, 70). A previous study suggested that Tregs were highly infiltrated into *EGFR*-mutant NSCLC in comparison with *EGFR*-wt NSCLC (70). The discrepancy between that study and ours might also be due to differences in disease stages. Hence, we reemphasize the strength of our study that all samples were exclusively metastatic tumors, which would be clinically relevant to our primary clinical question. Overall, this study suggests promising future treatment targets for *EGFR*-mutant mNSCLC, such as CD73/adenosine pathway, angiogenesis, or TGF-β in the context of ICI combination therapy. Importance of antiangiogenesis in immunotherapy for this type of tumor has been implicated by *EGFR*-mutant subgroup analyses from previous clinical trials, although observed benefit was still marginal, and additional strategies are thus warranted (71). Several experimental in vitro and in vivo studies — in which CD73 was inhibited in human or mouse *EGFR*-mutant NSCLC cell lines with CD73 antibody or CRISPR/Cas9-mediated gene deletion — indicated the possibility that CD73 blockade sensitizes *EGFR*-mutant NSCLC to CD8^+^ T cell attack (72–74). Of note, CD73 inhibitors such as oleclumab are currently being developed, and very preliminary clinical trial data implicated that these investigational drugs would be potential ICI sensitizers in ICI-resistant NSCLC cases (75, 76).

A limitation of our study is that it is a single-institution retrospective study without a validation cohort, and our cohort consisted exclusively of Japanese patients. Therefore, further investigation to validate our findings in other populations is encouraged. However, we established a strict and predetermined mIHC pipeline, ensuring well-defined patient selection and justifying the internal validity of this study. Notably, all tumor tissue specimens in the ICI cohort were obtained without any treatment between acquisition and ICI treatment. The mIHC staining was performed in a diligent manner, preserving high sample quality to warrant reliable results. Subsequent analyses were performed in a consistent manner using a highly sophisticated computer-driven analysis. The strength of this study was further reinforced by concomitant transcriptomic and genomic data incorporation. Our comprehensive examination of the TME using proteotyping retaining single-cell spatial information is thus noteworthy.

### Conclusion

We performed an unprecedented TME profiling using diligent and consistent spatial single-cell proteotyping alongside transcriptomic and genomic profiling by dissecting complex TME features in terms of ICI efficacy in mNSCLC. Our findings facilitate a better understanding of the TME profile in NSCLC, thus paving the way for further development of next-generation therapy.

## Methods

Details for patient recruitment, data collection, mIHC staining protocol, digital analysis, in-house transcriptomic analysis, external RNA-seq cohort analysis, and TCGA dataset–based transcriptomic validation are described in Supplemental Methods.

### Sex as a biological variable.

Our study included both female and male patients, as shown in Supplemental Table 1. Findings are expected to apply to both sexes.

### Statistics.

We categorized patients in the ICI cohort into 2 groups based on their PFS for PD-1/PD-L1 inhibitors. They were classified as DCB if they experienced PFS for ≥ 1 year or as non-DCB if they did not. No patients were censored within 12 months except for 2 patients in this cohort. The median value was used to define high versus low levels for continuous variables. Fisher’s exact test and Mann-Whitney *U* test were applied to compare categorical or continuous variables, respectively. Correlations were determined by Spearman’s rank correlation coefficient test. Differences in PFS or OS curves, constructed by the Kaplan-Meier method, were assessed with the log-rank test. Univariable and multivariable Cox proportional hazard regression models were adopted to determine HRs. Covariables for the multivariable analysis were predetermined based on clinical importance in relation to PD-1/PD-L1 inhibitor efficacy. Missing data were not imputed. All *P* values were based on the 2-sided hypothesis. The Benjamini-Hochberg test was performed to calculate the FDR (*q* value). Hierarchical clustering was performed using Cluster 3.0, where the average linkage method was executed using an uncentered correlation similarity metric, and clustering results were visualized by Java TreeView. Since the study was designed as an exploratory biomarker study, no threshold of *P* value was defined for statistical significance. Statistical analysis was performed with GraphPad Prism software version 10.6.0 (GraphPad Software).

### Study approval.

This retrospective study was approved by the IRB of Kindai University Hospital (reference number R02-174). All patients provided written informed consent, where applicable, or such informed consent was waived by IRB-approved protocols. The study was performed in accordance with the Declaration of Helsinki.

### Data availability.

The data underlying this study are not publicly available as stipulated by the study protocol because of the possibility that patient privacy or consent could be compromised. These data are available from the corresponding author upon reasonable request. Aggregated data and source data underlying the main analyses, including multivariable regression analyses, are provided in the Supporting Data Values file.

## Author contributions

KH conceptualized the study and designed experiments. KH and TT supervised mIHC experiments. KI and TT developed mIHC analysis software. KI and KH performed mIHC experiments and TCGA analysis, curated genomic data, analyzed the data, visualized results, and wrote the manuscript. AI curated pathology specimen and assisted with interpretation of mIHC samples. KH performed IO360 transcriptome experiments. ST supported bioinformatic analyses of external validation RNA-seq datasets obtained via dbGaP. KI and YM collected data from clinical databases. MT, KY, and KS assisted curation of genomic data. KH, TT, MT, KY, KT, TI, KS, K Nishio, AI, K Nakagawa, and HH provided resources and reviewed the manuscript. KH and K Nakagawa acquired funding and supervised the study. KI and KH contributed equally to this study as co–first authors. The contribution to performing laborious mIHC experiments and mIHC data preparation was mainly provided by KI; therefore, KI deserves the first position among the co–first authors. Analysis and interpretation of the mIHC data were jointly performed by KI and KH. KH also performed essential mIHC experiments, including pilot studies required for establishing the entire platform, conducted most of the experiments and analyses related to the transcriptome, and wrote most parts of the original draft of the manuscript as co–first author. KH guided KI and the overall study as the sole corresponding author. All authors reviewed and approved the final version.

## Conflict of interest

The conflict-of-interest statement is available in the supplemental materials.

## Funding support

- Osaka Cancer Society.
- KANAE Foundation for the Promotion of Medical Science.
- SGH Foundation.
- YOKOYAMA Foundation for Clinical Pharmacology (grant YRY-2003).

## Supplementary Material

Supplemental data

ICMJE disclosure forms

Supporting data values

## Version 1 03/10/2026

In-Press Preview

## Version 2 05/15/2026

Electronic publication
