# Supplementary material for: Spatial single-cell proteotyping reveals immunotherapy-resistant features within the complex tumor microenvironment of metastatic NSCLC
Source: J Clin Invest. 2026 Mar 10;136(10):e195021. doi: 10.1172/JCI195021 (PMC13178657; doi:10.1172/JCI195021)
Supplement: Supplemental data [file jci-136-195021-s238.pdf]

Supplemental Material

Supplemental Methods……………………………………………………….2
Supplemental Data…………………………………………………………..10
Supplemental Figures……………………………………………………….11
Supplemental Tables………………………………………………………..35
Author contributions and Acknowledgements……………………………43
Conflict-of-interest statement………………………………………………44

Supplemental Methods

Patients

We consecutively reviewed the medical records of all adult (≥20 years old) patients with advanced or recurrent non-small cell lung cancer (NSCLC) at Kindai University Hospital, focusing on those who underwent first-line treatment between October 2006 and September 2021. Our study included 819 patients with available clinical histories and archival tumor tissue specimens for research purposes. Among them, we selected 232 patients treated with programmed cell death 1/programmed cell death ligand 1 (PD-1/PD-L1) inhibitors for tumors, which were wild-type for the *EGFR*-mutations or the *anaplastic lymphoma kinase* (*ALK*)-fusion genes, to evaluate the association of tumor microenvironment (TME) profiles with the treatment efficacy. Additionally, we extracted 194 patients with tumors harboring these driver oncogenes were also extracted to investigate TME profiles related to immune checkpoint inhibitor (ICI) tolerance, as suggested by previous studies (1-3). Exclusion criteria comprised patients whose tumor tissues were obtained by transbronchial needle aspiration or were cell block specimens, as these were deemed unsuitable for our spatial TME analysis. FFPE tumor tissue blocks with insufficient amounts (<4 mm^2/section) were also excluded. For ICI-treated cases, patients receiving any anti-cancer treatment (chemotherapy or radiotherapy) for stage IV lesions between tumor tissue acquisition and PD-1/PD-L1 inhibitor treatment were excluded. Moreover, tumors negative for pan-cytokeratin (panCK) staining among those stained with our multiplex IHC (mIHC) were not included in formal analyses, as they were deemed unsuitable for our platform, as explained below. Ultimately, we identified 71 patients with *EGFR/ALK*-wild-type tumors (*EGFR/ALK*-wt cohort), 19 patients harboring *EGFR* mutations (*EGFR*-mutant cohort), and 13 patients with *ALK*-fusion genes (*ALK*-fusion cohort), all deemed potentially evaluable. From those positive for *EGFR* mutations, we identified 10 patients also treated with PD-1/PD-L1 inhibitors. Thus, the ICI cohort included 81 patients (71 patients from the *EGFR/ALK*-wt cohort and 10 from the *EGFR*-mutant cohort). The details of the patient recruitment process are shown in **Supplemental Figure 1**. Information on race and ethnicity was not collected in this

retrospective study, since such data are not routinely recorded in the medical records in Japan as Japanese regulatory (Ministry of Health, Labour and Welfare [MHLW]) does not require it.

Data collection.

Clinicopathologic features and treatment history data, updated as of June 1, 2022, were extracted from medical records. Genomic features were obtained by NGS of biopsied tumors with >10% of tumor content, utilising various panels, such as FoundationOne CDx (Foundation Medicine), Oncomine Dx Target Test Multi-CDx System (Thermo Fisher Scientific), Ion AmpliSeq Cancer Hotspot Panel v2 (Thermo Fisher Scientific), Ion AmpliSeq Colon and Lung Cancer Panel (Thermo Fisher Scientific), FusionPlex Comprehensive Thyroid and Lung Kit (Thermo Fisher Scientific), or Ion AmpliSeq RNA Fusion Lung Cancer Research Panel (Thermo Fisher Scientific). These sequencing procedures were carried out in clinical sequencing or relevant studies, with secondary data use permitted under written informed consent (4). Seventeen known driver oncogenes were consistently covered by all the next-generation sequencing (NGS) panels (**Supplemental Table 6**). Tumors with any alterations for these 17 genes were defined driver-oncogene^{+} if those alterations were ranked as oncogenic by OncoKB (https://www.oncokb.org/). Treatment responses were assessed using the Response Evaluation Criteria in Solid Tumors (RECIST) v1.1. We calculated progression-free survival (PFS) from the initiation of PD-1/PD-L1 inhibitors treatment to clinical or radiographic progression or death from any cause. Patients without documented clinical or radiographic disease progression or those still alive were censored on the date of the last follow-up. Overall survival (OS) was measured from enrollment to death from any cause, with patients still alive censored at the last follow-up date.

mIHC staining protocol.

We sectioned FFPE tissues into two 4-μm-thick serial sections for all cases. To ensure high-quality staining, the staining for the first sections was started immediately after preparation, and all multiplex staining rounds were completed within 10 days (Panel 1). During the Panel 1

staining, additional sections for Panel 2 were stored with tissue protectors (Matsunami Glass Ind. Ltd.) in dark boxes at 4°C to avoid epitope alteration. Once the Panel 1 staining was completed, Panel 2 staining was immediately started, and all multiplex staining rounds for Panel 2 were also completed within 10 days.

We performed mIHC staining as described previously (5-10). Antibodies and reagents, and their order of use are detailed in **Supplemental Table 7**. Sections of FFPE tissue were deparaffinized, stained with hematoxylin (S3301, Dako), and then subjected to whole-tissue scanning with a NanoZoomer instrument (Hamamatsu Photonics) at 20× magnification to identify the nucleus of each cell. Endogenous peroxidase activity was then blocked by incubation in 0.6% hydrogen peroxide in phosphate buffered saline (PBS) for 15 min. Antigen retrieval was performed by microwave radiation (to achieve a temperature of 95°C for 15 min) with citrate buffer pH 6.0 (RM102-C, LSI Medience). The slides were then exposed to 5.0% goat serum and 2.5% bovine serum albumin (BSA) in PBS to block nonspecific sites, followed by sequential incubation with primary antibodies, anti-mouse, anti-rabbit, anti-rat, and anti-goat Histofine Simple Stain MAX PO horseradish peroxidase-conjugated polymer (Nichirei Biosciences), and the alcohol-soluble peroxidase substrate 3-aminomethyl carbazole (AEC). Goat serum was removed from the blocking buffer before the anti-goat secondary antibody was used. Stained sections were scanned by the NanoZoomer at 20× magnification. Chromogenic destaining and antibody stripping were performed between the sequential staining steps.

Digital analysis.

Representative fields (0.50 to 3.55 mm^2) were randomly selected from intratumoral areas as regions of interest (ROIs) using Aperio ImageScope v.12 software (Leica Biosystems). A board-certified pathologist confirmed the presence of tumor cells in these areas. Between one and four ROIs were evaluated depending on the size of the tumor (average of 2.2 ROIs). Blank areas lacking cellular components were removed from all ROIs using ImageJ/Fiji version 1.51 (National Institutes of Health) to retain evaluable tumor regions for subsequent analyses. The

tumor regions were further categorized into tumor cell nest area and intratumoral stromal area based on panCK staining positivity of cancer cells, utilising a mathematical morphological approach with the Tissue Segmentation platform developed in our previous study (8, 9, 11). Image processing and subsequent computational analysis were performed with ImageJ/Fiji, CellProfiler version 2.2.0 (Broad Institute), Aperio ImageScope, and FCS Express 7 Image Cytometry v.7.04.0020 (De Novo Software) using in-house software (developed by SCREEN Holdings Co., Ltd.) as shown previously (9). This resulted in the quantification of staining intensity for 16–17 protein markers on single cells in each section. Fluorescence-minus-one controls were used as negative controls to determine true positive cells. The final value for each cell number was calculated as the average of values from the multiple ROIs. Visualization of each staining was performed by pseudocoloring with ImageJ/Fiji and Aperio ImageScope. The ImageJ/Fiji macro for tissue segmentation and image preparation for FCS Express analysis is publicly available under the GNU General Public License version 3 at https://github.com/mIHC-Kindai/17-plex-IHC. Detailed usage instructions are provided in the repository's README_ImageCytometry.pdf.

Transcriptomic analysis.

Transcriptomic analysis was performed when sufficient tumor tissues were available for RNA extraction. RNA was extracted from FFPE tumor tissue and subjected to gene expression analysis, following procedures outlined in our previous study (12). Macrodissection of tumor tissue was performed to avoid contamination with non-tumor tissue before isolating intratumoral RNA using an AllPrep DNA/RNA FFPE Kit (Qiagen). The isolated RNA quality was assessed using a NanoDrop system (Thermo Fisher Scientific) and an Agilent 2100 Bioanalyzer system (Agilent Technologies) prior to analysis with the nCounter platform and a PanCancer IO 360 Panel, which includes 750 immune-related genes and 20 housekeeping genes (NanoString Technologies). Sample-to-sample normalization of gene expression was facilitated based on data for the 20 housekeeping genes, using nSolver Analysis Software 4.0 and nCounter Advanced

Analysis 2.0 (NanoString Technologies). Samples with abnormal normalized expression values (normalization factor of >10 obtained with nSolver Analysis Software 4.0) were excluded, as recommended by the manufacturer. A total of 29 samples remained for further analysis. Of the 750 immune-related genes, we filtered out 24 genes, for which >90% of samples had expression values below the minimum threshold. The normalized gene expression data were log2-transformed before calculating the Z score. Signature scores for gene set analysis were calculated using nCounter Advanced Analysis 2.0.

External RNA-sequencing cohort analysis.

RNA sequencing data were obtained from the database of Genotypes and Phenotypes (dbGaP) using the Run Selector for the project Identifying Lung Cancer Microenvironment Features Underlying Immune Checkpoint Inhibitor Response (accession number phs002822). Samples were selected under the following conditions, analyte type = RNA and Consent = GRU, and a total of 45 sequence read archive (SRA) files were downloaded. The SRA files were converted to FASTQ format using fasterq-dump from the SRA Toolkit (version 3.0.7) with default parameters. The resulting FASTQ files were aligned to the human reference genome (GRCh38.p14) using CLC Genomics Workbench (version 25.0.3, QIAGEN) with default alignment settings. Gene expression levels were quantified and normalized as transcripts per million (TPM). Phenotype data were retrieved from dbGaP using the File Selector under the same consent condition (Consent = GRU). The phenotype file (phs002822.v1.pht012388.v1.p1.c3.SU2C_MARK_Subject_Phenotypes.GRU.txt) was used for downstream survival analysis. All dbGaP data were accessed and analyzed in accordance with controlled-access data use policies and relevant institutional approvals.

TCGA dataset–based transcriptomic validation.

Normalized gene expression data (RNA Seq v2 RSEM) for the lung adenocarcinoma cohort (n = 566) and lung squamous cell carcinoma cohort (n = 487) from TCGA dataset were downloaded

from the cBioPortal website (http://www.cbioportal.org) in July 2025 (13). Among these, 991 cases had both RNA sequencing results and *EGFR/ALK* mutational profiles (**Supplemental Figure 13A**). *EGFR* variants which were annotated as oncogenic according to OncoKB (https://www.oncokb.org/) (14, 15) were considered *EGFR*-oncogenes. Other *EGFR* mutations were regarded as variants of uncertain significance (VUS) and thus grouped into wild-type. Cases with *EML4-ALK* fusions were considered *ALK*-oncogene positive. For subsequent differential gene expression analyses, each expression value (x) was transformed to $\log_2(x + 1)$. Of the 20,531 genes, those not expressed in any case were removed, resulting in 20,247 genes retained for the analyses.

Statistics.

We categorized patients in the ICI cohort into two groups based on their PFS for PD-1/PD-L1 inhibitors. They were classified as DCB if they experienced PFS for ≥1 year or as non-DCB if they did not. No patients were censored within 12 months except for 2 patients in this cohort. The median value was used to define high vs low levels for continuous variables. Fisher's exact test and the Mann–Whitney U test were applied to compare categorical or continuous variables, respectively. Correlations were determined by Spearman's rank correlation coefficient test. Differences in PFS or OS curves, constructed by the Kaplan-Meier method, were assessed with the log-rank test. Univariable and multivariable Cox proportional hazard regression models were adopted to determine hazard ratios. Covariables for the multivariable analysis were predetermined based on clinical importance in relation to PD-1/PD-L1 inhibitor efficacy. Missing data were not imputed. All *P* values were based on the two-sided hypothesis. The Benjamini–Hochberg test was performed to calculate the false discovery rate (*q*-value). Hierarchical clustering was performed using Cluster 3.0, where the average linkage method was executed using an uncentred correlation similarity metric, and clustering results were then visualized by Java TreeView. Since the study was designed as an exploratory biomarker study,

no threshold of *P* value was defined for statistical significance. Statistical analysis was performed with GraphPad Prism software version 10.6.0 (Graph Pad Software).

Supplemental Methods References

1. Qiao M, Jiang T, Liu X, Mao S, Zhou F, Li X, et al. Immune Checkpoint Inhibitors in EGFR-Mutated NSCLC: Dusk or Dawn? *J Thorac Oncol.* 2021;16(8):1267-88.
2. Wiest N, Majeed U, Seegobin K, Zhao Y, Lou Y, and Manochakian R. Role of Immune Checkpoint Inhibitor Therapy in Advanced EGFR-Mutant Non-Small Cell Lung Cancer. *Front Oncol.* 2021;11:751209.
3. Hayashi H, Sugawara S, Fukuda Y, Fujimoto D, Miura S, Ota K, et al. A Randomized Phase II Study Comparing Nivolumab with Carboplatin-Pemetrexed for EGFR-Mutated NSCLC with Resistance to EGFR Tyrosine Kinase Inhibitors (WJOG8515L). *Clin Cancer Res.* 2022;28(5):893-902.
4. Takeda M, Sakai K, Terashima M, Kaneda H, Hayashi H, Tanaka K, et al. Clinical application of amplicon-based next-generation sequencing to therapeutic decision making in lung cancer. *Ann Oncol.* 2015;26(12):2477-82.
5. Tsujikawa T, Kumar S, Borkar RN, Azimi V, Thibault G, Chang YH, et al. Quantitative Multiplex Immunohistochemistry Reveals Myeloid-Inflamed Tumor-Immune Complexity Associated with Poor Prognosis. *Cell Rep.* 2017;19(1):203-17.
6. Gopalakrishnan V, Spencer CN, Nezi L, Reuben A, Andrews MC, Karpinets TV, et al. Gut microbiome modulates response to anti-PD-1 immunotherapy in melanoma patients. *Science.* 2018;359(6371):97-103.
7. Tsujikawa T, Crocenzi T, Durham JN, Sugar EA, Wu AA, Onners B, et al. Evaluation of Cyclophosphamide/GVAX Pancreas Followed by Listeria-Mesothelin (CRS-207) with or without Nivolumab in Patients with Pancreatic Cancer. *Clin Cancer Res.* 2020;26(14):3578-88.

8. Makutani Y, Kawakami H, Tsujikawa T, Yoshimura K, Chiba Y, Ito A, et al. Contribution of MMP14-expressing cancer-associated fibroblasts in the tumor immune microenvironment to progression of colorectal cancer. *Front Oncol.* 2022;12:956270.

9. Isomoto K, Haratani K, Tsujikawa T, Makutani Y, Kawakami H, Takeda M, et al. Mechanisms of primary and acquired resistance to immune checkpoint inhibitors in advanced non-small cell lung cancer: A multiplex immunohistochemistry-based single-cell analysis. *Lung Cancer.* 2022;174:71-82.

10. Liudahl SM, Betts CB, Sivagnanam S, Morales-Oyarvide V, da Silva A, Yuan C, et al. Leukocyte Heterogeneity in Pancreatic Ductal Adenocarcinoma: Phenotypic and Spatial Features Associated with Clinical Outcome. *Cancer Discov.* 2021;11(8):2014-31.

11. Yoshimura K, Tsujikawa T, Mitsuda J, Ogi H, Saburi S, Ohmura G, et al. Spatial Profiles of Intratumoral PD-1(+) Helper T Cells Predict Prognosis in Head and Neck Squamous Cell Carcinoma. *Front Immunol.* 2021;12:769534.

12. Haratani K, Nakamura A, Mamesaya N, Mitsuoka S, Yoneshima Y, Saito R, et al. Tumor Microenvironment Landscape of NSCLC Reveals Resistance Mechanisms for Programmed Death-Ligand 1 Blockade After Chemoradiotherapy: A Multicenter Prospective Biomarker Study (WJOG11518L:SUBMARINE). *J Thorac Oncol.* 2023;18(10):1334-50.

13. de Bruijn I, Kundra R, Mastrogiacomo B, Tran TN, Sikina L, Mazor T, et al. Analysis and Visualization of Longitudinal Genomic and Clinical Data from the AACR Project GENIE Biopharma Collaborative in cBioPortal. *Cancer Res.* 2023;83(23):3861-7.

14. Chakravarty D, Gao J, Phillips SM, Kundra R, Zhang H, Wang J, et al. OncoKB: A Precision Oncology Knowledge Base. *JCO Precis Oncol.* 2017;2017.

15. Suehnholz SP, Nissan MH, Zhang H, Kundra R, Nandakumar S, Lu C, et al. Quantifying the Expanding Landscape of Clinical Actionability for Patients with Cancer. *Cancer Discov.* 2024;14(1):49-65.

Supplemental Data

Figure 4B. Covariates included in the multivariable Cox proportional hazards regression model. Female sex (HR, 0.36 [95% CI, 0.16–0.78]), Age ≥75 (HR, 0.65 [95% CI, 0.31–1.30]), PS = 2 (vs PS = 0–1) (HR, 9.01 [95% CI, 3.17–24.5]), Never smoker (HR, 1.79 [95% CI, 0.71–4.17]), De novo metastasis (vs recurrent disease) (HR, 1.77 [95% CI, 0.87–3.81]), Brain metastasis (HR, 1.08 [95% CI, 0.51–2.21]), ICI monotherapy (vs combo) (HR, 1.86 [95% CI, 0.96–3.60]), Treatment line ≥3 (HR, 1.01 [95% CI, 0.23–3.85]), Primary tumor biopsied (vs metastatic site) (HR, 1.07 [95% CI, 0.57–2.07]), *EGFR*-oncogene positive (HR, 3.22 [95% CI, 0.83–14.0]), PD-L1-TPS <50 % (HR, 0.63 [95% CI, 0.29–1.36]).

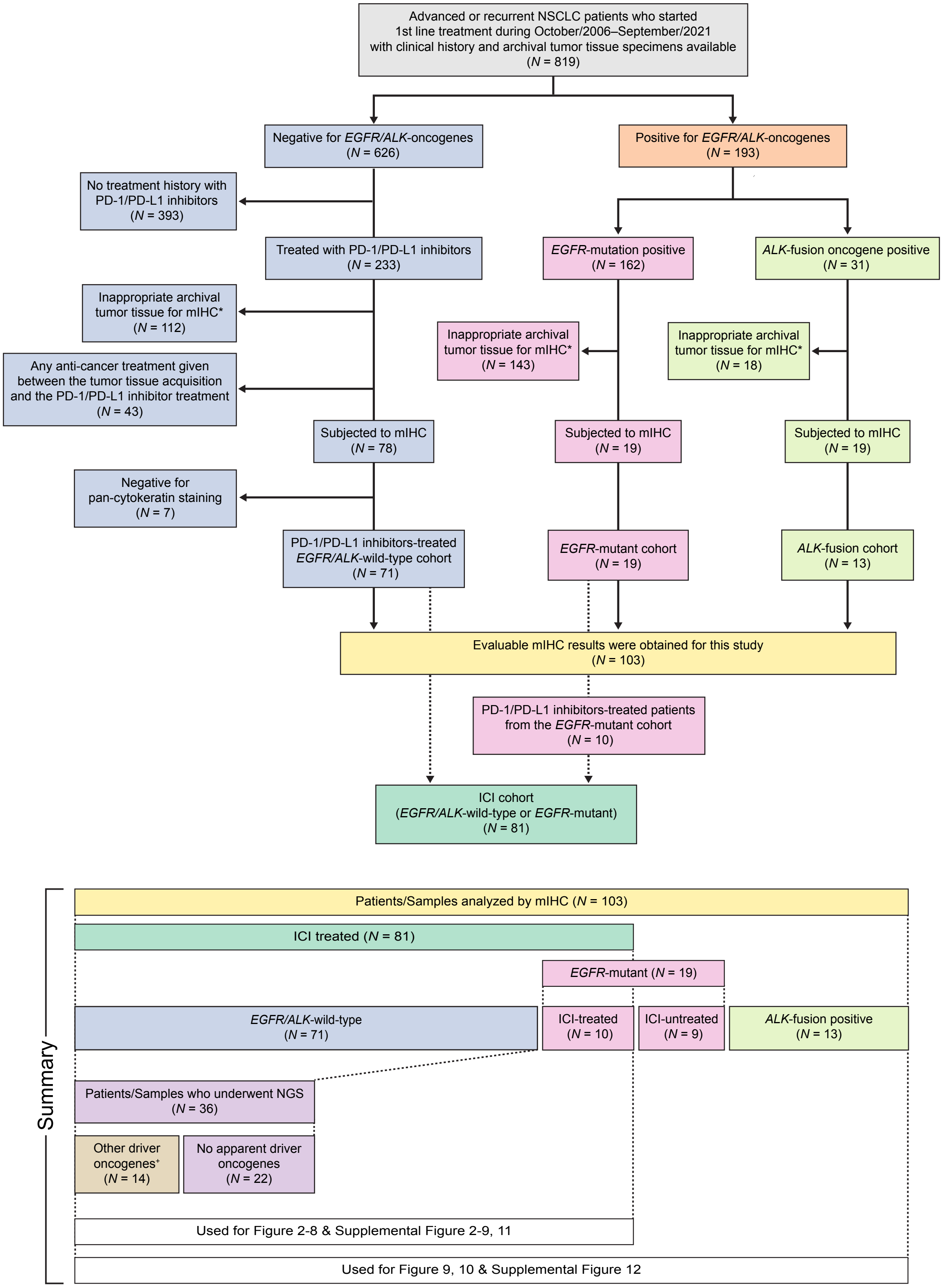
Advanced or recurrent NSCLC patients who started 1st line treatment during October/2006–September/2021 with clinical history and archival tumor tissue specimens available (N = 819)
Negative for EGFR/ALK-oncogenes (N = 626)
Positive for EGFR/ALK-oncogenes (N = 193)
No treatment history with PD-1/PD-L1 inhibitors (N = 393)
Treated with PD-1/PD-L1 inhibitors (N = 233)
EGFR-mutation positive (N = 162)
ALK-fusion oncogene positive (N = 31)
Inappropriate archival tumor tissue for mIHC* (N = 112)
Inappropriate archival tumor tissue for mIHC* (N = 143)
Inappropriate archival tumor tissue for mIHC* (N = 18)
Any anti-cancer treatment given between the tumor tissue acquisition and the PD-1/PD-L1 inhibitor treatment (N = 43)
Subjected to mIHC (N = 78)
Subjected to mIHC (N = 19)
Subjected to mIHC (N = 19)
Negative for pan-cytokeratin staining (N = 7)
PD-1/PD-L1 inhibitors-treated EGFR/ALK-wild-type cohort (N = 71)
EGFR-mutant cohort (N = 19)
ALK-fusion cohort (N = 13)
Evaluable mIHC results were obtained for this study (N = 103)
PD-1/PD-L1 inhibitors-treated patients from the EGFR-mutant cohort (N = 10)
ICI cohort (EGFR/ALK-wild-type or EGFR-mutant) (N = 81)
Summary
Patients/Samples analyzed by mIHC (N = 103)
ICI treated (N = 81)
EGFR-mutant (N = 19)
EGFR/ALK-wild-type (N = 71)
ICI-treated (N = 10)
ICI-untreated (N = 9)
ALK-fusion positive (N = 13)
Patients/Samples who underwent NGS (N = 36)
Other driver oncogenes+ (N = 14)
No apparent driver oncogenes (N = 22)
Used for Figure 2-8 & Supplemental Figure 2-9, 11
Used for Figure 9, 10 & Supplemental Figure 12

Supplemental Figure 1. Flow diagram of patient selection. *Specimens obtained through transbronchial needle aspiration (TBNA), cell blocks, or those that were too small for iterative mIHC staining (<4 mm^2/section) were excluded from the mIHC study. The amount of tumor tissue specimens of the stage IV *EGFR*-mutant NSCLC left for research studies was limited due to the frequent prior use of these tissues for our previous studies (PMID: 28407039; PMID: 36347190).

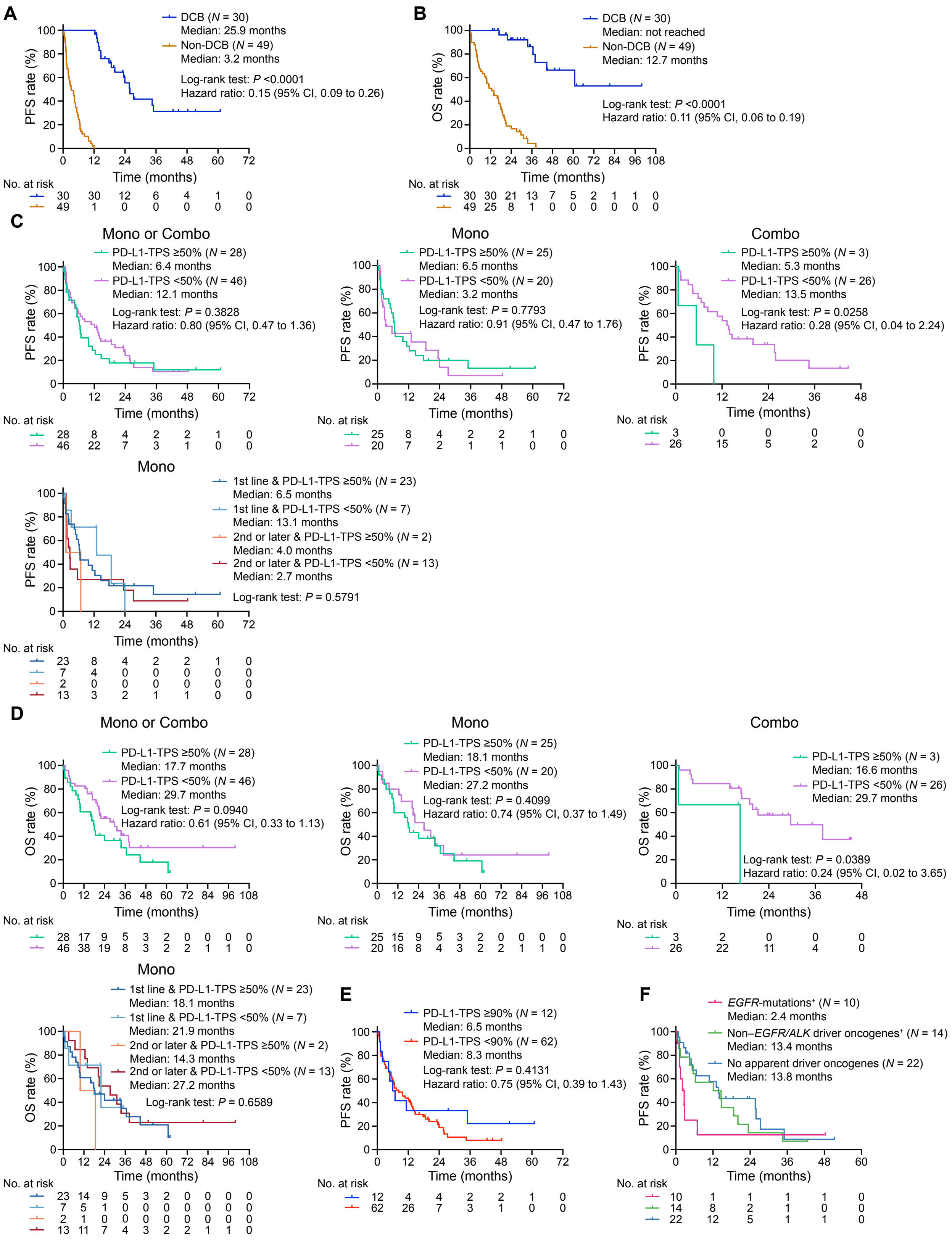

A
DCB (N = 30)
Median: 25.9 months
Non-DCB (N = 49)
Median: 3.2 months
Log-rank test: P <0.0001
Hazard ratio: 0.15 (95% CI, 0.09 to 0.26)
PFS rate (%)
Time (months)
No. at risk
30 30 12 6 4 1 0
49 1 0 0 0 0 0
B
DCB (N = 30)
Median: not reached
Non-DCB (N = 49)
Median: 12.7 months
Log-rank test: P <0.0001
Hazard ratio: 0.11 (95% CI, 0.06 to 0.19)
OS rate (%)
Time (months)
No. at risk
30 30 21 13 7 5 2 1 1 0
49 25 8 1 0 0 0 0 0 0
C
Mono or Combo
PD-L1-TPS ≥50% (N = 28)
Median: 6.4 months
PD-L1-TPS <50% (N = 46)
Median: 12.1 months
Log-rank test: P = 0.3828
Hazard ratio: 0.80 (95% CI, 0.47 to 1.36)
No. at risk
28 8 4 2 2 1 0
46 22 7 3 1 0 0
Mono
PD-L1-TPS ≥50% (N = 25)
Median: 6.5 months
PD-L1-TPS <50% (N = 20)
Median: 3.2 months
Log-rank test: P = 0.7793
Hazard ratio: 0.91 (95% CI, 0.47 to 1.76)
No. at risk
25 8 4 2 2 1 0
20 7 2 1 1 0 0
Combo
PD-L1-TPS ≥50% (N = 3)
Median: 5.3 months
PD-L1-TPS <50% (N = 26)
Median: 13.5 months
Log-rank test: P = 0.0258
Hazard ratio: 0.28 (95% CI, 0.04 to 2.24)
No. at risk
3 0 0 0 0
26 15 5 2 0
Mono
1st line & PD-L1-TPS ≥50% (N = 23)
Median: 6.5 months
1st line & PD-L1-TPS <50% (N = 7)
Median: 13.1 months
2nd or later & PD-L1-TPS ≥50% (N = 2)
Median: 4.0 months
2nd or later & PD-L1-TPS <50% (N = 13)
Median: 2.7 months
Log-rank test: P = 0.5791
No. at risk
23 8 4 2 2 1 0
7 4 0 0 0 0 0
2 0 0 0 0 0 0
13 3 2 1 1 0 0
D
Mono or Combo
PD-L1-TPS ≥50% (N = 28)
Median: 17.7 months
PD-L1-TPS <50% (N = 46)
Median: 29.7 months
Log-rank test: P = 0.0940
Hazard ratio: 0.61 (95% CI, 0.33 to 1.13)
No. at risk
28 17 9 5 3 2 0 0 0 0
46 38 19 8 3 2 2 1 1 0
Mono
PD-L1-TPS ≥50% (N = 25)
Median: 18.1 months
PD-L1-TPS <50% (N = 20)
Median: 27.2 months
Log-rank test: P = 0.4099
Hazard ratio: 0.74 (95% CI, 0.37 to 1.49)
No. at risk
25 15 9 5 3 2 0 0 0 0
20 16 8 4 3 2 2 1 1 0
Combo
PD-L1-TPS ≥50% (N = 3)
Median: 16.6 months
PD-L1-TPS <50% (N = 26)
Median: 29.7 months
Log-rank test: P = 0.0389
Hazard ratio: 0.24 (95% CI, 0.02 to 3.65)
No. at risk
3 2 0 0 0
26 22 11 4 0
Mono
1st line & PD-L1-TPS ≥50% (N = 23)
Median: 18.1 months
1st line & PD-L1-TPS <50% (N = 7)
Median: 21.9 months
2nd or later & PD-L1-TPS ≥50% (N = 2)
Median: 14.3 months
2nd or later & PD-L1-TPS <50% (N = 13)
Median: 27.2 months
Log-rank test: P = 0.6589
No. at risk
23 14 9 5 3 2 0 0 0 0
7 5 1 0 0 0 0 0 0 0
2 1 0 0 0 0 0 0 0 0
13 11 7 4 3 2 2 1 1 0
E
PD-L1-TPS ≥90% (N = 12)
Median: 6.5 months
PD-L1-TPS <90% (N = 62)
Median: 8.3 months
Log-rank test: P = 0.4131
Hazard ratio: 0.75 (95% CI, 0.39 to 1.43)
No. at risk
12 4 4 2 2 1 0
62 26 7 3 1 0 0
F
EGFR-mutations+ (N = 10)
Median: 2.4 months
Non–EGFR/ALK driver oncogenes+ (N = 14)
Median: 13.4 months
No apparent driver oncogenes (N = 22)
Median: 13.8 months
No. at risk
10 1 1 1 1 0
14 8 2 1 0 0
22 12 5 1 1 0

Supplemental Figure 2. ICI efficacy in this study cohort. (A) Kaplan–Meier (KM) curves for PFS of ICI treatment compared between the durable clinical benefit (DCB) and non-DCB groups. **(B)** KM curves for OS of ICI treatment compared between the DCB and non-DCB groups. **(C and D)** KM curves for PFS (C) and OS (D) of ICI treatment stratified by programmed cell death ligand 1 tumor proportion score (PD-L1-TPS), with or without treatment lines (first-line versus second or later lines). Mono indicates ICI monotherapy, and Combo indicates ICI combined with cytotoxic chemotherapy. **(E)** KM curves for PFS of ICI treatment stratified by PD-L1-TPS with a cut off of 90%. **(F)** KM curves for PFS of ICI treatment compared between patients with activating *EGFR* mutations, those with non-*EGFR/ALK* driver oncogene–positive tumors, and those without apparent driver oncogenes. Two cases were not included in (A) and (B), because DCB/non-DCB status could not be determined due to early censoring (<1 year) of PFS data. Seven cases with unavailable PD-L1-TPS clinical data were excluded in (C–E). Thirty-five *EGFR/ALK*–wild-type cases were excluded in (F) because final genomic status remained inconclusive due to lack of next-generation sequencing results. The *P* values for survival analyses were determined with the log-rank test. Vertical bars on the KM curves indicate censoring. Abbreviations: CI, confidence interval.

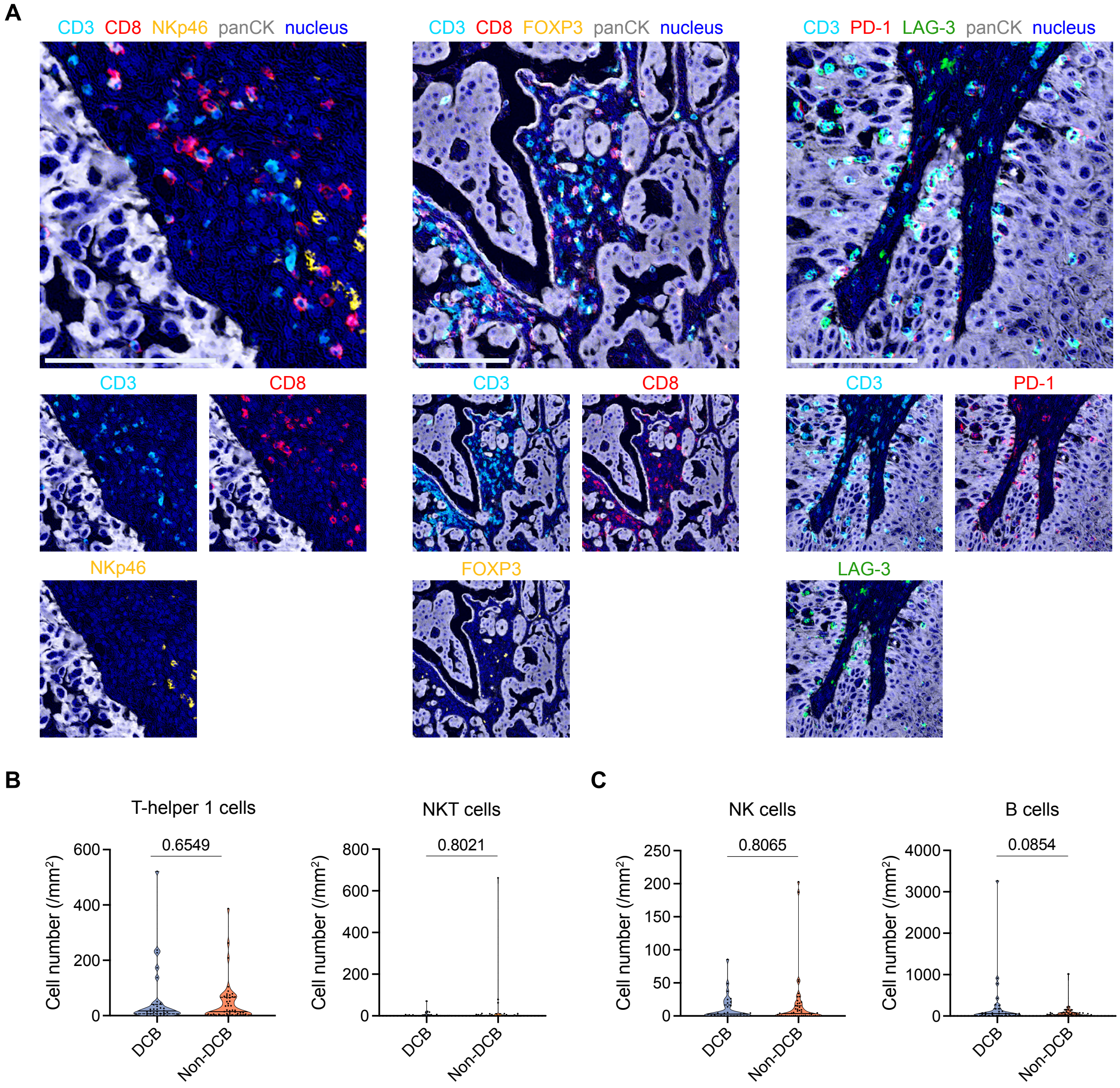

Supplemental Figure 3. Association of non-CD8$^+$ T cells with ICI efficacy. (A) Representative mIHC images of lymphocyte subsets. Multicolor images show CD3 (cyan), CD8 (red), NKp46 (yellow), panCK (white), and nucleus (blue), identifying CD8$^+$ T cells (panCK$^-$CD3$^+$CD8$^+$) and NK cells (panCK$^-$CD3$^-$NKp46$^+$) (left). Images showing CD3 (cyan), CD8 (red), FOXP3 (yellow), panCK (white), and nucleus (blue) identify Tregs (panCK$^-$CD3$^+$CD8$^-$FOXP3$^+$) (middle). Images showing CD3 (cyan), PD-1 (red), LAG-3 (green), panCK (white), and nucleus (blue) depict exhausted T cell subsets (right). Scale bar, 100 μm. **(B**

and C) Densities of intratumoral T-helper 1 cells, NKT cells, NK cells, and B cells are compared between the DCB (N = 30) and non-DCB (N = 49) group. Two cases were not included in (B) and (C) because DCB/non-DCB status could not be determined due to early censoring (<1 year) of PFS data. Data are provided by violin plots in (B) and (C), with each dot represents each patient in the violin plots. The *P* values indicated on horizontal bars were determined with the Mann–Whitney U test.

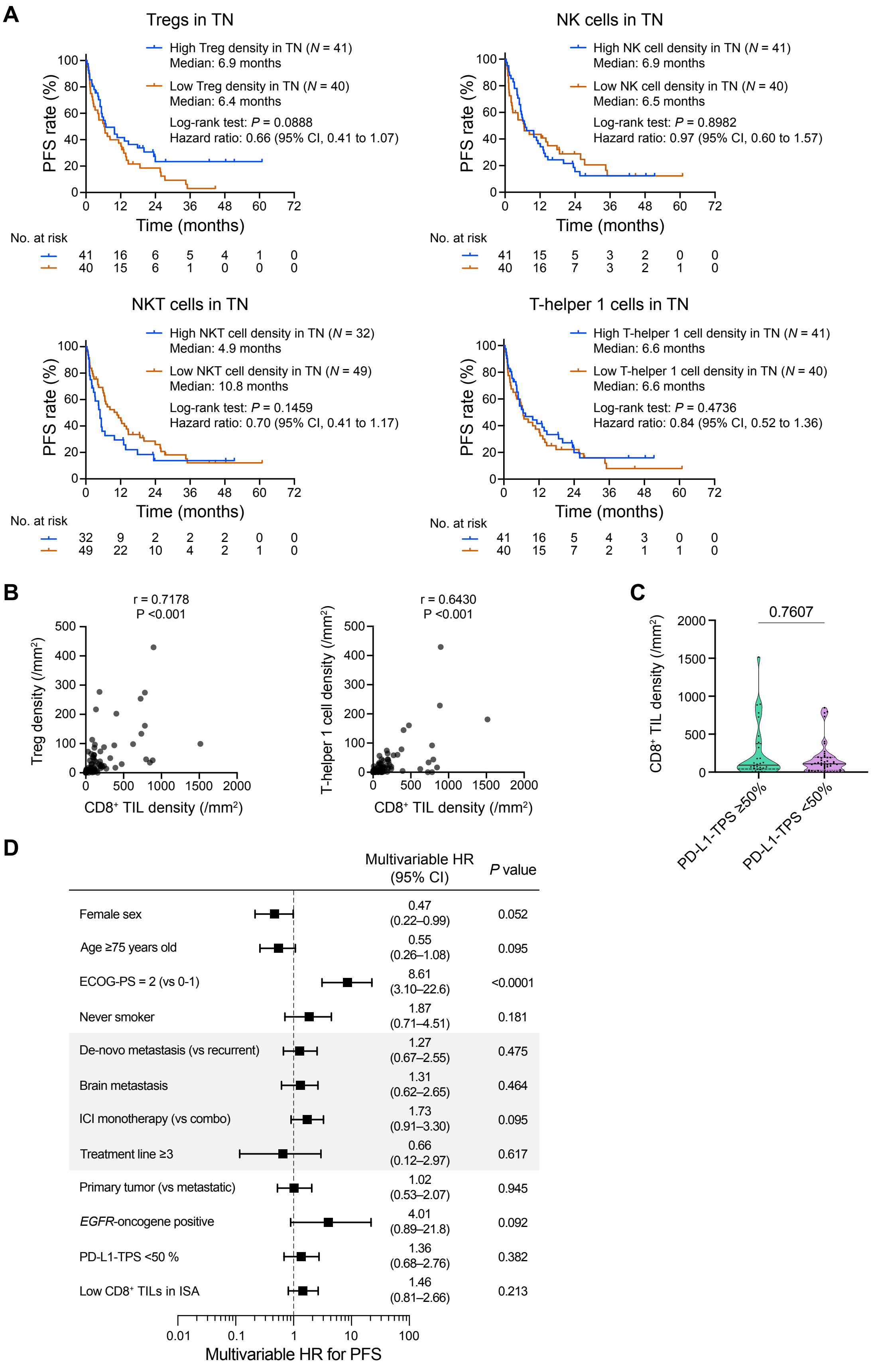
A
Tregs in TN
High Treg density in TN (N = 41)
Median: 6.9 months
Low Treg density in TN (N = 40)
Median: 6.4 months
Log-rank test: P = 0.0888
Hazard ratio: 0.66 (95% CI, 0.41 to 1.07)
PFS rate (%)
Time (months)
No. at risk
41 16 6 5 4 1 0
40 15 6 1 0 0 0
NK cells in TN
High NK cell density in TN (N = 41)
Median: 6.9 months
Low NK cell density in TN (N = 40)
Median: 6.5 months
Log-rank test: P = 0.8982
Hazard ratio: 0.97 (95% CI, 0.60 to 1.57)
PFS rate (%)
Time (months)
No. at risk
41 15 5 3 2 0 0
40 16 7 3 2 1 0
NKT cells in TN
High NKT cell density in TN (N = 32)
Median: 4.9 months
Low NKT cell density in TN (N = 49)
Median: 10.8 months
Log-rank test: P = 0.1459
Hazard ratio: 0.70 (95% CI, 0.41 to 1.17)
PFS rate (%)
Time (months)
No. at risk
32 9 2 2 2 0 0
49 22 10 4 2 1 0
T-helper 1 cells in TN
High T-helper 1 cell density in TN (N = 41)
Median: 6.6 months
Low T-helper 1 cell density in TN (N = 40)
Median: 6.6 months
Log-rank test: P = 0.4736
Hazard ratio: 0.84 (95% CI, 0.52 to 1.36)
PFS rate (%)
Time (months)
No. at risk
41 16 5 4 3 0 0
40 15 7 2 1 1 0
B
r = 0.7178
P <0.001
Treg density (/mm²)
CD8⁺ TIL density (/mm²)
r = 0.6430
P <0.001
T-helper 1 cell density (/mm²)
CD8⁺ TIL density (/mm²)
C
0.7607
CD8⁺ TIL density (/mm²)
PD-L1-TPS ≥50%
PD-L1-TPS <50%
D
Multivariable HR (95% CI)
P value
Female sex 0.47 (0.22–0.99) 0.052
Age ≥75 years old 0.55 (0.26–1.08) 0.095
ECOG-PS = 2 (vs 0-1) 8.61 (3.10–22.6) <0.0001
Never smoker 1.87 (0.71–4.51) 0.181
De-novo metastasis (vs recurrent) 1.27 (0.67–2.55) 0.475
Brain metastasis 1.31 (0.62–2.65) 0.464
ICI monotherapy (vs combo) 1.73 (0.91–3.30) 0.095
Treatment line ≥3 0.66 (0.12–2.97) 0.617
Primary tumor (vs metastatic) 1.02 (0.53–2.07) 0.945
EGFR-oncogene positive 4.01 (0.89–21.8) 0.092
PD-L1-TPS <50 % 1.36 (0.68–2.76) 0.382
Low CD8⁺ TILs in ISA 1.46 (0.81–2.66) 0.213
Multivariable HR for PFS

Supplemental Figure 4. Association of spatial localization of non-CD8$^+$ T cells with ICI efficacy. (A) KM curves for PFS of ICI treatment stratified by the non-CD8$^+$ T cell (Treg, NK cells, NKT cells, and T-helper 1 cells) density in tumor nest (TN). **(B)** Correlation between CD8$^+$ T cell density and Treg density (left) or T-helper 1 cell density (right) in TN (N = 81). Pairwise correlation was calculated using Spearman's correlation analysis. **(C)** Densities of CD8$^+$ T cells infiltrated into TN were compared between their PD-L1-TPS (≥50 %, N =28; <50 %, N =46). Seven cases were excluded because PD-L1-TPS clinical data were not available. **(D)** Forest plot showing HRs estimated by multivariable Cox proportional hazards regression models evaluating the association between PFS of ICI treatment and the indicated biomarkers. Data are provided by violin plots in (C), with *P* values indicated on horizontal bar. Each dot represents each patient in (B) and (C). The *P* values of the violin plots and survival analyses were determined with the Mann–Whitney U test and log-rank test, respectively. Vertical bars on the KM curves indicate censoring. Abbreviations: TIL, tumor-infiltrating lymphocyte.

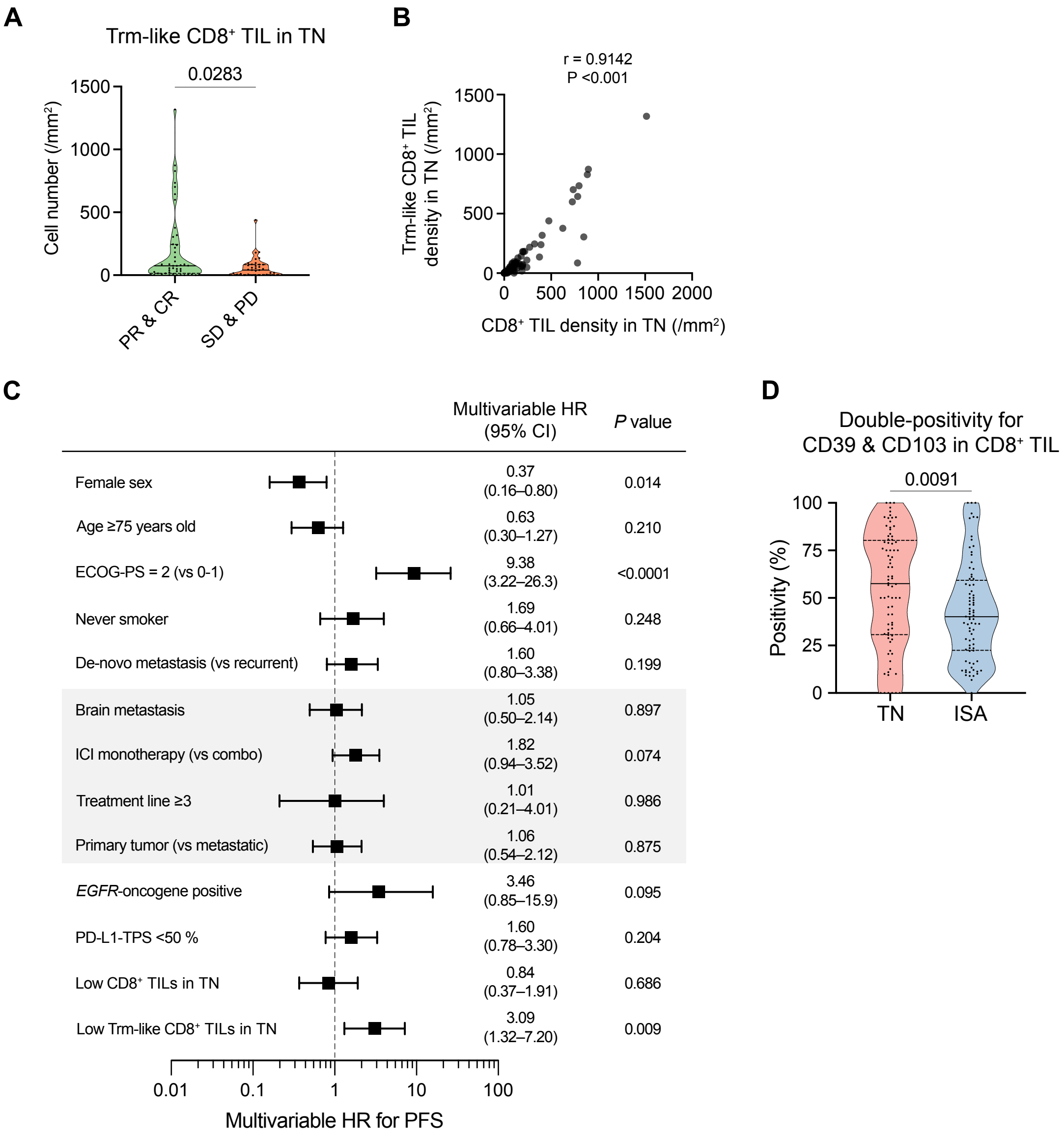

Supplemental Figure 5. Association of tissue resident-memory (Trm)-like CD8$^+$ T cells with ICI efficacy. (A) Densities of Trm-like CD8$^+$ T cells infiltrated into TN compared between objective response of ICI treatment based on the best overall response (PR & CR, N = 44; SD & PD, N =37). **(B)** Correlation between CD8$^+$ TIL density and Trm-like CD8$^+$ TIL density in TN (N = 81). Pairwise correlation was calculated using Spearman's correlation analysis. **(C)** Forest plot showing HRs estimated by multivariable Cox proportional hazards regression models evaluating the association between PFS of ICI treatment and the indicated biomarkers. **(D)**

Double positivity for CD39 and CD103 in $CD8^+$ TILs compared between TN and intratumoral stromal area (ISA) (N =80). One case was excluded, because $CD8^+$ T cells were absent, precluding positivity calculation. Each dot represents each patient in (A), (B), and (D). Data are provided by violin plots in (A) and (D), with *P* values indicated on horizontal bars. The *P* values of the violin plots and survival analyses were determined with the Mann–Whitney U test and log-rank test, respectively. Abbreviations: PR, partial response; CR, complete response; SD, stable disease; PD, progressive disease.

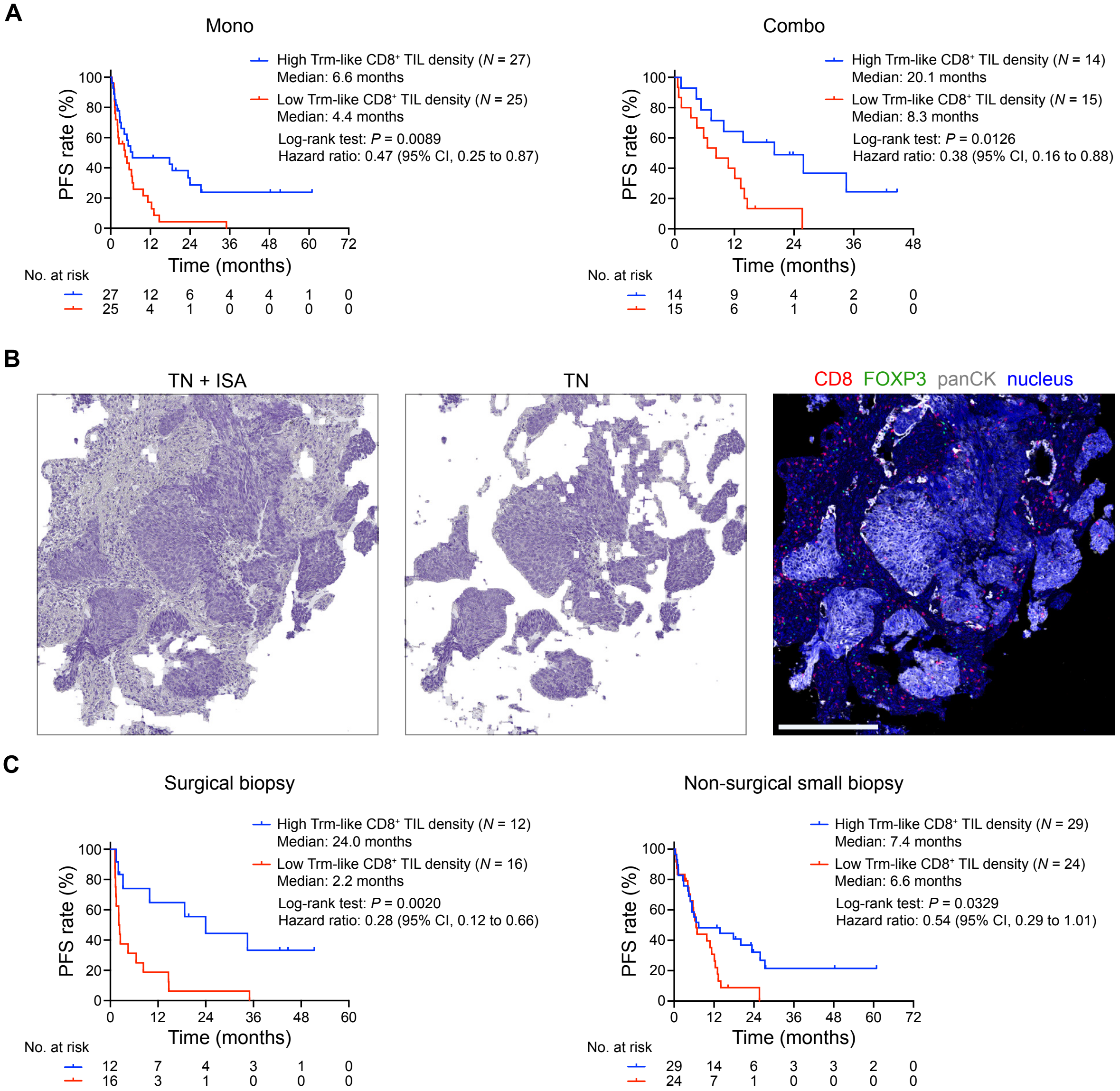

Supplemental Figure 6. Association of treatment regimens and sample collection methods with ICI efficacy. (A) KM curves for PFS of ICI treatment stratified by Trm-like CD8^{+} TIL density in TN across treatment-regimen subgroups. **(B)** Representative images demonstrating appropriate tissue segmentation in a small biopsy sample. Hematoxylin images of TN and ISA (left), TN (middle), and a multicolor image with CD8 (red), FOXP3 (green), panCK (white), and nucleus (blue) are shown on the right. Scale bar, 300 µm. **(C)** KM curves for PFS of ICI treatment stratified by Trm-like CD8^{+} TIL density in TN across sample-collection method

subgroups. The *P* values of survival analyses were determined with log-rank test. Vertical bars on the KM curves indicate censoring.

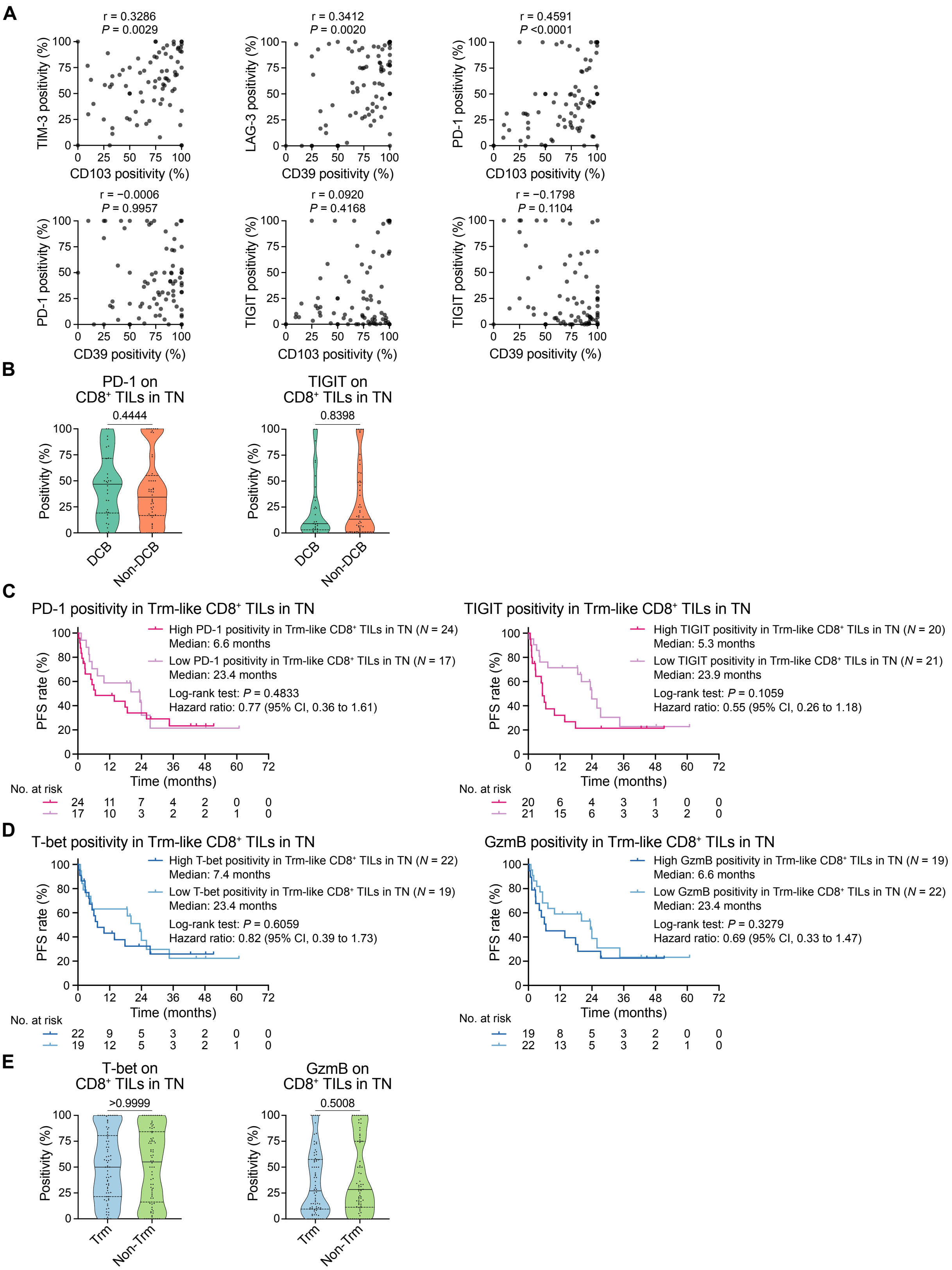
A
r = 0.3286
P = 0.0029
TIM-3 positivity (%)
CD103 positivity (%)
r = 0.3412
P = 0.0020
LAG-3 positivity (%)
CD39 positivity (%)
r = 0.4591
P <0.0001
PD-1 positivity (%)
CD103 positivity (%)
r = −0.0006
P = 0.9957
PD-1 positivity (%)
CD39 positivity (%)
r = 0.0920
P = 0.4168
TIGIT positivity (%)
CD103 positivity (%)
r = −0.1798
P = 0.1104
TIGIT positivity (%)
CD39 positivity (%)
B
PD-1 on CD8+ TILs in TN
0.4444
Positivity (%)
DCB
Non-DCB
TIGIT on CD8+ TILs in TN
0.8398
Positivity (%)
DCB
Non-DCB
C
PD-1 positivity in Trm-like CD8+ TILs in TN
High PD-1 positivity in Trm-like CD8+ TILs in TN (N = 24)
Median: 6.6 months
Low PD-1 positivity in Trm-like CD8+ TILs in TN (N = 17)
Median: 23.4 months
Log-rank test: P = 0.4833
Hazard ratio: 0.77 (95% CI, 0.36 to 1.61)
PFS rate (%)
Time (months)
No. at risk
24 11 7 4 2 0 0
17 10 3 2 2 1 0
TIGIT positivity in Trm-like CD8+ TILs in TN
High TIGIT positivity in Trm-like CD8+ TILs in TN (N = 20)
Median: 5.3 months
Low TIGIT positivity in Trm-like CD8+ TILs in TN (N = 21)
Median: 23.9 months
Log-rank test: P = 0.1059
Hazard ratio: 0.55 (95% CI, 0.26 to 1.18)
PFS rate (%)
Time (months)
No. at risk
20 6 4 3 1 0 0
21 15 6 3 3 2 0
D
T-bet positivity in Trm-like CD8+ TILs in TN
High T-bet positivity in Trm-like CD8+ TILs in TN (N = 22)
Median: 7.4 months
Low T-bet positivity in Trm-like CD8+ TILs in TN (N = 19)
Median: 23.4 months
Log-rank test: P = 0.6059
Hazard ratio: 0.82 (95% CI, 0.39 to 1.73)
PFS rate (%)
Time (months)
No. at risk
22 9 5 3 2 0 0
19 12 5 3 2 1 0
GzmB positivity in Trm-like CD8+ TILs in TN
High GzmB positivity in Trm-like CD8+ TILs in TN (N = 19)
Median: 6.6 months
Low GzmB positivity in Trm-like CD8+ TILs in TN (N = 22)
Median: 23.4 months
Log-rank test: P = 0.3279
Hazard ratio: 0.69 (95% CI, 0.33 to 1.47)
PFS rate (%)
Time (months)
No. at risk
19 8 5 3 2 0 0
22 13 5 3 2 1 0
E
T-bet on CD8+ TILs in TN
>0.9999
Positivity (%)
Trm
Non-Trm
GzmB on CD8+ TILs in TN
0.5008
Positivity (%)
Trm
Non-Trm

Supplemental Figure 7. Association of T cell exhaustion or T cell activation marker positivity in $CD8^+$ TILs with ICI efficacy. (A) Correlation between exhaustion marker positivities, including TIM-3, LAG-3, PD-1, TIGIT, CD39, and CD103, in $CD8^+$ TILs in TN (N = 80). One case was excluded because no $CD8^+$ T cells were absent in the tumor, precluding positivity calculation. Pairwise correlation was calculated using Spearman's correlation analysis. **(B)** PD-1 (left) and TIGIT (right) positivities in $CD8^+$ TILs in TN compared between the DCB (N = 30) and non-DCB (N = 48) groups. Two cases were not included in because DCB/non-DCB status could not be determined due to early censoring (<1 year) of PFS data. Another case was excluded in the non-DCB group, because $CD8^+$ T cells were absent, precluding positivity calculation. **(C)** KM curves for PFS of ICI treatment stratified by PD-1 (left) and TIGIT (right) positivities in tumors with high Trm-like $CD8^+$ TIL density in TN. **(D)** KM curves for PFS of ICI treatment stratified by T-bet (left) and granzyme B (GzmB) (right) positivities in tumors with high Trm-like $CD8^+$ TIL density in TN. **(E)** T-bet (left) and GzmB (right) positivities compared between Trm-like (N = 74) and non-Trm-like (N =76) $CD8^+$ TILs in TN. Seven Trm-like and five non–Trm-like cases were excluded because the corresponding T cell populations were absent, precluding positivity calculation. Each dot represents each patient in (A), (B), and (E). Data are provided by violin plots in (B) and (E), with *P* values indicated on horizontal bars. The *P* values for violin plots and survival analyses were determined with the Mann–Whitney U test and log-rank test, respectively. Vertical bars on the KM curves indicate censoring.

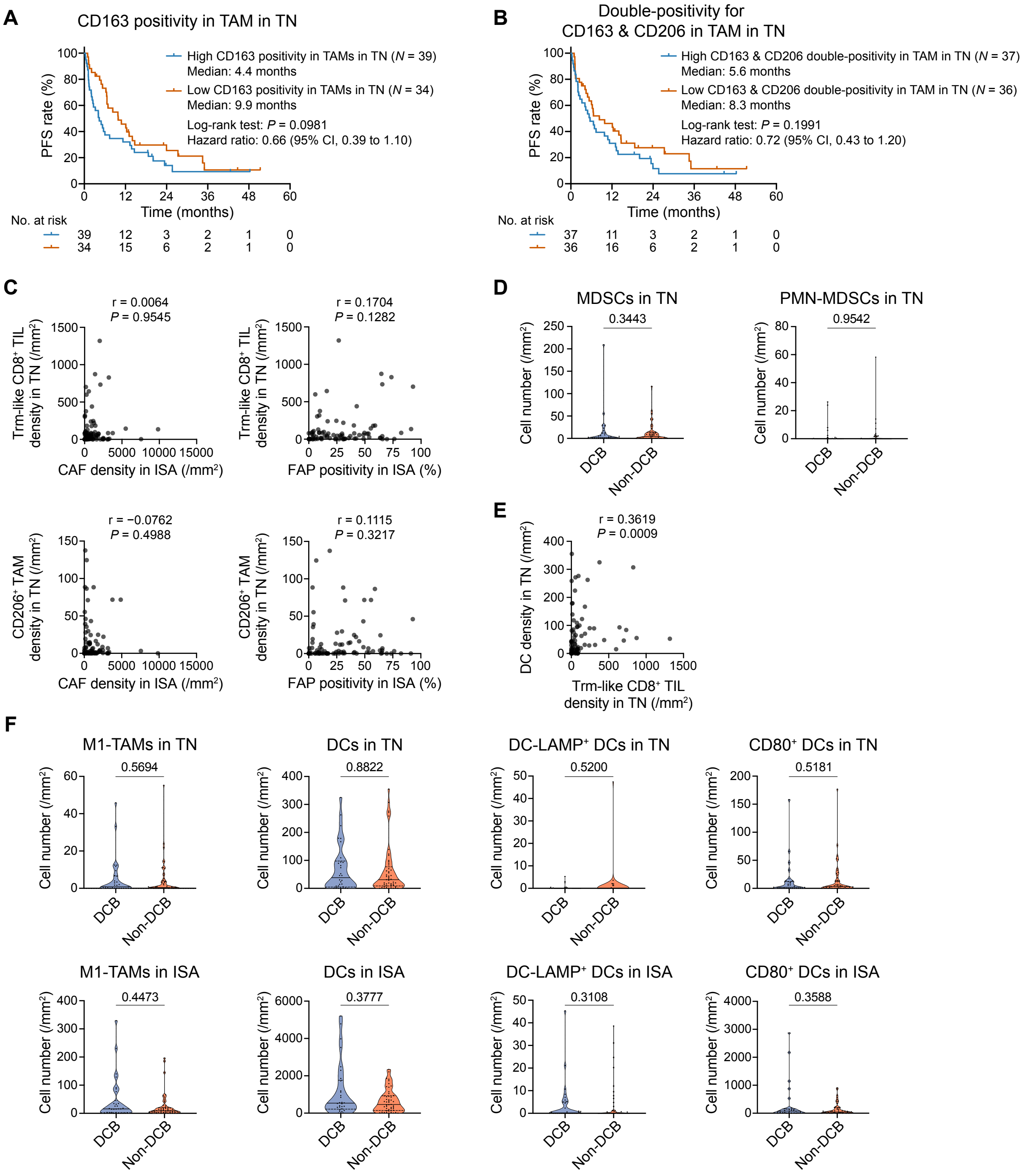

Supplemental Figure 8. Association of tumor-associated macrophages (TAMs), cancer-associated fibroblasts (CAFs), myeloid-derived suppressor cells (MDSCs), and DCs with ICI efficacy. (A) KM curves for PFS of ICI treatment stratified by CD163 positivity in TAMs in TN. **(B)** KM curves for PFS of ICI treatment stratified by double positivity for CD163 and

CD206 in TAMs. **(C)** Correlation between CAF density in the ISA and Trm-like $CD8^+$ TIL density (top left) or $CD206^+$ TAMs density (bottom left) in TN, and correlation between fibroblast activating protein (FAP) positivity in stromal cells and Trm-like $CD8^+$ TIL density (top right) or $CD206^+$ TAMs density (bottom right) in TN (N = 81). **(D)** Densities of MDSCs and PMN-MDSCs in TN compared between the DCB (N = 30) and non-DCB (N = 49) groups. **(E)** Correlation between Trm-like $CD8^+$ TIL density and DC density in TN (N = 81). **(F)** Densities of M1-TAMs and DCs (including DC-$LAMP^+$ and $CD80^+$) in both TN and ISA compared between the DCB (N = 30) and non-DCB (N = 49) groups. Eight cases were excluded in (A) and (B) because $CD68^+$ TAMs were absent, precluding positivity calculation. Two cases were not included in (D) and (F) because DCB/non-DCB status could not be determined due to early censoring (<1 year) of PFS data. Each dot represents each patient in (C–F). Data are provided by violin plots in (D) and (F), with *P* values indicated on horizontal bars. The *P* values for the violin plots and survival analyses were determined with the Mann–Whitney U test and log-rank test, respectively. Pairwise correlation was calculated using Spearman's correlation analysis in (C) and (E). Vertical bars on KM curves indicate censoring. Abbreviations: DC-LAMP, dendritic cell lysosome-associated membrane protein.

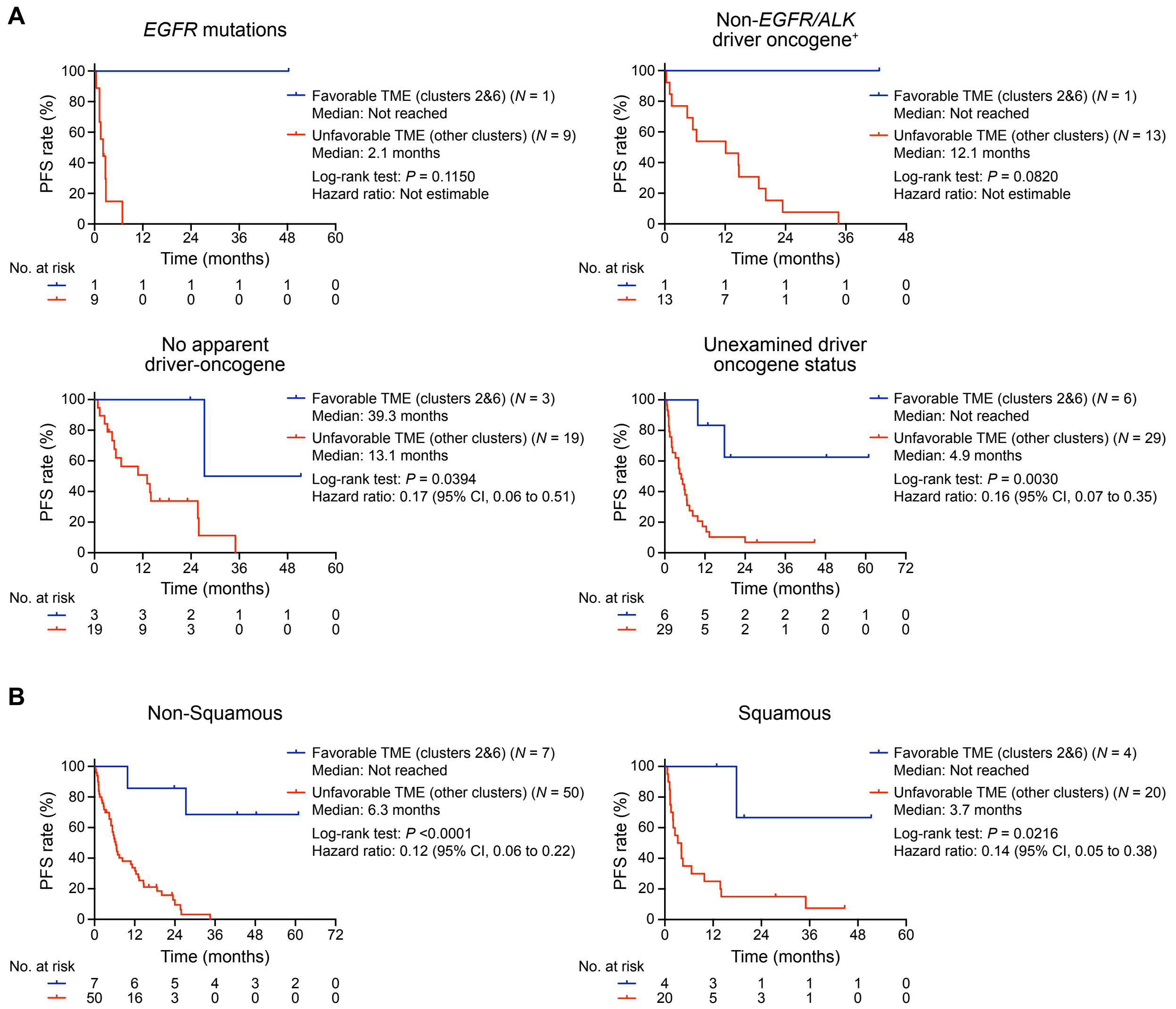

Supplemental Figure 9. Predictive features of the mIHC-based TME profile based on histology and driver oncogene status. (A) KM curves for PFS of ICI treatment, comparing favorable TME (clusters 2 and 6) versus unfavorable TME (other clusters) in patients with *EGFR* mutations (top left), non-*EGFR/ALK* driver oncogenes (top right), and without apparent driver oncogenes (bottom left). Data for patients negative for *EGFR/ALK* oncogenes but with unexamined non-*EGFR/ALK* driver oncogene status are also shown (bottom right). See **Supplemental Tables 2 and 6** for details of genomic features tested. **(B)** KM curves for PFS of ICI treatment, comparing favorable TME (clusters 2 and 6) and unfavorable TME (other

clusters) based on tumor histology. The *P* values were determined with the log-rank test. Vertical bars on the KM curves indicate censoring.

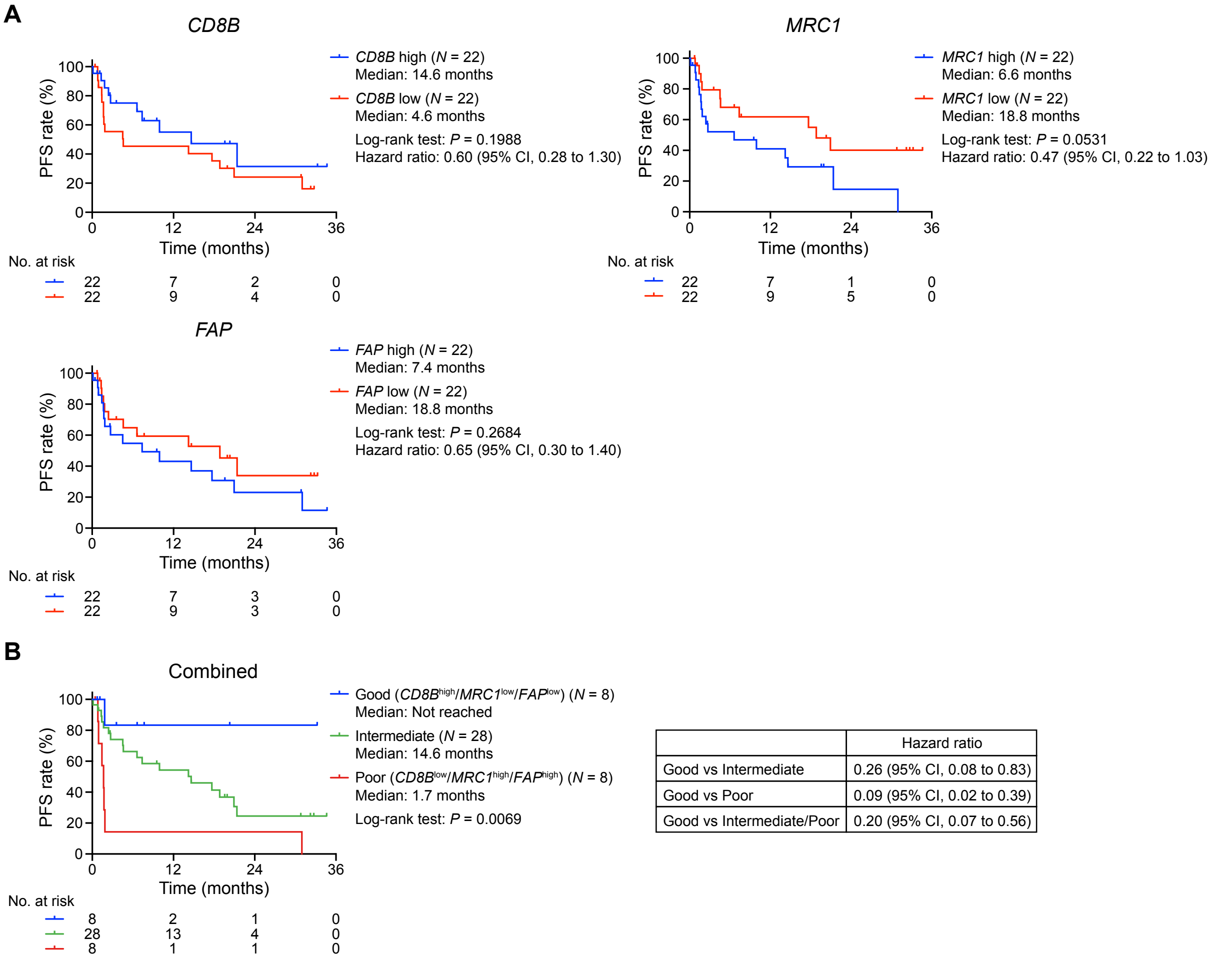

	Hazard ratio
Good vs Intermediate	0.26 (95% CI, 0.08 to 0.83)
Good vs Poor	0.09 (95% CI, 0.02 to 0.39)
Good vs Intermediate/Poor	0.20 (95% CI, 0.07 to 0.56)

Supplemental Figure 10. External validation of mIHC-derived TME features using a bulk RNA-sequencing cohort. (A) KM curves for PFS of ICI treatment stratified by expression levels of *CD8B*, *MRC1* (CD206), and *FAP* in the SU2CLC-MGH bulk RNA-sequencing cohort (N = 44). **(B)** KM curves for PFS based on a composite gene expression profile composed of *CD8B*, *MRC1*, and *FAP* expression. Patients were classified as good ($CD8B^{high}/MRC1^{low}/FAP^{low}$), intermediate (neither good nor poor), or poor ($CD8B^{low}/MRC1^{high}/FAP^{high}$). The *P* values for survival analyses were determined using the log-rank test. Vertical bars on KM curves indicate censoring.

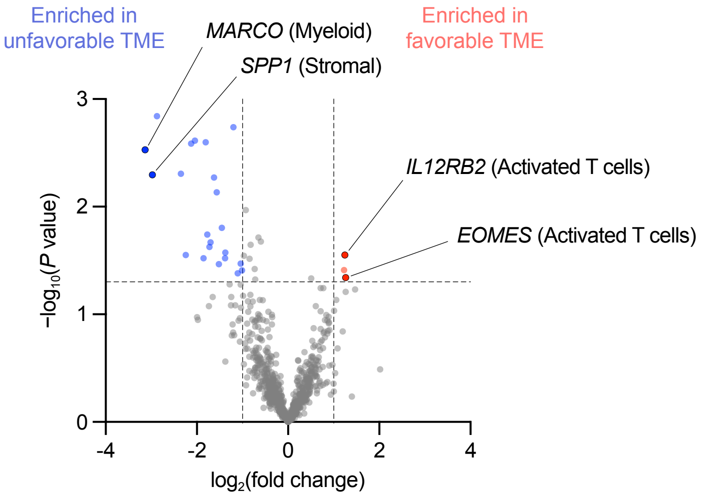

Top 30 downregulated genes

No	Gene	log_2(FC)	*P* value
1	*MARCO*	−3.1350	2.97×10^{-3}
2	*SPP1*	−2.9777	5.04×10^{-3}
3	*PDZK1IP1*	−2.8758	1.44×10^{-3}
4	*OLR1*	−2.3494	4.95×10^{-3}
5	*SERPINA1*	−2.2462	2.82×10^{-2}
6	*ICAM5*	−2.1236	2.59×10^{-3}
7	*SLC11A1*	−2.0413	2.44×10^{-3}
8	*AREG*	−1.9930	1.06×10^{-1}
9	*COL17A1*	−1.9770	1.12×10^{-1}
10	*TREM1*	−1.8563	3.02×10^{-2}
11	*FSTL3*	−1.8073	2.51×10^{-3}
12	*INHBA*	−1.7738	1.82×10^{-2}
13	*DPP4*	−1.7331	8.40×10^{-2}
14	*CXCL5*	−1.7268	2.36×10^{-2}
15	*CST2*	−1.7013	2.14×10^{-2}
16	*ANGPTL4*	−1.6559	6.90×10^{-2}
17	*TREM2*	−1.6256	5.36×10^{-3}
18	*ICAM1*	−1.5626	7.34×10^{-3}
19	*VEGFA*	−1.5174	3.43×10^{-2}
20	*ITGA2*	−1.4523	1.57×10^{-2}
21	*RORC*	−1.3843	3.01×10^{-2}
22	*COL11A1*	−1.3746	2.74×10^{-1}
23	*APOE*	−1.3746	2.66×10^{-2}
24	*GPC4*	−1.2827	5.27×10^{-2}
25	*GLS*	−1.2496	8.22×10^{-2}
26	*CDKN2B*	−1.2476	6.88×10^{-2}
27	*MMP7*	−1.2351	1.57×10^{-1}
28	*COL5A1*	−1.2189	1.24×10^{-1}
29	*LAMC2*	−1.2058	1.48×10^{-1}
30	*IFNGR2*	−1.1967	1.82×10^{-3}

Top 30 upregulated genes

No	Gene	log_2(FC)	*P* value
1	*HLA-DQA1*	2.0196	3.24×10^{-1}
2	*SFRP1*	1.4704	5.89×10^{-2}
3	*HLA-DRB5*	1.3970	5.80×10^{-1}
4	*SELE*	1.2616	6.17×10^{-2}
5	*EOMES*	1.2610	4.55×10^{-2}
6	*IL12RB2*	1.2470	2.81×10^{-2}
7	*TDO2*	1.2282	3.87×10^{-2}
8	*GZMK*	1.2006	1.44×10^{-1}
9	*TCL1A*	1.0877	2.07×10^{-1}
10	*CCR4*	1.0587	7.35×10^{-2}
11	*CXCL13*	1.0307	3.53×10^{-1}
12	*LCK*	1.0119	9.28×10^{-2}
13	*MAGEA3/A6*	1.0066	5.20×10^{-1}
14	*PLA2G2A*	0.9988	4.41×10^{-1}
15	*CD27*	0.9718	2.94×10^{-1}
16	*HLA-DQB1*	0.9370	5.59×10^{-1}
17	*GZMA*	0.9100	1.40×10^{-1}
18	*CCND2*	0.9043	2.99×10^{-1}
19	*IL34*	0.8977	1.18×10^{-1}
20	*CCR5*	0.8917	1.21×10^{-1}
21	*CD28*	0.8906	1.05×10^{-1}
22	*TNFSF18*	0.8738	1.42×10^{-1}
23	*SH2D1A*	0.8645	1.51×10^{-1}
24	*CXCR3*	0.8644	1.14×10^{-1}
25	*CCL19*	0.8616	4.27×10^{-1}
26	*NFATC2*	0.8355	1.01×10^{-1}
27	*TNFRSF17*	0.8281	4.38×10^{-1}
28	*TRAT1*	0.8100	1.63×10^{-1}
29	*XCL1/2*	0.8094	1.13×10^{-1}
30	*CXorf36*	0.7901	1.45×10^{-1}

Supplemental Figure 11. Differential gene expression between favorable and unfavorable TMEs. A volcano plot (top) shows $-\log_{10}$(*P* value) and $\log_2$(fold change [FC]) of gene expression levels for 726 genes evaluated by nCounter IO360, comparing favorable TME (N = 4] and unfavorable TME (N = 18). The top 30 upregulated genes (bottom left) and downregulated genes (bottom right) in favorable TME are listed with *P* values, ranked by the $\log_2$(FC).

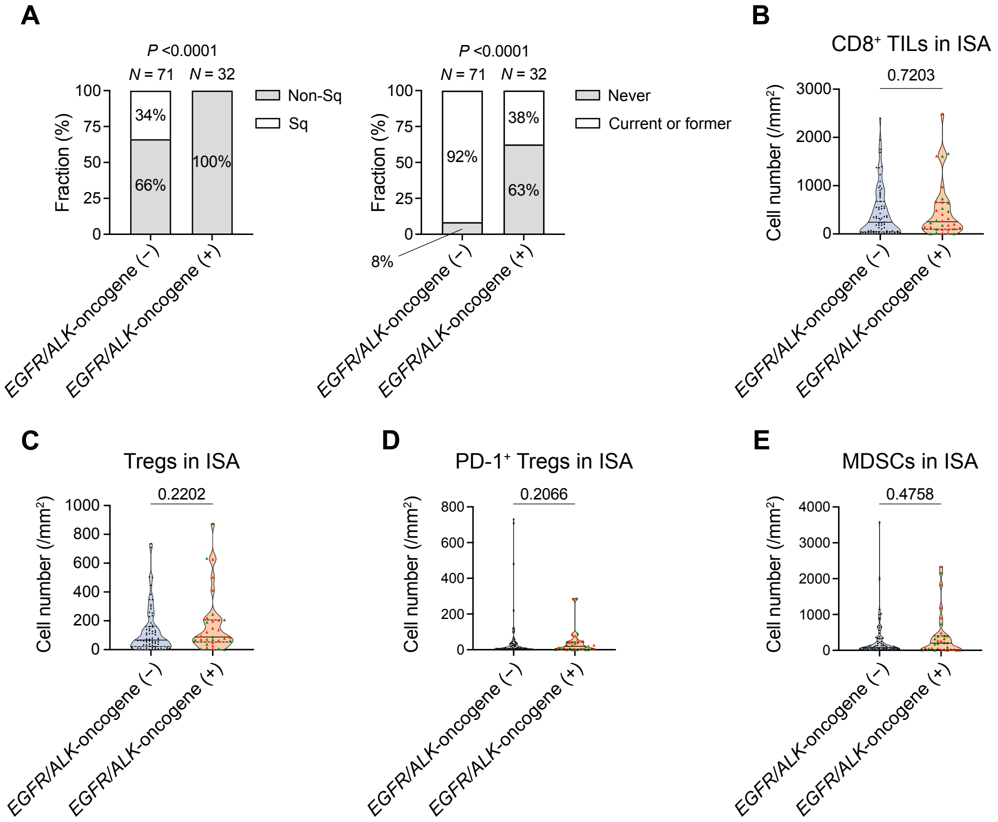

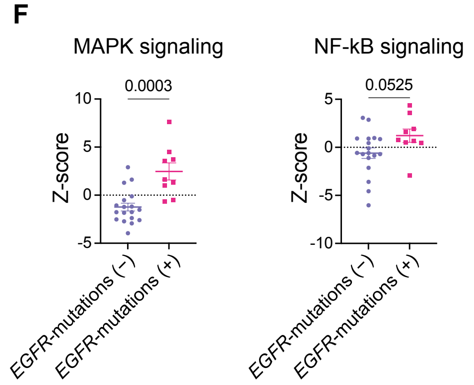

G

No	Gene	$\log_2$(FC)	*P* value	*q*-value
1	*S100A9*	−2.0941	2.37×10^{-3}	3.43×10^{-2}
2	*S100A8*	−2.0029	9.80×10^{-3}	6.97×10^{-2}
3	*IL18*	−1.9027	6.51×10^{-4}	2.21×10^{-2}
4	*SERPINB5*	−1.8984	6.13×10^{-2}	2.21×10^{-1}
5	*MMP1*	−1.7794	7.89×10^{-2}	2.55×10^{-1}
6	*CCND2*	−1.5929	7.27×10^{-3}	5.61×10^{-2}
7	*FCGR3A/B*	−1.4445	2.41×10^{-3}	3.43×10^{-2}
8	*C1QA*	−1.2689	6.59×10^{-3}	5.44×10^{-2}
9	*S100A12*	−1.2473	6.78×10^{-2}	2.30×10^{-1}
10	*VTCN1*	−1.2354	7.61×10^{-2}	2.50×10^{-1}
11	*SNCA*	−1.1929	1.08×10^{-2}	7.42×10^{-2}
12	*CXCL6*	−1.1765	2.55×10^{-2}	1.32×10^{-1}
13	*CCL3/L1*	−1.0981	1.07×10^{-2}	7.41×10^{-2}
14	*HLA-DOB*	−1.0576	3.49×10^{-2}	1.60×10^{-1}
15	*TLR8*	−1.0265	1.57×10^{-2}	9.21×10^{-2}
16	*LAMC2*	−0.9908	8.39×10^{-2}	2.57×10^{-1}
17	*SIRPA*	−0.9595	3.35×10^{-3}	3.87×10^{-2}
18	*IL16*	−0.9159	8.34×10^{-2}	2.57×10^{-1}
19	*ITGA6*	−0.9150	3.64×10^{-2}	1.62×10^{-1}
20	*FOSL1*	−0.9094	1.54×10^{-1}	3.71×10^{-1}
21	*IL1A*	−0.9045	5.80×10^{-2}	2.16×10^{-1}
22	*BLM*	−0.8931	4.64×10^{-3}	4.54×10^{-2}
23	*ITGB3*	−0.8798	8.38×10^{-2}	2.57×10^{-1}
24	*SREBF1*	−0.8651	1.15×10^{-3}	2.69×10^{-2}
25	*PECAM1*	−0.8583	6.13×10^{-2}	2.21×10^{-1}
26	*CENPF*	−0.8543	3.66×10^{-2}	1.62×10^{-1}
27	*FASLG*	−0.8156	8.35×10^{-2}	2.57×10^{-1}
28	*MXI1*	−0.8134	1.59×10^{-2}	9.21×10^{-2}
29	*IGF2R*	−0.8126	2.93×10^{-3}	3.73×10^{-2}
30	*WNT7B*	−0.7958	1.01×10^{-1}	2.94×10^{-1}

Supplemental Figure 12. Clinical features and minor TME features of *EGFR/ALK*-oncogene-positive NSCLC. (A) Tumor histology (left) and smoking status (right) compared between *EGFR/ALK*-oncogene–negative tumors (N = 71) and *EGFR/ALK*-oncogene–positive tumors (N = 32). **(B–E)** Densities of $CD8^+$ T cells (B), Tregs (C), PD-1^+ Tregs (D), and MDSCs (E) in ISA compared between *EGFR/ALK*-oncogene–negative tumors (N = 71) and *EGFR/ALK*-oncogene–positive tumors (N = 32). **(F)** Gene expression signature scores for MAPK and NF-kB signalling compared between *EGFR*-mutation-negative NSCLC (N = 18) and *EGFR*-mutation–positive NSCLC (N = 9). **(G)** Top 30 downregulated genes in *EGFR*-mutation-positive tumors from **Figure 10A** are listed with *q*-values and ranked by the $\log_2$(FC). Data are provided by violin plots in (B–E). Each dot represents each patient in the violin plots, where tumors with *EGFR*-mutations are colored red, while those with *ALK*-fusions are colored green. Bars indicate mean ± standard error of the mean in the scattered dot plots in (F). Fisher's exact test was used to determine the *P* value in (A). *P* values indicated above horizontal bars in (B–F) were determined using the Mann–Whitney U test, and *P* values in (G) were determined using unpaired t tests.

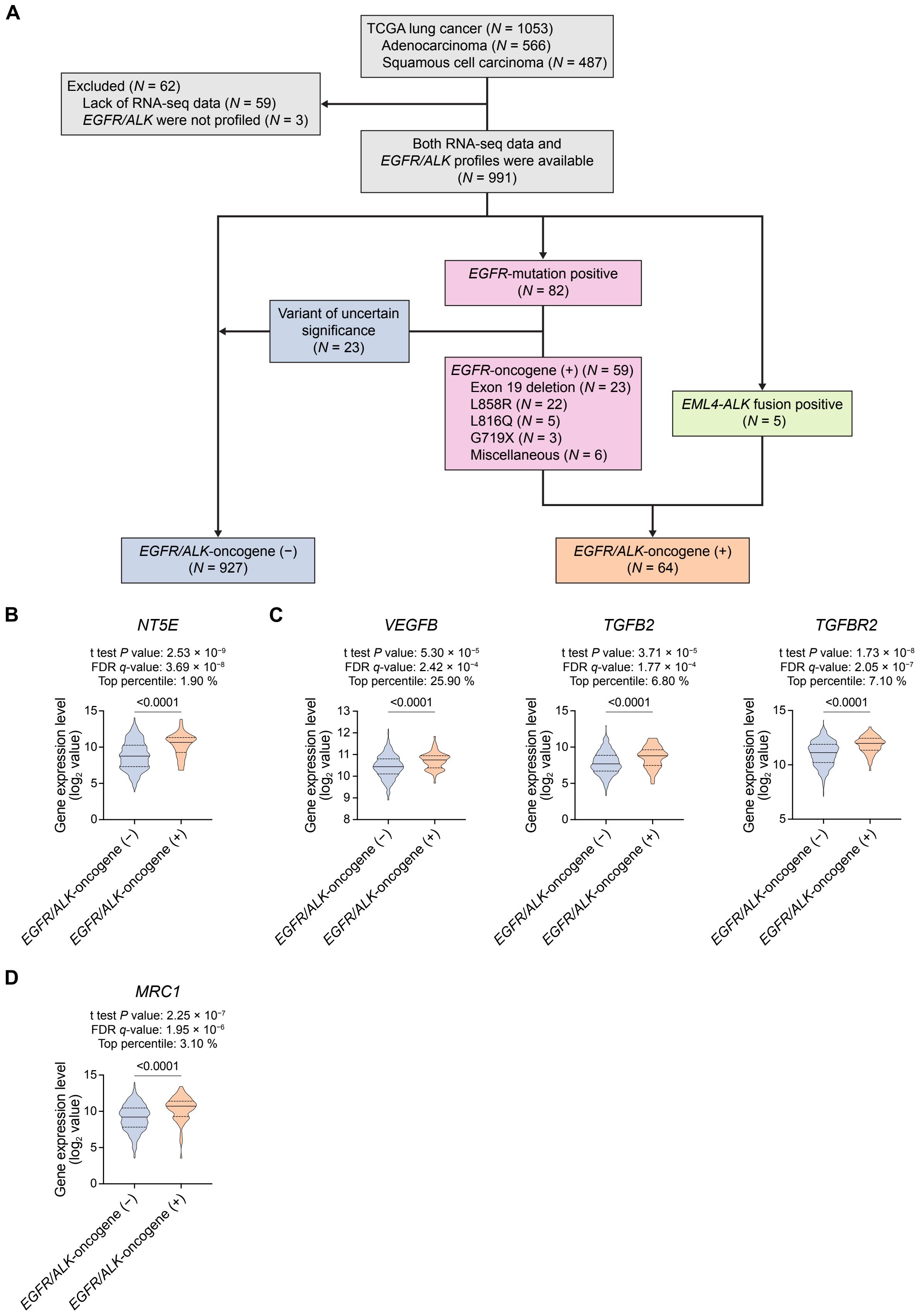
A
TCGA lung cancer (N = 1053)
Adenocarcinoma (N = 566)
Squamous cell carcinoma (N = 487)
Excluded (N = 62)
Lack of RNA-seq data (N = 59)
EGFR/ALK were not profiled (N = 3)
Both RNA-seq data and EGFR/ALK profiles were available (N = 991)
EGFR-mutation positive (N = 82)
Variant of uncertain significance (N = 23)
EGFR-oncogene (+) (N = 59)
Exon 19 deletion (N = 23)
L858R (N = 22)
L816Q (N = 5)
G719X (N = 3)
Miscellaneous (N = 6)
EML4-ALK fusion positive (N = 5)
EGFR/ALK-oncogene (−) (N = 927)
EGFR/ALK-oncogene (+) (N = 64)
B
NT5E
t test P value: 2.53 × 10⁻⁹
FDR q-value: 3.69 × 10⁻⁸
Top percentile: 1.90 %
C
VEGFB
t test P value: 5.30 × 10⁻⁵
FDR q-value: 2.42 × 10⁻⁴
Top percentile: 25.90 %
TGFB2
t test P value: 3.71 × 10⁻⁵
FDR q-value: 1.77 × 10⁻⁴
Top percentile: 6.80 %
TGFBR2
t test P value: 1.73 × 10⁻⁸
FDR q-value: 2.05 × 10⁻⁷
Top percentile: 7.10 %
D
MRC1
t test P value: 2.25 × 10⁻⁷
FDR q-value: 1.95 × 10⁻⁶
Top percentile: 3.10 %
<0.0001
Gene expression level (log2 value)
EGFR/ALK-oncogene (−)
EGFR/ALK-oncogene (+)

Supplemental Figure 13. TCGA dataset–based transcriptome analysis. (A) Flow diagram for case selection. **(B–D)** Gene expression levels of *NT5E* (B), *VEGFB*, *TGFB2*, *TGFBR2* (C), and *MRC1* (D) compared between *EGFR/ALK*-oncogene-positive and *EGFR/ALK*-oncogene-negative cases. Data are provided by violin plot in (B–D). Mann-Whitney U test was used to determine the *P* values shown on the horizontal bars. *P* values from unpaired *t* tests and FDR *q*-values were also provided. Ranking percentiles are determined based on $\log_2$(FC) across all evaluable 20,247 genes. Abbreviations: TCGA, The Cancer Genome Atlas; FDR, false discovery rate.

Supplemental Table 1. Characteristics of 81 patients treated with PD-1/PD-L1 inhibitors (ICI-cohort)

Characteristics	*N* = 81 (%)[a]
Median age (range), years	71 (43–89)
Sex	
Male	59 (73)
Female	22 (27)
ECOG performance status	
0–1	72 (90)
≥2	9 (10)
Smoking status[b]	
Current or former	69 (85)
Never	12 (15)
Diagnosis pattern at first line therapy	
Recurrence	17 (21)
De-novo IIIB-IV	64 (79)
CNS metastasis	
Yes	19 (23)
No	62 (77)
Histology	
Non-squamous[c]	57 (70)
Squamous	24 (30)
Biopsy site	
Primary	61 (75)
Metastatic[d]	20 (25)
EGFR mutation status[e]	
Ex19del	1 (1)
Ex19del + T790M	3 (4)
L858R	6 (7)
Wild	71 (88)
Next generation sequencing in *EGFR/ALK*-wild-type tumors	
Performed[f]	36 (44)
Not performed	35 (43)
PD-L1-TPS	
≥50 %	28 (34)
1–49 %	27 (33)
<1 %	19 (23)
Unknown	7 (9)
Regimen	
ICI monotherapy	52 (64)
Chemo + ICI combination	29 (36)
PD-1/L1 antibodies	
Nivolumab	25 (31)
Pembrolizumab	50 (62)
Atezolizumab	6 (7)
Anti-PD-1/L1 antibodies treatment lines	
1	59 (73)
2	11 (14)
≥3	11 (14)

[a]Percentages may not add up to 100 because of rounding. [b]Current smokers were defined as individuals who had smoked a cigarette within the previous year; former smokers were defined as those who had smoked ≥100 cigarettes but had quit >1 year before initiating anti-PD-1 antibody treatment; never-smokers were defined as individuals who had smoked <100 cigarettes. [c]Non-squamous histology includes adeno/adenosquamous (*N* = 50), sarcomatoid (*N* = 1), large cell (*N* = 2), and not-otherwise-specified (*N* = 4). [d]Metastatic sites include pleura (*N* = 7), lymph node (*N* = 5), brain (*N* = 3), bone (*N* = 2), adrenal (*N* = 1), lung (*N* = 1), and subcutaneous (*N* = 1). [e]Mutational status for the specimens with which mIHC was performed. [f]See **Supplemental Table 2** for details. Abbreviations: CNS, central nervous system; ex19del, exon 19 deletion.

Supplemental Table 2. Details and results of NGS performed in 36 *EGFR/ALK*-wild-type NSCLC

	N = 36 (%)[a]
NGS panel	
Foundation One CDx	9 (25)
Oncomine Dx Target Test Multi-CDx System	23 (64)
Ion AmpliSeq Colon and Lung Cancer Panel & Ion AmpliSeq RNA Fusion Lung Cancer Research Panel	3 (8)
Ion AmpliSeq™ Cancer Hotspot Panel v2 & FusionPlex Comprehensive Thyroid and Lung Kit	1 (3)
Positive for driver oncogenes	14 (39)
KRAS	9 (24)
G12C	2 (6)
G12C + G12F	1 (3)
G12D	3 (8)
G12V	1 (3)
G13R	1 (3)
Q61L	1 (3)
PIK3CA	2 (5)
E542K	1 (3)
H1047R	1 (3)
MET ex14del	1 (3)
BRAF V600E	1 (3)
ROS-1 fusion	1 (3)
Wild-type (no apparent driver-oncogenes)	22 (61)

[a]Percentages may not add up to 100 because of rounding. Abbreviations: ex14del, exon 14 deletion.

Supplemental Table 3. Definition of cell lineages based on multiplex immunohistochemistry staining pattern

Panel 1

Lineages	Identification biomarkers
Tumor cell	panCK^{+}CD45^{-}
Leukocyte	panCK^{-}CD45^{+}
T cell	panCK^{-}CD45^{+}CD3^{+}
CD8^{+} T cell	panCK^{-}CD45^{+}CD3^{+}CD8^{+}
Trm-like CD8^{+} T cell	panCK^{-}CD45^{+}CD3^{+}CD8^{+}CD39^{+}CD103^{+}
Regulatory T cell	panCK^{-}CD45^{+}CD3^{+}CD8^{-}NKp46^{-}FOXP3^{+}
T-helper 1 cell	panCK^{-}CD45^{+}CD3^{+}CD8^{-}FOXP3^{-}NKp46^{-}Tbet^{+}
NK cell	panCK^{-}CD45^{+}CD3^{-}NKp46^{+}
NKT cell	panCK^{-}CD45^{+}CD3^{+}CD8^{-}NKp46^{+}

Panel 2

Lineages	Identification biomarkers
Tumor cell	panCK^{+}CD45^{-}
Leukocyte	panCK^{-}CD45^{+}
B cell	panCK^{-}CD45^{+}CD3^{-}CD20^{+}
Myeloid cell	panCK^{-}CD45^{+}CD3^{-}CD20^{-}
Macrophage	panCK^{-}CD45^{+}CD3^{-}CD20^{-}CD68^{+}
M1 macrophage	panCK^{-}CD45^{+}CD3^{-}CD20^{-}CD68^{+}CD163^{-}CD206^{-}CD80^{+}
CD163^{+} M2 macrophage	panCK^{-}CD45^{+}CD3^{-}CD20^{-}CD68^{+}CD163^{+}
CD206^{+} M2 macrophage	panCK^{-}CD45^{+}CD3^{-}CD20^{-}CD68^{+}CD206^{+}
MDSC	panCK^{-}CD45^{+}CD3^{-}CD20^{-}CD33^{+}HLA-DPB1^{-}
PMN-MDSC	panCK^{-}CD45^{+}CD3^{-}CD20^{-}CD33^{+}HLA-DPB1^{-}CD66b^{+}
Dendritic cell (DC)	panCK^{-}CD45^{+}CD3^{-}CD20^{-}CD68^{-}HLA-DPB1^{+}
DC-LAMP^{+} DC	panCK^{-}CD45^{+}CD3^{-}CD20^{-}CD68^{-}HLA-DPB1^{+}DC-LAMP^{+}
CD80^{+} DC	panCK^{-}CD45^{+}CD3^{-}CD20^{-}CD68^{-}HLA-DPB1^{+}CD80^{+}
Fibroblast	panCK^{-}CD45^{-}FAP^{+}

Abbreviations: MDSC, myeloid-derived suppressor cell; HLA-DPB1, human leukocyte antigen–DPB1; PMN, polymorphonuclear.

Supplemental Table 4. Characteristics of patients in *EGFR/ALK*-wt, *EGFR*-mutant or *ALK*-fusion cohorts

Characteristics	Number of patients (%)[a]		
	EGFR/ALK-wt cohort ($N = 71$)	*EGFR*-mutant cohort ($N = 19$)	*ALK*-fusion cohort ($N = 13$)
Median age (range), years	71 (43–89)	73 (55–84)	72 (53–77)
Sex			
Male	56 (79)	6 (32)	6 (46)
Female	15 (21)	13 (68)	7 (54)
ECOG performance status			
0–1	62 (87)	19 (100)	13 (100)
≥2	9 (13)	0 (0)	0 (0)
Smoking status[b]			
Current or former	65 (92)	6 (32)	6 (46)
Never	6 (8)	13 (68)	7 (54)
Histology			
Non-squamous[c]	47 (66)	19 (100)	13 (100)
Squamous	24 (34)	0 (0)	0 (0)
EGFR mutation status[d]			
Ex19del	0 (0)	6 (32)	0 (0)
Ex19del + T790M	0 (0)	3 (16)	0 (0)
L858R	0 (0)	10 (53)	0 (0)
Wild	0 (0)	0 (0)	0 (0)
PD-L1-TPS			
≥50%	27 (38)	2 (11)	3 (23)
1–49%	26 (37)	4 (21)	0 (0)
<1%	12 (17)	10 (53)	3 (23)
Unknown	6 (8)	3 (16)	7 (54)

[a]Percentages may not add up to 100 because of rounding. [b]Current smokers were defined as individuals who had smoked a cigarette within the previous year; former smokers were defined as those who had smoked ≥100 cigarettes but had quit >1 year before initiating anti-PD-1 antibody treatment; never-smokers were defined as individuals who had smoked <100 cigarettes. [c]Non-squamous includes 72 adeno/adenosquamous, 1 sarcomatoid carcinoma, 2 large cell, and 4 not otherwise specified cases. [d]Mutational status for the specimens with which multiplexed immunohistochemistry was performed. Abbreviations: wt, wild-type.

Supplemental Table 5. Summary of representative pilot studies and comparison with the present study

Category	Reference Number	Journal name	Year	Author	Assay	Spatial analysis	Spatial measure	Cases evaluated for ICI efficacy	TIL	Trm-like CD8$^+$ TIL	TAM	CAF	Summary	Difference from our work
Conventional IHC	11	J Thorac Oncol.	2021	Shirasawa M et al	Conventional IHC<br>2 markers (CD8, PD-L1)	No	N/A	228	Yes	No	No	No	TME classification based on PD-L1 expression and CD8$^+$ TIL density in advanced NSCLC patients predicts anti–PD-1/PD-L1 therapy response, with PD-L1 High/TIL High tumors showing the most favorable outcomes.	This study classified tumors based on PD-L1 and CD8$^+$ TILs, while our work further incorporates spatial profiling of additional immune and stromal components including TAMs and CAFs.
H&E based AI detection	12	J Clin Oncol.	2022	Park S et al	H&E based AI detection<br>multiplex IHC<br>5 markers (CD3, CD8, FOXP3, CD68, Cytokeratin)	Yes	Tissue compartment evaluation	518<br>(mIHC cohort 99)	Yes	No	Yes	No	AI-powered analysis of TILs on whole-slide images using Lunit SCOPE IO stratifies NSCLC into three immune phenotypes that correlate with ICI outcomes and provide a complementary biomarker to PD-L1-TPS. 99 cases were subjected to mIHC for evaluating CD8$^+$ T cells, regulatory T cells, and macrophages.	This study highlighted immune phenotypes using AI-based H&E analysis, while our work complements this with high-plex mIHC and spatial profiling of tumor-reactive CD8$^+$ T cells, TAMs, and CAFs.
H&E based AI detection	13	JAMA Oncol.	2023	Rakaee M et al	H&E based AI detection	No	N/A	239<br>(Discovery cohort 446)	Yes	No	No	No	Machine learning was used to quantify TILs on standard H&E images from NSCLC patients treated with immunotherapy. High TIL levels (≥250 cells/mm^2) were independently associated with improved survival.	This study quantified TILs on H&E images to show prognostic value, while our work adds spatially resolved analyses of tumor-reactive CD8$^+$ T cells, TAMs, and CAFs to reveal mechanisms underlying ICI efficacy.
mIHC	28	Sci Rep	2022	Qin A et al	multiplex IF + image analysis<br>6 markers (CD3, CD8, CD163, PD-L1, FOXP3, Pancytokeratin)	Yes	Nearest neighbor distance	52	Yes	No	Yes	No	A spatial immune score combining PD-L1 expression, CTL–epithelial cell engagement, and CTL–helper T cell engagement outperforms PD-L1 alone in predicting ICI non-response in mNSCLC and can identify responders even in PD-L1–negative tumors.	This study focused on spatial interactions among T cells, while our work additionally considers other components such as TAMs and CAFs.
mIHC	29	EbioMedicine	2023	Ghiringhelli F et al	multiplex IHC + image analysis<br>2 markers (CD8, PD-L1)	Yes	Cell-cell distance	132<br>(Training cohort 133)	Yes	No	No	No	Immunoscore-IC (immune checkpoint), a digital pathology test quantifying CD8 and PD-L1 markers, is a powerful and superior predictor of anti-PD-1/PD-L1 immunotherapy efficacy in NSCLC patients, guiding treatment selection.	This study centered on CD8 and PD-L1 markers, while our work extends analyses to TAMs, CAFs, and tumor-reactive CD8$^+$ T cell subsets.
mIHC	30	J Thorac Oncol.	2021	Yeong J et al	multiplex IHC<br>6 markers (only CD39, CD8, and PD-L1 was described in the manuscript)	No	N/A	35	Yes	Yes	No	No	CD39$^+$CD8$^+$ T cell proportion and that this marker can serve as a potential biomarker that predicts response to ICB therapy in patients with NSCLC.	This study assessed CD39$^+$CD8$^+$ T cells in a smaller cohort, while our work includes larger-scale spatial profiling with additional cell types such as TAMs and CAFs.
mIHC	31	Cell Rep Med	2020	Corgnac S. et al	multiplex IF + image analysis<br>3 markers (CD8, CD103, cytokeratin)	Yes	Tissue compartment evaluation	86	Yes	Yes	No	No	CD103$^+$CD8$^+$ T cell density predicts better PFS in ICI-treated NSCLC.	This study linked CD103$^+$CD8$^+$ T cell density with outcome, while our work integrates spatial analyses.
mIHC	56	Nat Med	2018	Thommen DS et al	Flow cytometry<br>multiplex IHC + image analysis<br>2 markers (CD8, PD-1)	Yes	Co-localization analysis within TLS	21	Yes	Partially, Yes.<br>(only PD-1$^+$)	No	No	PD-1$^+$ T cells in NSCLC localize to TLSs and predict response to PD-1 blockade.	This study highlighted PD-1$^+$ T cells within TLSs, while our work expands to spatial profiling of tumor-reactive CD8$^+$ T cells, TAMs, and CAFs.
mIHC	57	Cell Rep Med	2025	Peyraud F et al	multiplex IF + image analysis<br>CAF cohort: 5 markers (PanCK, CD8, FAP, MYH11, αSMA, DAPI)<br>CD8$^+$ T cell cohort: 6 markers (CD8, PD-1, CD39, LAG-3, TIGIT, TIM-3)<br>Regulatory T cell cohort: 6 markers (CD4, CD8, CD20, FOXP3, ICOS, TIGIT)	Yes	Tissue compartment evaluation	CAF cohort: 77<br>CD8 cohort: 64<br>Treg cohort: 64	No	No	No	Yes	mature TLSs correlate with ICI benefit in NSCLC, while distinct CAF subsets promote resistance via immune exclusion and T cell exhaustion.	This study investigated CAF and immune subsets separately, while our work simultaneously analyzes spatial interactions among CD8$^+$ T cells, TAMs, and CAFs, including *EGFR*-mutant cohorts.
scRNA/FCM	65	Immunity	2023	Chow A et al	Single-cell RNA-seq<br>Flow cytometry	No	N/A	23	Yes	Yes	No	No	Single-cell RNA-seq and flow cytometry were used to characterize CD39$^+$CD8$^+$ TILs as neoantigen-reactive cells in NSCLC. In 23 ICI-treated Stage IV cases, higher CD39 expression was associated with longer PFS.	This study characterized CD39$^+$CD8$^+$ TILs using single-cell RNA-seq and flow cytometry, while our work complements this with spatial mIHC analyses incorporating TAMs, CAFs, and TME context.
mIHC	66	Front Oncol	2021	Li L et al	mIHC + image analysis<br>4 markers (CD8, CD68, CD163, PD-L1)	Yes	Tissue compartment evaluation	Stage IV: 20<br>Other stage: 13	No	No	Yes	No	CD8$^+$, CD8$^+$PD-L1$^+$ T cells, and M2 macrophages by mIHC predict ICI benefit; joint analysis enhances prognostic value.	This study evaluated CD8$^+$ T cells and M2 macrophages by mIHC, while our work expands the analysis to include CAFs and spatial profiling across broader cohorts.
mIHC	32	Lung Cancer	2022	Isomoto K and Haratani K et al<br>(Our prior work)	multiplex IHC + image analysis<br>Panel 1: 15 markers + hematoxylin<br>Panel 2: 16 markers + hematoxylin	Yes	Tissue compartment evaluation	PFS ≥12 months: 4<br>PFS <9 weeks: 4	Yes	Yes	Yes	Yes	Tumor-reactive CD39$^+$CD103$^+$CD8$^+$ T cells were associated with long-term ICI response in NSCLC. However, their presence alone was insufficient for durable tumor control, underscoring the role of heterogeneous TME profiles in acquired ICI resistance.	This study was based on a very small cohort only including 4 responders and 4 non-responders. All 4 responders developed disease progression after 1-year ICI response, precluding the possibility to evaluate TME features in association with persistent tumor control. Driver-oncogene-positive cancers were also excluded in this previous study, given its pilot nature. Nuanced analysis involving other TME features such as TAM, CAF, detailed CD8$^+$ TIL phenotypes including other co-inhibitory checkpoint molecules or Ki-67, as well as locations of Trm-like CD8$^+$ TILs was thus deferred to the current study.
mIHC	-	-	-	Isomoto K and Haratani K et al<br>(The current work)	multiplex IHC + image analysis<br>Panel 1: 15 markers + hematoxylin<br>Panel 2: 16 markers + hematoxylin	Yes	Tissue compartment evaluation	ICI cohort: 81<br>(including *EGFR* positive case 10)<br>Driver oncogene$^+$ cohort: 32	Yes	Yes	Yes	Yes	-	-

Abbreviations: AI, artificial intelligence; IF, immunofluorescence; MYH11, myosin heavy chain 11; αSMA, alpha smooth muscle actin DAPI, 4′,6-diamidino-2-phenylindole; TLS, tertiary lymphoid structure; ICOS, inducible T-cell co-stimulator.

Supplemental Table 6. Driver oncogenes examined by next generation sequencing panels used in this study

DNA (mutation)

AKT1	*ALK*	*BRAF*	*CTNNB1*	*EGFR*
ERBB2	*ERBB4*	*FGFR2*	*FGFR3*	*KRAS*
MET	*NRAS*	*PIK3CA*		

RNA (fusion)

ALK	*NTRK1*	*RET*	*ROS1*

Supplemental Table 7. Details of the multiplex immunohistochemistry panel adopted in this study

Panel 1

	Round 1	Round 2	Round 3	Round 4	Round 5	Round 6	Round 7	Round 8
Primary Ab	Hematoxylin	TIGIT	PD-1	CD45	GzmB	LAG-3	CD103	CD3
Supplier	Dako	Dianova	Abcam	ThermoFisher Invitrogen	Cell Marque	Abcam	Abcam	ThermoFisher Invitrogen
Clone/catalog#	S3301	TG1	NAT105	HI30	EP230	EPR4392	EPR4166(2)	SP7
Dilution		1:50	1:50	1:100	1:100	1:1000	1:500	1:150
Reaction	1 min	RT, 60 min	RT, 60 min	RT, 30 min	RT, 60 min	RT, 30 min	RT, 30 min	RT, 30 min
Histofine		Anti-mouse	Anti-mouse	Anti-mouse	Anti-rabbit	Anti-rabbit	Anti-rabbit	Anti-rabbit
Reaction		RT, 30 min	RT, 30 min	RT, 30 min	RT, 30 min	RT, 30 min	RT, 30 min	RT, 30 min
AEC reaction time		40 min	40 min	40 min	40 min	20 min	20 min	20 min

	Round 9	Round 10	Round 11	Round 12	Round 13	Round 14	Round 15	Round 16
Primary Ab	CD8	T-bet	FOXP3	Ki-67	TIM-3	NKp46	CD39	panCK
Supplier	Agilent Technologies	Abcam	ThermoFisher eBioscience	Abcam	Cell Signaling Technology	R&D Systems	Abcam	Leica Biosystems
Clone/catalog#	C8/144B	EPR9301	236A/E7	SP6	D5D5R	195314	EPR20267	AE1/AE3
Dilution	1:100	1:100	1:50	1:500	1:100	1:20	1:1000	1:200
Reaction	RT, 30 min	RT, 30 min	RT, 30 min	RT, 30 min	RT, 60 min	RT, 30 min	RT, 30 min	RT, 30 min
Histofine	Anti-mouse	Anti-rabbit	Anti-mouse	Anti-rabbit	Anti-rabbit	Anti-mouse	Anti-rabbit	Anti-mouse
Reaction	RT, 30 min	RT, 30 min	RT, 30 min	RT, 30 min	RT, 30 min	RT, 30 min	RT, 30 min	RT, 30 min
AEC reaction time	20 min	20 min	20 min	20 min	40 min	20 min	20 min	10 min

Panel 2

	Round 1	Round 2	Round 3	Round 4	Round 5	Round 6	Round 7	Round 8	Round 9
Primary Ab	Hematoxylin	CD68	CD45	CD80	CD206	CD163	CD33	CD66b	CD73
Supplier	Dako	Abcam	ThermoFisher Invitrogen	R&D Systems	Abcam	Abcam	Abcam	BioLegend	Cell Signaling Technology
Clone/catalog#	S3301	PG-M1	HI30	MAB14037711	Polyclonal	10D6	SP266	G10F5	D7F9A
Dilution		1:50	1:100	1:25	1:2500	1:100	1:50	1:200	1:50
Reaction	1 min	RT, 60 min	RT, 30 min	RT, 60 min	RT, 30 min	RT, 30 min	RT, 30 min	RT, 30 min	RT, 30 min
Histofine		Anti-mouse	Anti-mouse	Anti-mouse	Anti-rabbit	Anti-mouse	Anti-rabbit	Anti-mouse	Anti-rabbit
Reaction		RT, 30 min	RT, 30 min	RT, 30 min	RT, 30 min	RT, 30 min	RT, 30 min	RT, 30 min	RT, 30 min
AEC reaction time		40 min	40 min	20 min	20 min	20 min	20 min	20 min	20 min

	Round 10	Round 11	Round 12	Round 13	Round 14	Round 15	Round 16	Round 17
Primary Ab	B7-H3	HLA-DPB1	DC-LAMP	FAP	CD20	PNAd	CD3	panCK
Supplier	R&D Systems	Abcam	Merck Millipore	Abcam	Abcam	BioLegend	ThermoFisher Invitrogen	Leica Biosystems
Clone/catalog#	AF1027	EPR11226	16H11.2	EPR20021	L26	MECA-79	SP7	AE1/AE3
Dilution	1:40	1:5000	1:200	1:150	1:50	1:100	1:150	1:200
Reaction	RT, 30 min	RT, 30 min	RT, 30 min	RT, 30 min	RT, 30 min	RT, 30 min	RT, 30 min	RT, 30 min
Histofine	Anti-Goat polyclonal	Anti-rabbit	Anti-mouse	Anti-rabbit	Anti-mouse	Anti-rat	Anti-rabbit	Anti-mouse
Reaction	RT, 30 min	RT, 30 min	RT, 30 min	RT, 30 min	RT, 30 min	RT, 30 min	RT, 30 min	RT, 30 min
AEC reaction time	20 min	10 min	30 min	20 min	20 min	20 min	20 min	10 min

Abbreviations: Ab, antibody; AEC, alcohol-soluble peroxidase substrate 3-amino-9-ethyl-carbazole; RT, room temperature; min, minutes; PNAd, peripheral lymph node addressin.

Author contributions

KH conceptualized the study and designed experiments. KH and TT supervised mIHC experiments. KI and TT developed mIHC analysis software. KI and KH performed mIHC experiments. AI curated pathology specimen and assisted interpretation of mIHC samples. KH performed IO360 transcriptome experiments. KI and KH performed TCGA analysis. ST supported bioinformatic analyses of external validation RNA-sequencing datasets obtained via dbGaP. KI and YM collected data from clinical databases. KI and KH curated genomic data. MT, KY, and KS assisted curation of genomic data. KI and KH analyzed the data and visualized results. KI and KH wrote the manuscript. KH, TT, MT, KY, KT, TI, KS, K Nishio, AI, K Nakagawa, and HH provided resources and reviewed the manuscript. KH and K Nakagawa acquired funding and supervised the study. KI and KH contributed equally to this study as co-first authors. The contribution to performing laborious mIHC experiments and mIHC data preparation was mainly provided by KI; therefore, KI deserves the first position among the co-first authors. Analysis and interpretation of the mIHC data were jointly performed by KI and KH. KH also performed essential mIHC experiments including pilot studies required for establishing the entire platform, conducted most of the experiments and analyses related to the transcriptome, and wrote most parts of the original draft of the manuscript as a co-first author. KH guided KI and the overall study as the sole corresponding author. All authors reviewed and approved the final version.

Acknowledgements

We thank Mami Kitano, Yume Shinkai, Michiko Kitano, and Haruka Sakamoto of Kindai University, Kyoko Itoh of the Department of Pathology and Applied Neurobiology, Kyoto Prefectural University of Medicine, as well as Hiroshi Ogi and Saya Shibata of SCREEN Holdings Co. Ltd. for their technical support. We thank David A. Barbie of the Department of Medical Oncology, Dana-Farber Cancer Institute, for his administrative support.

Conflict-of-interest statement

KI has received honoraria from Eli Lilly Japan K.K. and Ono Pharmaceutical Co., Ltd., outside the submitted work; **KH** reports research funding regarding the submitted work from Osaka Cancer Society, KANAE Foundation for the Promotion of Medical Science, SGH Foundation, and YOKOYAMA Foundation for Clinical Pharmacology; other research funding from AstraZeneca K.K., Mochida Memorial Foundation, Nakatomi Foundation, Takeda Science Foundation, and The Osaka Medical Research Foundation for Intractable Disease; honoraria from AS ONE Corporation and AstraZeneca K.K., outside the submitted work; **ST** reports grant from Illumina, Inc.; honoraria from Illumina, Inc., outside the submitted work; **MT** has received honoraria from Chugai Pharmaceutical Co., Ltd., AstraZeneca K.K., Bristol-Myers Squibb Company, Novartis Pharma K.K., Ono Pharmaceutical Co., Ltd., Boehringer Ingelheim Japan Inc., Bayer Yakuhin Ltd., and Takeda Pharmaceutical Co., Ltd., outside the submitted work; **KY** reports grants from Daiichi Sankyo Co., Ltd. and Boehringer Ingelheim Japan Inc.; honoraria from Chugai Pharmaceutical Co., Ltd., Daiichi Sankyo Co., Ltd., and Boehringer Ingelheim Japan Inc.; patent royalty from Daiichi Sankyo Co., Ltd., outside the submitted work; **KT** has received honoraria from AstraZeneca K.K., Eisai Co., Ltd., Ono Pharmaceutical Co., Ltd., Chugai Pharmaceutical Co., Ltd., Taiho Pharmaceutical Co., Ltd., Novartis Pharmaceuticals K.K., Merck Biopharma Co., Ltd., Bristol-Myers Squibb Company, MSD K.K., Takeda Pharmaceutical Co., Ltd., and Kyowa Hakko Kirin Co., Ltd., outside the submitted work; **KS** has received honoraria from Life Technologies Japan Ltd., Chugai Pharmaceutical Co., Ltd., Takeda Pharmaceutical Co., Ltd., Qiagen, Inc., YODOSHA CO., LTD., and Nippon Kayaku Co., Ltd., outside the submitted work; **K Nishio** reports grants from Boehringer Ingelheim Japan Inc., West Japan Oncology Group, Thoracic Oncology Research Group, National Hospital Organization Osaka Minami Medical Center, Sysmex Corporation, Nichirei Biosciences Inc., Otsuka Pharmaceutical Co., Ltd, Hitachi, Ltd, and Eli Lilly Japan K.K.; consulting fees from SymBio Pharmaceuticals Ltd., Eli Lilly Japan K.K., and Otsuka Pharmaceutical Co., Ltd.; honoraria from Boehringer Ingelheim Japan Inc., AstraZeneca K.K., Chugai Pharmaceutical Co., Ltd., Novartis

Pharma K.K., MSD K.K., Bristol-Myers Squibb Company, Ono Pharmaceutical Co., Ltd., Pfizer Japan Inc., Guardant Health, Inc., Eli Lilly Japan K.K., Amgen K.K., Merck Biopharma Co., Ltd., Invitae Japan K.K., Nichirei Biosciences Inc., Yakult Honsha Co., Ltd., Takeda Pharmaceutical Co., Ltd., Janssen Pharmaceutical K.K., FUJIREBIO Inc., Daiichi Sankyo Co., Ltd., and Maruho Co., Ltd., outside the submitted work; **K Nakagawa** reports grants from MSD K.K., Takeda Pharmaceutical Co., Ltd., Eli Lilly Japan K.K., Bristol-Myers Squibb Company, Taiho Pharmaceutical Co., Ltd., Ono Pharmaceutical Co., Ltd., Janssen Pharmaceutical Co., Ltd., Kobayashi Pharmaceutical Co., Ltd., Shionogi & Co., Ltd., Amgen K.K., Chugai Pharmaceutical Co., Ltd., Boehringer Ingelheim Japan Inc., Nippon Kayaku Co., Ltd., AstraZeneca K.K., EPS International Co., Ltd., EP-CRSU Co., Ltd., CMIC Co., Ltd., Eisai Co., Ltd., Mochida Pharmaceutical Co., Ltd., Astellas Pharma Inc., GlaxoSmithKline K.K., SRL, Inc., Daiichi Sankyo Co., Ltd., IQVIA Services JAPAN K.K., Pfizer Japan Inc., Bayer Yakuhin Ltd., Otsuka Pharmaceutical Co., Ltd., PRA Health Sciences Inc., Novartis Pharma K.K., Mebix, Inc., Labcorp Development Japan K.K., Medical Research Support, Sanofi K.K., Syneos Health., Pfizer Japan Inc., Ascent Development Service, and Japan Clinical Research Operations; consulting fees from Eli Lilly Japan K.K. and Ono Pharmaceutical Co., Ltd.; honoraria from AstraZeneca K.K., MSD K.K., Ono Pharmaceutical Co., Ltd., Daiichi Sankyo Co., Ltd., Taiho Pharmaceutical Co., Ltd., Bristol Myers Squibb Co., Ltd., Global Health Consulting Japan Co., Ltd., Life Technologies Japan Ltd., TAIYO Pharma Co., Ltd., Medical Mobile Communications co., Ltd., Takeda Pharmaceutical Co., Ltd., Chugai Pharmaceutical Co., Ltd., Eli Lilly Japan K.K., Novartis Pharma K.K., Pfizer Japan Inc., M3, Inc., YODOSHA CO., LTD., Hisamitsu Pharmaceutical Co., Ltd., Incyte biosciences Japan, The Yomiuri Shimbun, Janssen Pharmaceutical K.K., Nippon Kayaku Co., Ltd., Bayer Yakuhin, Co., Ltd., Merck Biopharma Co., Ltd., Amgen K.K., Neo Communication, Otsuka Pharmaceutical Factory, Inc., Boehringer Ingelheim Japan Inc., CMIC Shift Zero K.K., CMIC Co., Ltd., and Japan Clinical Research Operations, outside the submitted work; **HH** reports grants from AstraZeneca K.K., Astellas Pharma Inc., Amgen K.K., MSD K.K., Ono Pharmaceutical Co., Ltd., Boehringer Ingelheim Co.,

Ltd., Novartis Pharma K.K., Pfizer Japan Inc., Bristol Myers Squibb Co., Ltd., Eli Lilly Japan K.K., Chugai Pharmaceutical Co., Ltd., Daiichi Sankyo Co., Ltd., Merck Biopharma Co., Ltd., Takeda Pharmaceutical Co., Ltd., Taiho Pharmaceutical Co., Ltd., Mochida Pharmaceutical Co., Ltd., Covance Japan Inc., AbbVie Inc, Shionogi & Co., Ltd., EPS Corporation., Syneos Health., Pfizer R&D Japan G.K., A2 Healthcare Corp., IQVIA Services JAPAN K.K., EP-CRSU Co., Ltd., Eisai Co., Ltd., Janssen Pharmaceutical K.K., CMIC Co., Ltd., Nippon Kayaku Co., Kyowa Hakko Kirin Co., Ltd, Bayer Yakuhin, Ltd, EPS International Co., Ltd., Otsuka Pharmaceutical Co., Ltd., Kobayashi Pharmaceutical Co., Ltd., PRA Health Sciences Inc., GlaxoSmithKline K.K., Sanofi K.K., SRL Medisearch Inc., Medical Research Support, Medpace Japan K.K., Mebix, Inc., Ascent Development Services, Labcorp Development Japan K.K., Japanese Gastric Cancer Association, West Japan Oncology Group, Thoracic Oncology Research Group, Clinical Research Support Center Kyushu, Japan Clinical Cancer Research Organization, Comprehensive Support Project for Oncological Research of Breast Cancer, and Japan Clinical Research Operations; honoraria from Amgen K.K., AstraZeneca K.K., Boehringer Ingelheim Japan Inc., Bristol-Myers Squibb Company, Chugai Pharmaceutical Co., Ltd., Daiichi Sankyo Co., Ltd., Eli Lilly Japan K.K., Guardant Health, Inc., Merck Biopharma Co., Ltd., MSD K.K., Novartis Pharmaceuticals K.K., Ono Pharmaceutical Co., Ltd., Janssen Pharmaceutical K.K., Pfizer Japan Inc., Takeda Pharmaceutical Co., Ltd., 3H Clinical Trial Inc., and Sysmex Corporation, outside the submitted work; All remaining authors have declared no conflicts of interest.
